# Phase Ia Clinical Evaluation of the Safety and Immunogenicity of the *Plasmodium falciparum* Blood-Stage Antigen AMA1 in ChAd63 and MVA Vaccine Vectors

**DOI:** 10.1371/journal.pone.0031208

**Published:** 2012-02-21

**Authors:** Susanne H. Sheehy, Christopher J. A. Duncan, Sean C. Elias, Sumi Biswas, Katharine A. Collins, Geraldine A. O'Hara, Fenella D. Halstead, Katie J. Ewer, Tabitha Mahungu, Alexandra J. Spencer, Kazutoyo Miura, Ian D. Poulton, Matthew D. J. Dicks, Nick J. Edwards, Eleanor Berrie, Sarah Moyle, Stefano Colloca, Riccardo Cortese, Katherine Gantlett, Carole A. Long, Alison M. Lawrie, Sarah C. Gilbert, Tom Doherty, Alfredo Nicosia, Adrian V. S. Hill, Simon J. Draper

**Affiliations:** 1 Centre for Clinical Vaccinology and Tropical Medicine, Churchill Hospital, Oxford, United Kingdom; 2 The Jenner Institute, University of Oxford, Oxford, United Kingdom; 3 University College London Clinical Research Facility, University College Hospital, London, United Kingdom; 4 Laboratory of Malaria and Vector Research, National Institute of Allergy and Infectious Diseases/National Institutes of Health, Rockville, Maryland, United States of America; 5 Clinical Biomanufacturing Facility, University of Oxford, Churchill Hospital, Oxford, United Kingdom; 6 Okairòs AG, Rome, Italy; 7 CEINGE, Naples, Italy; Queensland Institute of Medical Research, Australia

## Abstract

**Background:**

Traditionally, vaccine development against the blood-stage of *Plasmodium falciparum* infection has focused on recombinant protein-adjuvant formulations in order to induce high-titer growth-inhibitory antibody responses. However, to date no such vaccine encoding a blood-stage antigen(s) alone has induced significant protective efficacy against erythrocytic-stage infection in a pre-specified primary endpoint of a Phase IIa/b clinical trial designed to assess vaccine efficacy. Cell-mediated responses, acting in conjunction with functional antibodies, may be necessary for immunity against blood-stage *P. falciparum*. The development of a vaccine that could induce both cell-mediated and humoral immune responses would enable important proof-of-concept efficacy studies to be undertaken to address this question.

**Methodology:**

We conducted a Phase Ia, non-randomized clinical trial in 16 healthy, malaria-naïve adults of the chimpanzee adenovirus 63 (ChAd63) and modified vaccinia virus Ankara (MVA) replication-deficient viral vectored vaccines encoding two alleles (3D7 and FVO) of the *P. falciparum* blood-stage malaria antigen; apical membrane antigen 1 (AMA1). ChAd63-MVA AMA1 administered in a heterologous prime-boost regime was shown to be safe and immunogenic, inducing high-level T cell responses to both alleles 3D7 (median 2036 SFU/million PBMC) and FVO (median 1539 SFU/million PBMC), with a mixed CD4^+^/CD8^+^ phenotype, as well as substantial AMA1-specific serum IgG responses (medians of 49 µg/mL and 41 µg/mL for 3D7 and FVO AMA1 respectively) that demonstrated growth inhibitory activity *in vitro*.

**Conclusions:**

ChAd63-MVA is a safe and highly immunogenic delivery platform for both alleles of the AMA1 antigen in humans which warrants further efficacy testing. ChAd63-MVA is a promising heterologous prime-boost vaccine strategy that could be applied to numerous other diseases where strong cellular and humoral immune responses are required for protection.

**Trial Registration:**

ClinicalTrials.gov NCT01095055

## Introduction

An effective vaccine against the blood-stage of *Plasmodium falciparum* infection could significantly contribute to any future strategy for reducing malaria morbidity and mortality, limiting transmission and aiding disease eradication [1]. Although anti-disease and anti-parasitic immunity is naturally acquired against blood-stage infection following repeated exposure [2], replicating such immunity by vaccination has proved extremely difficult [3]. There have been recent reports of efficacy observed in retrospective/post-hoc analyses from Phase Ib safety and immunogenicity trials of a blood-stage vaccine [4] or one with a blood-stage component [5]. More encouragingly, significant strain-specific efficacy was also recently reported in a pre-specified secondary analysis of a Phase IIb trial of a mono-valent 3D7 strain apical membrane antigen 1 (AMA1) protein vaccine [6]. Of note, this vaccine also showed an encouraging signal in a prior Phase IIa controlled human malaria infection study [7]. However, despite these extensive efforts to date, no candidate blood-stage vaccine has been developed that has demonstrated statistically significant efficacy with regard to clinical outcome in a pre-specified primary endpoint analysis in a Phase IIa/b clinical trial designed to assess vaccine efficacy [3], [8]. The majority of such blood-stage vaccine candidates have traditionally focused on recombinant protein-in-adjuvant formulations with the aim of inducing growth inhibitory antibody responses against merozoite antigens involved in the erythrocyte invasion process [3]. However, increasing evidence suggests that T cells can also play an important contributory role in the mediation of immunity against blood-stage antigens [9], [10].

The mechanisms by which T cells could contribute to protective outcome *in vivo* in humans remain less well defined, particularly given the lack of MHC molecules necessary for antigen presentation on red blood cells (RBCs). One suggestion is that macrophages in the spleen, activated by cytokines from T helper 1 (Th1)-type CD4^+^ cells specific for blood-stage antigens, may enhance phagocytic clearance of infected RBCs [11], [12]. Another proposal is that CD4^+^ Th1 cells may bias the induction of cytophilic antibody subclasses from B cells that can in turn mediate anti-parasitic neutrophil respiratory burst activity (ADRB) [13] or antibody-dependent cellular inhibition (ADCI) [14] via monocytes. Alternatively, CD8^+^ T cell responses against blood-stage antigens could target late liver-stage parasite forms which also express classical ‘blood-stage’ antigens [15], [16], [17]. An effective blood-stage vaccine may therefore be required to induce strong cellular immunity that can act in concert with anti-parasite antibodies.

Recently, viral vectored vaccines encoding blood-stage antigens have been developed which can induce potent humoral and cellular immune responses in animal models [18]. Heterologous prime-boost immunization with human or simian adenovirus followed by the orthopoxvirus modified vaccinia virus Ankara (MVA) expressing the blood-stage antigen AMA1 is highly immunogenic for both antibodies and T cells in mice, rabbits [19] and rhesus macaques [20]. Although a long-standing subunit vaccine candidate antigen that is susceptible to strain-specific antibodies [21], AMA1 exhibits extreme polymorphism [22] which has proved a significant obstacle in the development of a broadly neutralizing antibody-inducing vaccine for use in endemic populations [6]. In the study reported here, the simian adenovirus and MVA vectors were designed to express an optimized transgene encoding two divergent alleles (3D7 and FVO) of AMA1 [19], [20]. These vectors, when used in heterologous prime-boost regimes in animal models, induced antibodies that mediate *in vitro* growth inhibition against both 3D7 and FVO strain *P. falciparum* parasites [19], [20]. Moreover, similar vaccines, encoding the orthologous gene, can confer blood-stage efficacy in the *P. chabaudi* rodent malaria model, which is dependent on vaccine-induced antibodies as well as AMA1-specific CD4^+^ T cells (Biswas *et al.*, submitted). T cell epitopes within AMA1 that elicit proliferative T cell responses have also been described in naturally-exposed individuals from Kenya [23], [24]. A recent Phase Ia study of a candidate human adenovirus serotype 5 (AdHu5) vaccine expressing *P. falciparum* AMA1 (3D7 strain allele) [25] was also shown to be immunogenic for AMA1-specific CD4^+^ and CD8^+^ T cells in malaria-naïve adults [26], [27]. However, concerns regarding pre-existing anti-vector immunity to human adenoviral serotypes [28], [29], and the inclusion of just one allele (3D7) in this vaccine formulation is likely to limit the widespread utility of this specific vaccine.

The replication-deficient chimpanzee adenovirus 63 (ChAd63) has been shown to be a safe, versatile and exceptionally immunogenic vector when administered in a heterologous prime-boost regimen with the attenuated orthopoxvirus MVA in two Phase Ia clinical trials in healthy malaria-naïve adults in the UK; one using vectors encoding the liver-stage antigen thrombospondin related adhesion protein fused to a multi-epitope string (ME-TRAP) (O'Hara *et al.*, J Infect Dis 2011 in press), and the other using the same vectors encoding the blood-stage antigen merozoite surface protein 1 (MSP1) [30]. Here we present the safety and immunogenicity results of the third ChAd63 vector to be trialled alone and in a prime-boost regimen with MVA. This Phase Ia trial utilized the ChAd63 and MVA vectors expressing an optimized bi-valent AMA1 insert designed to address antigen polymorphism, administered in a heterologous prime boost regimen to healthy malaria-naïve adults.

## Methods

### Objective

The objective of the study was to assess the reactogenicity and immunogenicity of ChAd63 AMA1 administered alone and with MVA AMA1 in healthy malaria-naïve adults.

### Participants

The study was conducted at the Centre for Clinical Vaccinology and Tropical Medicine, University of Oxford, Oxford, UK and the University College London Clinical Research Facility, London, UK. Healthy, malaria-naïve males and non-pregnant females aged 18–50 were invited to participate in the study. There was no selection of volunteers on the basis of pre-existing neutralizing antibodies (NAb) to the ChAd63 vector prior to enrolment, however NAb titers were subsequently assayed (see Supplementary Information S1 for the full list of inclusion and exclusion criteria).

### Study Design

**Figure 1:**
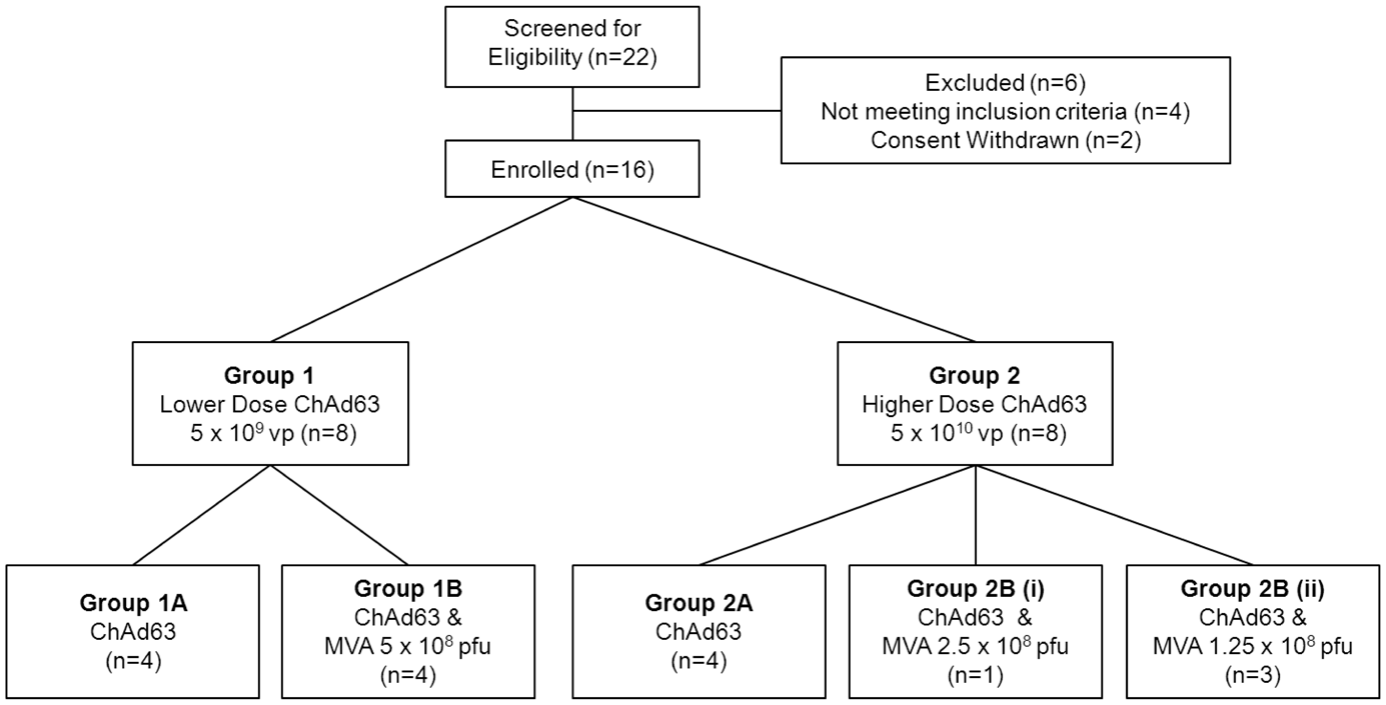
Flow chart of the study. All vaccinations were administered intramuscularly. ChAd63 AMA1 was administered on day 0 and MVA AMA1 on day 56. Six volunteers were excluded following screening for the following reasons: psychiatric morbidity; recurrent severe urticaria; elevated alanine aminotransferase (87 IU/L); unexplained microscopic haematuria and proteinuria; and withdrawal of consent (two individuals).

This was a Phase Ia open-label, non-randomized blood-stage malaria vaccine trial. The clinical trial protocol and supporting CONSORT checklist are available as Supplementary Information; see Protocol S1, Checklist S1 and Supplementary Information S1. Allocation to study groups (Figure 1) occurred at screening based on volunteer preference, as previously described [30]. Eight volunteers were vaccinated with 5×10^9^ viral particles (vp) ChAd63 AMA1 (diluted in 0.9% NaCl and administered in 300 µL) (groups 1A & 1B). Four of these volunteers were subsequently vaccinated in the opposite arm 56 days later with 5×10^8^ plaque forming units (pfu) MVA AMA1 undiluted and administered in 200 µL (group 1B). Another eight volunteers were vaccinated with 5×10^10^ vp ChAd63 AMA1 undiluted and administered in 300 µL (group 2A & 2B) and four of these were subsequently vaccinated 56 days later in the opposite arm with either 2.5×10^8^ pfu MVA AMA1 undiluted and administered in 100 µL (Group 2B(i), *n* = 1) or 1.25×10^8^ pfu MVA AMA1 undiluted and administered in 50 µL (Group 2B(ii), *n* = 3). Note the dose of MVA AMA1 was reduced following greater than expected reactogenicity when using 5×10^8^ pfu in Group 1B. All vaccinations were administered intramuscularly (IM) into the deltoid.

Volunteers attended clinical follow-up at days 2, 14, 28, 56 and 90 following ChAd63 AMA1 immunization in groups 1A and 2A and at days 2, 14, 28, 56, 58, 63, 84 and 140 following ChAd63-MVA AMA1 immunization in groups 1B and 2B. Safety assessments, including blood sampling for safety and immunology analysis at these visits were conducted as previously described [30]. A time window ranging between 1 and 14 days was allowed for vaccination and follow-up visits. Throughout the paper, study day refers to the nominal time point for a group and not the actual day of sampling.

The first volunteer to receive each dose of ChAd63 AMA1 was vaccinated in isolation. Following a review of reactogenicity in these individuals 48 hours post vaccination, the remaining volunteers in each group were enrolled. There was a 2 week interval prior to dose escalation of ChAd63 AMA1, during which time a scheduled review of safety data was conducted by the independent Local Safety Committee.

### Sample size

This was an observational and descriptive study to assess the safety and immunogenicity of ChAd63 AMA1 and MVA AMA1. The sample size (*n* = 16) was chosen to allow determination of the magnitude of the primary outcome measures, especially of serious and severe adverse events (AEs), rather than assessment of statistically significant differences between groups.

### Ethical & Regulatory Approval

The clinical trial protocol and associated documents were approved by the UK Gene Therapy Advisory Committee (GTAC 142). Clinical Trial Authorisation was granted by the United Kingdom Medicines and Healthcare Products Regulatory Agency (MHRA. Ref: 24821/85158/19/720). Vaccine use was authorized by the Genetically Modified Organisms Safety Committee (GMSC) of the Oxford Radcliffe Hospitals NHS Trust, UK (Reference number GM 462.09.42). All participants gave written informed consent prior to any study procedure being undertaken. The study was conducted according to the principles of the Declaration of Helsinki (2008) and the International Conference on Harmonization (ICH) Good Clinical Practice (GCP) guidelines. The Local Safety Committee provided safety oversight and GCP compliance was independently monitored by an external organization (Appledown Clinical Research Ltd, Great Missenden, UK).

### ChAd63 AMA1 and MVA AMA1 Vaccines

Generation of the recombinant vectors has been previously described [19], [20]. They were manufactured under Good Manufacturing Practice conditions by the Clinical Biomanufacturing Facility, University of Oxford (ChAd63 AMA1) and IDT Biologika, Rosslau, Germany (MVA AMA1). Briefly, ChAd63 AMA1 was generated in suspension HEK293 cells and purified by caesium chloride density-gradient centrifugation. MVA AMA1 was generated in chicken embryo fibroblasts (CEFs) and purified by sucrose density-gradient centrifugation. Each vaccine lot underwent comprehensive quality control analysis to ensure that the purity, identity and integrity of the virus met pre-defined specifications. Vaccine lots were stored at the clinical site at −80°C and the temperature was monitored. ChAd63 AMA1 vaccine stability was monitored by using an infectivity assay in HEK293 cells. The immuno-potency of the MVA AMA1 vaccine was confirmed by regular immunogenicity evaluation in mice.

To address the issue of AMA1 polymorphism, the transgene insert was designed as a bi-valent composite sequence from the *P. falciparum* blood-stage antigen AMA1 [19], [20]. Briefly, the sequence of the AMA1 insert contains from N- to C- terminus: the leader sequence from human tissue plasminogen activator (tPA) followed in-frame by the sequences encoding the ectodomain (amino acids 25–546) of *P. falciparum* (strain 3D7) AMA1 followed by the ectodomain plus C-terminal transmembrane region (amino acids 25–574) of *P. falciparum* (strain FVO) AMA1. These two regions represent two of the most diverged allelic variants of AMA1 (3D7 and FVO) which differ by 24 out of 622 amino acids [31], and were separated in the vaccine transgene insert by a flexible linker sequence (GGGPGGG) that has been used safely in other malaria constructs in Phase I/IIa trials [30], [32]. A number of amino acid substitutions (9 in the 3D7 allele and 10 in the FVO allele) (Tables S4 and S5) were also included to prevent potential N-linked glycosylation, as described elsewhere [20], [33].

### Safety

Volunteers in group 1 were observed for 2 hours post each immunization. Volunteers in group 2 were observed for 1 hour post each immunization. Volunteers were given a digital thermometer, injection site reaction measurement tool and symptom diary card to record their daily temperature, injection site reactions and solicited systemic AEs for 14 days following vaccination with ChAd63 AMA1 and 7 days following vaccination with MVA AMA1. Local and systemic reactogenicity was evaluated at subsequent clinic visits and graded for severity, outcome and association to vaccination as per the criteria outlined in Tables S1, S2, and S3. Blood was sampled at all visits post vaccination except days 2 and 58, and the full blood count with differential, platelet count and serum biochemistry (including electrolytes, urea, creatinine, bilirubin, alanine aminotransferase, alkaline phosphatase and albumin) measured.

### Peripheral Blood Mononuclear Cell (PBMC) and Serum Preparation

Blood samples were collected into lithium heparin-treated vacutainer blood collection systems (Becton Dickinson, UK). PBMC were isolated and used within 6 hours in fresh assays as previously described [30]. Excess cells were frozen in foetal calf serum (FCS) containing 10% dimethyl sulfoxide (DMSO) and stored in liquid nitrogen. For serum preparation, untreated blood samples were stored at 4°C and then the clotted blood was centrifuged for 5 min (1000×*g*). Serum was stored at −80°C.

### Peptides for T cell Assays

Peptides were purchased from NEO Peptide (Cambridge, MA, USA). The peptides, 20 amino acids (aa) in length and overlapping by 10 aa covered the entire AMA1 insert present in the viral vectored vaccines. Peptides were reconstituted in 100% DMSO at 50–200 mg/mL and combined into various pools for ELISPOT and flow cytometry assays. Additional peptide pools containing 5 peptides (tPA leader, 1 pool) or 21 peptides (“Vaccine” and “Native”, 1 pool for each) were prepared from pre-clinical peptide stocks (15mers overlapping by 10 aa) as previously described [20]. Peptides are listed in Tables S4 and S5.

### *Ex-vivo* interferon-γ (IFN-γ) ELISPOT

The kinetics and magnitude of the T cell response to AMA1 were assessed over time by *ex-vivo* IFN-γ ELISPOT following an 18–20 hour re-stimulation of PBMC with overlapping peptides spanning the entire AMA1 insert present in the viral vectored vaccines (Table S4). 20mer peptides overlapping by 10 amino acids (aa) were generated for the whole of the AMA1 vaccine insert present in the ChAd63 and MVA vaccines. Peptides were divided into pools containing up to 10 peptides per pool and were divided up according to whether they were 3D7 strain specific (3 pools, *n* = 24), FVO specific (3 pools, *n* = 24), common peptides (3 pools, *n* = 28), or FVO terminus peptides (1 pool, *n* = 7). Fresh PBMC were used in all ELISPOT assays using a previously described protocol [30], except that 50 µL/well AMA1 peptide pools (final concentration each peptide 5 µg/mL) were added to test wells, 50 µL/well R10 and DMSO control were added to negative un-stimulated wells, and 50 µL/well Staphylococcal enterotoxin B (SEB) (final concentration 0.02 µg/mL) plus phytohemagglutinin (PHA) (final concentration 10 µg/mL) was added to positive control wells. Spots were counted using an ELISPOT counter (Autoimmun Diagnostika (AID), Germany). Results are expressed as IFN-γ spot-forming units (SFU) per million PBMC. Background responses in un-stimulated control wells were almost always less than 20 spots, and were subtracted from those measured in peptide-stimulated wells. Responses are shown as the summed response to all the AMA1 peptide pools (unless otherwise stated). The tPA, “Vaccine” and “Native” responses (shown in Figure S4), represent data measured using single pools of peptides.

### Multiparameter Flow Cytometry

Cytokine secretion by PBMC was assayed by intracellular cytokine staining (ICS) followed by flow cytometry using a previously described protocol [30]. Briefly, frozen PBMC were re-stimulated for 18 hours in the presence of anti-human CD49d and CD28 (BD Biosciences) and CD107a. Re-stimulation for the final 16 hours was carried out in the presence of Brefeldin A (Sigma) and Monensin (Golgi Stop, BD Biosciences). Each sample was re-stimulated with either: 2 µg/mL SEB (positive control samples); a pool of all 83 peptides spanning the AMA1 vaccine antigen (see Table S4) at final concentration 2 µg/mL each peptide and 0.11% total DMSO concentration; 0.11% DMSO final concentration (un-stimulated peptide control sample); cryopreserved red blood cells infected with schizont/late trophozoite stage 3D7 strain *P. falciparum* parasites (iRBC) at 5×10^6^/mL; or uninfected red blood cell controls (uRBC) at 5×10^6^/mL prepared in the same manner. Cells were stained the next day using a Live/Dead marker, as well as for CD4, CD14, CD20, CD8α, CD3, IFN-γ, TNFα, and IL-2. Samples were analyzed using a LSRII Flow Cytometer (BD Biosciences) and FlowJo v8.8 (Tree Star Inc, USA). Dead cells, monocytes (CD14^+^), and B cells (CD20^+^) were excluded from the analysis. The Boolean gate platform was used with individual gates to create response combinations (Figure S6). Analysis and presentation of distributions was performed using SPICE v5.2, downloaded from http://exon.niaid.nih.gov/spice [34]. Background responses in un-stimulated peptide and uRBC control cells were subtracted from the AMA1 peptide and iRBC stimulated responses respectively.

### Total IgG ELISA

The recombinant 3D7 AMA1 protein was a gift from Dr Chetan Chitnis (ICGEB, New Delhi, India) and FVO AMA1 was a gift from Dr Mike Blackman (NIMR, London, UK) [19]. ELISAs were performed with these proteins using the same standardized methodology, as previously described for the MSP1_19_ antigen [30]. The reference serum used to generate the standard curve was prepared from adult Kenyan immune serum (a gift from Dr Faith H. Osier, KEMRI-Wellcome, Kilifi, Kenya). Antibodies against AMA1 (3D7 and FVO alleles) were also assayed by the GIA Reference Center (NIH, USA) as previously described [35], and these OD-based ELISA units were converted to antigen-specific µg/mL also as previously described [36].

### *In vitro* Assay of Growth Inhibitory Activity (GIA)

The ability of induced anti-AMA1 antibodies to inhibit growth of *P. falciparum* 3D7 and FVO strain parasites was assessed by a standardized GIA assay using purified IgG as previously described [36]. Briefly, each test IgG (10 mg/mL in a final test well) was incubated with synchronized *P. falciparum* parasites for a single growth cycle and relative parasitemia levels were quantified by biochemical determination of parasite lactate dehydrogenase.

### Measurement of NAb Titers to ChAd63

ChAd63 antibody neutralization assays were performed as previously described [37] except that a ChAd63 vector expressing secreted alkaline phosphatise (SEAP) [38], and GripTiteTM 293 MSR cells (Invitrogen R795-07) were used and cultured for one day prior to infection. The lowest serum dilution tested was 1∶18 and thus samples that did not give 50% neutralization in comparison to the control at this level are reported as “negative” or <1∶18.

### Statistical Analysis

Data were analyzed using GraphPad Prism version 5.03 for Windows (GraphPad Software Inc., California, USA). Geometric mean or median responses for each group are described. Significance testing of differences between two groups used the two-tailed Mann-Whitney U test or Wilcoxon signed rank test as appropriate. Correlations were analyzed using Spearman's rank correlation co-efficient (r_s_) for non-parametric data. A value of *P*<0.05 was considered significant.

## Results

### Study Recruitment

Recruitment took place between February 2010 and June 2010. Sixteen healthy malaria-naïve adult volunteers (10 female and 6 male) were enrolled, immunized and followed up (Figure 1). The mean age of volunteers was 30 years (range 18–48). Vaccinations began in March 2010 and all follow-up visits were completed by October 2010. All volunteers attended all visits as scheduled and completed the study.

### Safety and Reactogenicity

**Figure 2:**
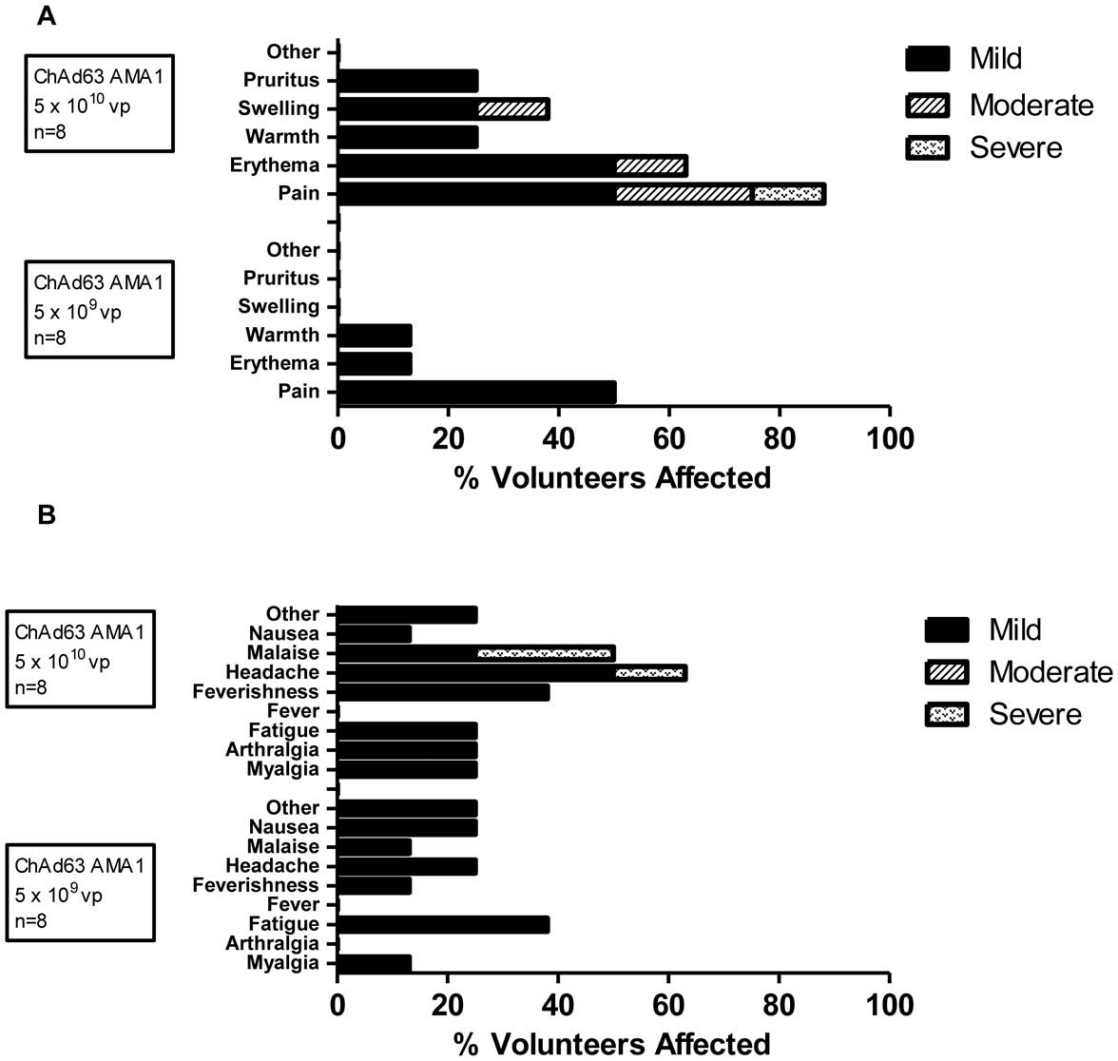
Local and systemic AEs deemed definitely, probably or possibly related to ChAd63 AMA1. Only the highest intensity of each AE per subject is listed. Data are combined for all AEs for all volunteers receiving the same vaccine at the stated dose. There were no immunization related serious AEs. (**A**) Local AEs post ChAd63 AMA1. (**B**) Systemic AEs post ChAd63 AMA1. ‘Other’ AEs post 5×10^9^ vp ChAd63 AMA1 included cough, coryzal symptoms, abdominal pain and dysmenorrhoea. ‘Other’ AE post 5×10^10^ vp ChAd63 AMA1 was coryzal symptoms. All ‘other’ AEs were considered possibly related to vaccination due to a temporal association.

**Figure 3:**
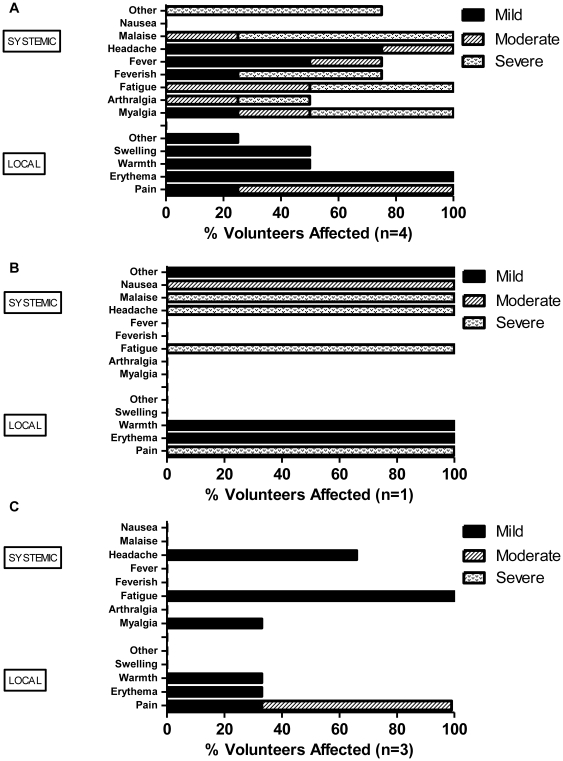
Local and systemic AEs deemed definitely, probably or possibly related to MVA AMA1. Only the highest intensity of each AE per subject is listed. Data are combined for all AEs for all volunteers receiving the same vaccine at the stated dose. There were no immunization related serious AEs. (**A**) Local and systemic AEs post 5×10^8^ pfu MVA AMA1. Local ‘other’ AE was mild bruising at vaccination site. Systemic ‘other’ AEs included two cases of rigor, one case of delirium, loss of appetite and chills. (**B**) Local and systemic AEs post 2.5×10^8^ pfu MVA AMA1. ‘Other’ AE post 2.5×10^8^ pfu MVA AMA1 was dizziness. (**C**) Local and systemic AEs post 1.25×10^8^ pfu MVA AMA1.

No unexpected or serious AEs occurred and no volunteers were withdrawn due to AEs. ChAd63 AMA1 demonstrated a good safety profile with the majority of AEs mild in severity (89%), all resolving completely, most (63%) within 48 hours (Figure 2). Overall, 12 out of 16 volunteers (75%) experienced one or more local AEs related to ChAd63 AMA1; these were mild with the exception of three cases of arm pain (two moderate and one severe), one case of moderate swelling and one case of moderate erythema, all occurring in the higher dose group (Figure 2A). 10 out of 16 volunteers (63%) also experienced at least one or more systemic AEs related to vaccination. These were all mild with the exception of two cases of severe malaise and one case of severe headache experienced by two volunteers in the higher dose group (Figure 2B). MVA AMA1 administered intramuscularly at the relatively high poxviral dose of 5×10^8^ pfu to the first four boosted volunteers (group 1B), was markedly more reactogenic than ChAd63 AMA1, with 3 out of 4 vaccinees (75%) experiencing a constellation of severe ‘flu-like’ systemic AEs (including malaise, myalgia, rigor, fatigue and feverishness) (Figure 3A). One of these volunteers described disorientation and ‘visual hallucinations’ whilst experiencing feverishness and a recorded fever of 37.8°C (AE defined as ‘delirium’). 92% of severe symptoms resolved completely within 48 hours of onset (one case of severe arthralgia took 72 hours to resolve). MVA expressing other malaria or HIV antigens has been used previously at doses equal to and higher than 5×10^8^ pfu without safety concern [39], [40], and such a dose was used because antibody induction in pre-clinical models has been shown to be dose dependent [41], [42]. However, after consultation with the Local Safety Committee, the dose of MVA AMA1 was subsequently halved to 2.5×10^8^ pfu for the fifth boosted volunteer but still resulted in severe local and systemic AEs (malaise and headache resulting in absenteeism from work) (group 2B (i), Figure 3B). The dose was therefore further reduced by half again to 1.25×10^8^ pfu for the remaining three volunteers (group 2B (ii), Figure 3C) and this now demonstrated an acceptable reactogenicity profile, typical of the MVA vector [39], [43], inducing only mild systemic AEs and mild or moderate local AEs.

### ChAd63-MVA AMA1 T cell immunogenicity assessed by *ex-vivo* IFN-γ ELISPOT

**Figure 4:**
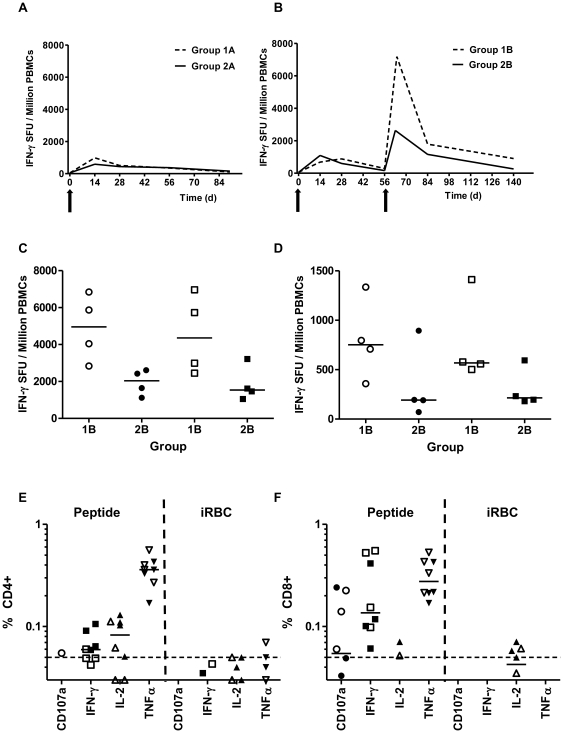
Cellular immunogenicity of ChAd63 AMA1 and ChAd63-MVA AMA1 immunization regimes. (**A**) Groups 1A and 2A and (**B**) groups 1B and 2B median e*x-vivo* IFN-γ ELISPOT responses in PBMC to the AMA1 insert (summed response across all the individual peptide pools). Individual responses are shown in Figure S1. Note due to allele-specific peptide overlap some responses can be potentially counted twice. Individual breakdowns of the ELISpot responses are shown in Figure S2, and data in Figure S3C show that on average one quarter to one third of the total summed response can be attributed to FVO allele-specific peptides. (**C**) Median and individual IFN-γ ELISPOT responses at day 63 and (**D**) at day 140 that are functional to the individual alleles 3D7 (circles) and FVO (squares). (**E,F**) PBMC from day 84 for group 1B (closed symbols) and group 2B (open symbols) were re-stimulated with a pool of AMA1 peptides or cryopreserved iRBCs. Individual data points and the median are shown for (**E**) the % CD4^+^ and (**F**) CD8^+^ T cells positive for CD107a, IFN-γ, IL-2 or TNFα. The dotted line indicates the 0.05% level and any response <0.03% is not shown.

Vaccination with ChAd63-MVA AMA1 induced antigen-specific T cell responses in all volunteers as measured by *ex-vivo* IFN-γ ELISPOT using 20mer peptides overlapping by 10aa, with individual responses shown in Figure S1 and median responses to the total vaccine insert shown for each group in Figure 4A and B. Following ChAd63 AMA1 prime, there was no significant difference between median responses in the higher dose group 2 in comparison to group 1 at the peak of the response on day 14 (median 921 [range 318–1366] vs 933 [range 298–2942] SFU/million PBMCs in groups 2 versus 1 respectively, *n* = 8 vs 8, *P* = 0.79 by Mann-Whitney test). Responses subsequently followed a classical T cell kinetic and contracted by day 56 (Figure 4A). Administration of MVA AMA1 at day 56 significantly boosted these responses in all volunteers as measured one week later on day 63 (Figure 4B). Due to the differences in dosage of MVA at this time point it is difficult to directly compare groups, however the volunteers in group 1B who all received 5×10^8^ pfu MVA AMA1 demonstrated a higher median response than those receiving the lower doses of MVA AMA1 (7186 [range 4372–10832] vs 2631 [range 2102–4500] SFU/million PBMCs). This was primarily due to two very strong responders in group 1B with peak post-boost responses in the region of 10,000 SFU/million PBMC. Analysis of the breakdown of the day 63 ELISPOT data showed that T cells were induced to both alleles equally; 3D7 (median 4959 [group 1B], 2036 [group 2B] SFU/million PBMC) and FVO (median 4359 [group 1B], 1539 [group 2B] SFU/million PBMC) (Figure 4C). Responses again contracted but were maintained above baseline at the end of the study period (day 140) with similar overall result to that observed at day 63 (Figure 4D).

### Breadth of the AMA1 T cell response

T cell responses in all volunteers were detected in multiple peptide pools spanning the entire AMA1 vaccine insert in the *ex-vivo* IFN-γ ELISPOT assay. Individual responses are shown according to magnitude of the response (Figure S2) or as a percentage of the total summed ELISPOT response (Figure S3). Irrespective of whether the responses are analyzed after the priming immunization with ChAd63 AMA1 or following the MVA AMA1 boosting immunization, the individual and median responses broadly mirror the composition of the vaccine antigen (Figure S2A). These data indicate that no single immuno-dominant region exists within the AMA1 transgene insert and importantly that responses were induced to epitopes contained within peptides whose sequence is common to both alleles. T cell responses were also measured to the tPA leader sequence (Figure S4A), and this showed the absence of any response induction against this mammalian sequence. A total of 19 amino acid substitutions were also included in the AMA1 transgene to remove sites of potential N-linked glycosylation. A pool of 21 previously described peptides (15mers overlapping by 10aa, Table S5) [20] was used that corresponds only to those peptides that vary between the native and vaccine AMA1 coding sequence. These peptides represent 21 out of 170 peptides (15mers) that are required to cover the whole AMA1 transgene insert, i.e. 12%. These two single pools were used here to measure responses following human vaccination. Responses to peptide pools for the vaccine versus native sequences of AMA1 showed a significantly stronger response to the vaccine pool for both day 14 (*n* = 16, *P* = 0.0007) and day 63 (*n* = 8, *P* = 0.008) as assessed by Wilcoxon signed rank test (Figure S4B).

### AMA1 T cell multi-functionality

Antigen-specific CD3^+^ T cell functionality was also assayed by ICS at the day 84 time-point (Figure 4E,F). Following peptide re-stimulation, detectable AMA1-specific CD3^+^ T cells consisted of a mixed CD4^+^ and CD8^+^ phenotype. It should be noted that the ELISPOT and ICS assays vary in methodology (including the use of multiple versus a single peptide pool respectively, as well as differences in peptide concentration, use of co-stimulatory antibodies and use of fresh versus frozen PBMC). Nevertheless, in agreement with the *ex-vivo* IFN-γ ELIspot data for this time-point (Figure 4A,B), reasonably comparable responses were seen in groups 1B and 2B for both T cell subsets. CD8^+^ T cells upregulated CD107a expression (marker of degranulation), and produced IFN-γ and TNFα but only negligible levels of IL-2. In comparison the CD4^+^ T cells produced high levels of TNFα, lower levels of IFN-γ and IL-2, but did not upregulate CD107a expression. However, following re-stimulation with cryopreserved red blood cells infected with schizont/late trophozoite stage 3D7 strain *P. falciparum* parasites (iRBCs), negligible (<0.05%) CD4^+^ or CD8^+^ T cell responses were evident above background uRBC re-stimulation. This is in contrast to results seen following ChAd63-MVA MSP1 immunization [30], whereby iRBC re-stimulation of PBMC resulted in comparable detection of CD4^+^ (but not CD8^+^) MSP1-specific T cell responses to those seen following peptide re-stimulation. This result may reflect the lower levels of AMA1 antigen present in iRBC preparations that is available for presentation to T cells (in comparison to the more abundant merozoite surface protein), or may in part be due to the aforementioned amino acid substitutions affecting T cell recognition of native parasite AMA1 antigen. Distinct populations of CD4^+^ and CD8^+^ T cells expressing 1+, 2+, 3+ or 4+ functional markers/cytokines were evident following a Boolean gate analysis (Figure S5).

### ChAd63-MVA AMA1 antibody immunogenicity assessed by ELISA

**Figure 5:**
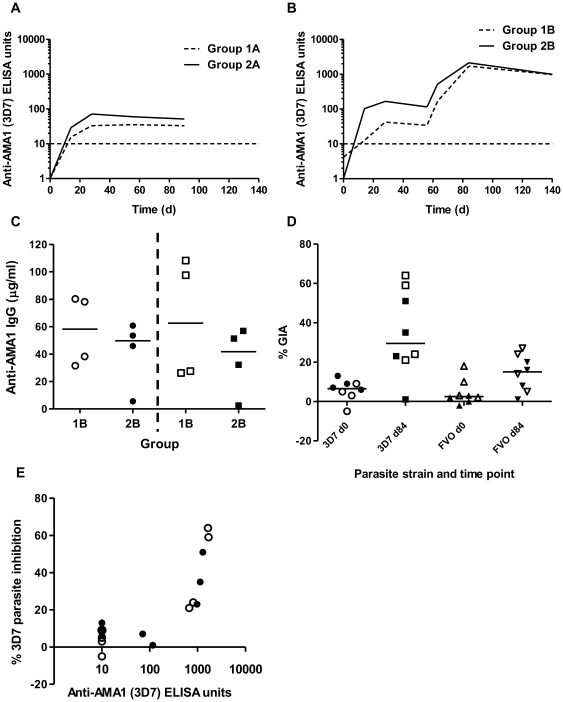
Antibody immunogenicity of ChAd63 AMA1 and ChAd63-MVA AMA1 immunization regimes. (**A**) Groups 1A and 2A and (**B**) groups 1B and 2B total IgG ELISA responses against 3D7 AMA1 as measured in the serum over time. The geometric mean response is shown for each group and individual responses are shown in Figure S7. The horizontal dotted line indicates the limit of detection of the assay. (**C**) AMA1-specific ELISA titers against 3D7 (circles) and FVO (squares) in µg/mL for group 1B (*n* = 4) and group 2B (*n* = 4) at the peak time-point (day 84). Individual data points and the median are shown. (**D**) % GIA against 3D7 and FVO parasites at day 0 and day 84 in group 1B (open symbols) and 2B (closed symbols). (**E**) Relationship between 3D7 strain % GIA and serum 3D7 AMA1-specific IgG ELISA titer at day 0 and day 84 in groups 1B (open symbols) and 2B (closed symbols).

The kinetics and magnitude of the serum IgG antibody response against the 3D7 allele of AMA1 were assessed over time by ELISA. AMA1-specific IgG was induced in all volunteers, with individual responses shown in Figure S7 and geometric mean (geomean) responses for each group in Figure 5A,B. Following the ChAd63 AMA1 prime, there were significantly stronger responses in the higher dose group 2 in comparison to group 1 at the peak of the response on day 28 (geomean titer 109 [range 48–196] vs 37 [range 18–192] AMA1 antibody units (AU) in groups 2 versus 1 respectively, *n* = 8 vs 8, *P* = 0.01 by Mann-Whitney test). Responses declined slowly but were maintained above the detection limit at day 90 in groups 1A and 2A. Administration of MVA AMA1 at day 56 significantly boosted these responses in all volunteers bar one (in group 2B(ii)), with serum IgG responses peaking four weeks later as measured on day 84. At this time-point, there was no significant difference (*P* = 0.68 by Mann-Whitney test) in the average AMA1-specific IgG responses in group 2B (5×10^10^ vp ChAd63 AMA1 prime and 2.5 or 1.25×10^8^ pfu MVA AMA1) in comparison to group 1B (5×10^9^ vp ChAd63 AMA1 prime and 5×10^8^ pfu MVA AMA1) (geomean titer 1709 [range 840–3370] vs 949 [range 84–2552] AMA1 AU respectively). Responses again declined over time but were maintained at high levels at the end of the study period (day 140) with again no significant difference (*P* = 1.00 by Mann-Whitney test) in the responses between groups 1B and 2B (geomean titer 971 [range 553–2683] vs 547 [range 90–1162] AMA1 AU respectively, *n* = 4 vs 4). Serum IgG responses against the FVO allele of AMA1 (which differs from the 3D7 allele by 24 amino acids) were also assessed by ELISA at day 84, and a strong correlation was evident between the responses against the two alleles (Spearman r = 0.97, *P* = 0.0004) (Figure S8A).

AMA1 AU quantified by standardized ELISA were also converted to concentration of antigen-specific antibodies against both alleles of AMA1 [36], with median concentrations of 58 µg/mL and 62 µg/mL anti-AMA1 3D7 and FVO IgG, respectively, in group 1B and 49 µg/mL and 41 µg/mL anti-AMA1 3D7 and FVO IgG, respectively, in group 2B (Figure 5C). The AMA1 specific IgG response induced functional GIA above baseline against both the 3D7 and FVO strains of *P. falciparum in vitro* (Figure 5D) and there was a significant correlation between the 3D7 and FVO % GIA (Spearman r = 0.90, *P* = 0.005) (Figure S8B). A sigmoidal relationship between 3D7 AMA1 AU and % 3D7 parasite GIA was also evident (Figure 5E), as described in other studies [20], [36].

### Pre-existing NAb titers against ChAd63 and immunogenicity

NAb titers against ChAd63 were assayed in the pre-immunization (day 0) serum of all volunteers but these were not used as an exclusion criterion. Five out of 16 volunteers were negative (titer<1∶18) and of the remaining 11 individuals all were low with the exception of two who had titers>1∶200 (the highest being 1∶369). There was no significant difference between the volunteers enrolled into Group 1 or Group 2 (*n* = 8 per group, *P* = 0.18, Mann Whitney test) (Figure S9A). There were also no significant correlations between these low level pre-existing NAb responses and ChAd63 AMA1 immunogenicity (as assessed by ELISPOT or ELISA at the peak of the priming response) in either dose group (Figure S9B,C).

## Discussion

In this Phase Ia study we have shown in healthy, malaria-naïve adult volunteers that a recombinant ChAd63-MVA heterologous prime-boost immunization regimen encoding AMA1 can induce functional antigen-specific antibody responses in addition to strong T cell responses. ChAd63 AMA1 demonstrated a good reactogenicity profile, similar to that seen consistently with the same doses of ChAd63 vectored vaccines encoding the pre-erythrocytic malaria antigen ME-TRAP (O'Hara *et al.*, J Infect Dis 2011 in press) and the blood-stage antigen MSP1 [30], supporting the growing body of evidence that this simian adenovirus vector is safe for clinical use. MVA expressing other malaria or HIV antigens has been used previously as a vaccine vector [28] at doses equal to and higher than the maximum dose used in this study without safety concern [39], [40]. The increased reactogenicity seen with the relatively high poxviral doses of MVA AMA1 used in this study (5×10^8^ pfu and 2.5×10^8^ pfu) may be a result of differences in viral titration methods or other factors. When MVA AMA1 was used at a dose of 1.25×10^8^ pfu, an acceptable reactogenicity profile was observed, without a significant compromise to vaccine antibody immunogenicity, although T cell responses tended to be higher following the higher boosting dose of MVA. A subsequent study has now shown this acceptable tolerability profile to be maintained following immunization of a further nine volunteers with the dosing regimen used here in group 2B(ii) (Sheehy *et al.*, manuscript in preparation).

The summed AMA1-specific IFN-γ T cell responses peaked at a median level of >6900 SFU/million PBMC in group 1B (ChAd63 5×10^9^ vp and MVA 5×10^8^ pfu), >2500 SFU/million in group 2B (ChAd63 5×10^10^ vp & MVA 1.25–2.5×10^8^ pfu). However, it remains possible that some T cell epitopes are duplicated within the 3D7- and FVO-specific 20mer peptides (because a number of these only differ by 1–2 amino acids) and thus the total sum may potentially count some responses twice. Examining the median summed individual responses to the two alleles in the vaccine, we found T cells were induced to both alleles equally; 3D7 (4959 SFU/million PBMC in group 1B and 2036 SFU/million PBMC in group 2B) and FVO (4359 SFU/million PBMC in group 1B and 1539 SFU/million PBMC in group 2B). The same result was observed at the late day 140 time-point. These allele-specific totals are not subject to any double counting error due to peptide homology between the 3D7 and FVO strains, and these median ELISPOT responses compare favourably with those induced by the same vectors and regimen for the ME-TRAP and MSP1 malaria antigens and are substantially greater than those reported for AMA1 protein-in-adjuvant vaccines [44], [45]. Responses induced after the ChAd63 AMA1 immunization also compared favourably with those recently reported following a single immunization of healthy adult US volunteers with 1×10^10^ vp of an AdHu5 vaccine encoding the 3D7 AMA1 antigen [27], although interestingly in this study the use of a higher dose (5×10^10^ vp) led to a significant reduction in T cell immunogenicity – an observation not seen here with the same dose of ChAd63.

A total of 19 amino acid substitutions, as described and used elsewhere [20], [33], were also included in the vaccine transgene to remove sites of potential N-linked glycosylation that could occur during *in vivo* mammalian expression of the AMA1 transgene. Such amino acid substitutions could affect T cell recognition of the native parasite AMA1 sequence if they occurred within recognized epitopes. Following immunization of rhesus macaques with the same ChAd63-MVA AMA1 vaccines these substitutions were shown to have a minimal effect following comparative re-stimulation with peptides representing these substitutions and which reflected about 12% of the total number of 15mer peptides required for complete coverage of the AMA1 transgene [20]. However following human vaccination here, responses to peptide pools for the vaccine versus native sequences of AMA1 showed a significantly stronger response to the vaccine pool, indicating amino acid substitutions included in the insert are likely to impact on the T cell recognition of native AMA1 parasite antigen in humans – an effect potentially observed in the peptide versus pRBC re-stimulation of PBMC. Similarly, it cannot be automatically assumed that cellular immune responses to the native AMA1 sequence would have been as strong as those observed to the modified vaccine sequence, although given responses were broadly strong to the remaining native sequence of the antigen (roughly 88% of the total), this seems unlikely. Future studies should thus extremely carefully address the potential advantages and disadvantages of such modifications in the context of vectored vaccine design. The discrepancy described here between the T cell data in rhesus macaques versus humans, indicates that the assessment of the positive versus negative impact(s) of such modifications at the preclinical stage may be extremely difficult.

The CD3^+^ T cell populations also consisted of a mixed CD4^+^ and CD8^+^ phenotype, and the cells expressed a range of functional cytokines/markers following AMA1 peptide re-stimulation *in vitro*. It remains a significant challenge to the field to establish the relevance of such *in vitro* measurements of T cell phenotypes to protective outcome against malaria *in vivo* in humans. Similar challenges are faced for *in vitro* assays of antibody function, and ultimately the development of an effective vaccine is required before such questions can be readily addressed. Nevertheless, the ChAd63-MVA vaccine platform, now tested here for the third time, represents a versatile delivery system for the reliable induction of high-level antigen-specific multi-functional T cell responses. The adverse reactogenicity profile of higher doses of MVA AMA1 means that future trials will use a maximum dose of 1.25×10^8^ pfu of this vaccine, which will limit median T cell responses to approximately 2000 SFU/million PBMC. However, these data indicate that exceptionally strong T cell responses (approximately 10,000 SFU/million PBMC) have the potential to be induced in humans by subunit vaccination, if the MVA vector could be further engineered or formulated to reduce reactogenicity whilst maintaining transgene potency.

In agreement with pre-clinical data in mice, rabbits and rhesus macaques [19], [20], this ChAd63-MVA prime-boost regimen also induced substantial AMA1-specific serum IgG antibody responses. When quantified by a standardized ELISA, the concentrations of AMA1-specific IgG induced at the peak of the response (median 49 µg/mL against 3D7 AMA1 in group 2B) were comparable to AMA1 protein vaccines formulated in Alum [46] or Montanide ISA720 [47], but three- to four-fold lower than Alum+CpG [45], [48]. As for T cell induction, the magnitude of these antigen-specific IgG antibody responses is also highly comparable to those seen with the ChAd63-MVA MSP1 regime [30]. Importantly, however, unlike for MSP1, ChAd63-MVA AMA1 induced moderate growth inhibition *in vitro* that correlated with total IgG ELISA titers. These levels were higher than those seen with a bi-valent (3D7 and FVO) AMA1 protein vaccine (AMA1-C1) formulated in Alum [48] and an AdHu5 vaccine encoding the 3D7 allele of AMA1 [27] (and assessed using the same assay of GIA), comparable to those when the vaccine was formulated in Montanide ISA720 [47], but lower than those seen with the same vaccine was formulated in Alum+CpG [45], [48]. This ability of the AMA1 antigen to induce functional GIA at lower levels of antigen-specific human IgG (in comparison to MSP1) is in agreement with previous data generated with protein-in-adjuvant vaccines [36]. Similarly, in this study *in vitro* inhibition levels of the FVO strain parasite were lower than those observed for 3D7, which is in agreement with previous clinical studies of the AMA1-C1 protein-in-adjuvant vaccine [47], [48], [49]. Interestingly, dose escalation of ChAd63 AMA1 from 5×10^9^ vp to 5×10^10^ vp led to a statistically significant increase in AMA1-specific serum IgG antibody responses but no significant increase in AMA1-specific IFN-γ T cell responses. The same finding was also seen with ChAd63 MSP1 [30]. A recent report of an AdHu5 vectored malaria vaccine showed the same trend for antibody responses but significantly reduced T cell responses at the highest dose used [27], whereas dose finding studies with the MRKAd5-gag HIV vaccine showed a similar plateauing of T cell responses (as assessed by *ex-vivo* IFN-γ ELISPOT) at the highest doses used [50]. The reason(s) for these quantitative T cell differences at high vaccine dose remain unclear and such responses may be vector as well as antigen-dependent. Similarly, functional or qualitative differences in T cell effector function following adenoviral vaccine dosing warrants further investigation.

In agreement with data relating to the use of the ChAd63 ME-TRAP vaccine in healthy UK volunteers (O'Hara *et al.*, J Infect Dis 2011 in press), there was also no apparent effect of low-level pre-existing anti-ChAd63 NAb responses on AMA1-specific cellular or humoral immune responses. This is also perhaps unsurprising given the relatively high doses of adenoviral vaccine used. Similar findings have recently been reported in healthy US adults following a single immunization with an AdHu5 vaccine encoding the *P. falciparum* circumsporozoite antigen [51].

The T cell and GIA data are reassuringly comparable to those seen in pre-clinical studies in mice [19], [42], [52] and macaques [20], supporting the importance of these models in pre-clinical vaccine optimization and development. The data also confirm that in comparison with leading AMA1 protein-in-adjuvant vaccines, the ChAd63-MVA vectored vaccine regimen in humans can induce comparable antibody titers, similar degrees of *in vitro* growth inhibition and markedly stronger CD4^+^ and CD8^+^ T cell responses including those against conserved sequences of AMA1. Since AMA1 is also expressed on sporozoites and during the liver-stage of infection [21], [53], the strong T cell responses, in particular CD8^+^ T cells, induced by ChAd63-MVA AMA1 may reduce the parasite inoculum released from the liver [15] and thus increase the potential for clinical efficacy at the blood-stage. It has been shown in animal models that immunization with multiple AMA1 alleles can focus B cell responses on more conserved eptiopes [54], and similarly the inclusion of conserved T cell epitopes in the vaccine insert described here (if capable of contributing to protective immunity in humans) may help to address the induction of effective immunity in the face of extensive parasite genetic variability. However, it remains for now unclear how effective these vaccines would be in the context of the marked AMA1 polymorphisms that are commonly seen in infecting parasites in the field [22] that are highly likely to affect the neutralizing ability of vaccine-induced antibodies. Future work should also examine whether the enhanced cell-mediated and humoral immunogenicity reported in pre-clinical studies (when protein-in-adjuvant formulations are combined with viral vectored vaccines [20], [41], [55]) can also be achieved in humans in clinical trials. Further studies are now essential to ascertain whether the addition of strong cellular immunity to such levels of antibody response can translate into significant vaccine efficacy against blood-stage infection in Phase IIa controlled human malaria challenge trials.

## Supporting Information

Checklist S1 **CONSORT Checklist.**
(DOC)

Figure S1 **Individual e** ***x-vivo*** **IFN-γ ELISPOT data.** ELISPOT responses to the AMA1 insert (summed response across all the individual peptide pools) are shown over time following immunization in (**A**) Group 1A (*n* = 4), (**B**) Group 1B (*n* = 4), (**C**) Group 2A (*n* = 4), and (**D**) Group 2B (*n* = 4). Individual responses are shown for each volunteer. MVA dosage explained in Figure 1. In Group 2B volunteer I = 2B (i), volunteers II–IV = 2B (ii). Note due to allele-specific peptide overlap some responses can be potentially counted twice. Individual breakdowns of the ELISpot responses are shown in Figure S2, and data in Figure S3C show that on average one quarter to one third of the total summed response can be attributed to FVO allele-specific peptides.
(PDF)

Figure S2 **Breakdown of e** ***x-vivo*** **IFN-γ ELISPOT data according to total response.** (**A**) The % of the amino acid sequence within the AMA1 vaccine insert that is attributable to each peptide pool is shown. (**B**) Data show the total response to each peptide pool within the AMA1 insert for each volunteer at day 14 after ChAd63 AMA1 immunization. (**C**) Data show the total response to each peptide pool within the AMA1 insert for each volunteer at the peak of the response at day 63 after ChAd63-MVA immunization. (**D**) The median IFN-γ response to each peptide pool within the AMA1 insert according to group (G1 or G2) and immunization regime (Ad = ChAd63, AdM = ChAd63-MVA) at the peak time-point (day 14 after ChAd63 prime and day 63 after ChAd63-MVA prime-boost). MVA dosage explained in Figure 1. Volunteer I = 2B (i), Volunteers II–IV = 2B (ii).
(PDF)

Figure S3 **Breakdown of e** ***x-vivo*** **IFN-γ ELISPOT data according to % response.** (**A**) Data show the % of the total response to each peptide pool within the AMA1 insert for each volunteer at day 14 after ChAd63 immunization. (**B**) Data show the % of the total response to each peptide pool within the AMA1 insert for each volunteer at the peak of the response (day 63) after ChAd63-MVA immunization. (**C**) The median response to peptide pool within the AMA1 insert according to group (G1 or G2) and immunization regime (Ad = ChAd63, AdM = ChAd63-MVA) at the peak time-point (day 14 after ChAd63 prime and day 63 after ChAd63-MVA prime-boost). MVA dosage explained in Figure 1. Volunteer I = 2B (i), Volunteers II–IV = 2B (ii).
(PDF)

Figure S4 **T cell responses to tPA sequence, Vaccine and Native pools.** (**A**) Data show *ex-vivo* IFN-γ ELISPOT responses to the tPA peptide pool for all volunteers at each time-point during the trial (*n* = 16 for d0–d56; *n* = 8 for d63–d140). Individual data points are shown as well as the median. Nominal cut-off for a significant response marked as 50 SFU/million PBMC. (**B**) Data show *ex-vivo* IFN-γ ELISPOT responses to peptide pools for Vaccine and Native sequences for all volunteers at the peak time-point after the prime (*n* = 16) and peak time-point after the boost (*n* = 8). Individual data points and median are shown. The responses shown represent data measured using a single pool of peptides (*n* = 21 peptides in each). In each graph: Group 1A (open circles); Group 1B (open squares); Group 2A (closed circles); Group 2B (closed squares).
(PDF)

Figure S5 **T cell multi-functionality following ChAd63-MVA AMA1 immunization.** The multi-functionality of the CD3^+^ T cell responses was assessed by polychromatic flow cytometry and ICS. Frozen PBMCs from day 84 were re-stimulated with a pool of AMA1 peptides and cells were stained as described. Gating strategy and representative plots are shown in Figure S6. The multi-functional compositions of the T cell responses following ChAd63-MVA immunization are shown for (**A**) CD4^+^ and CD8^+^ T cells in Group 1B following AMA1 peptide re-stimulation, and (**B**) CD4^+^ and CD8^+^ T cells in Group 2B following AMA1 peptide re-stimulation. Responses are grouped and colour-coded according to the CD4^+^ and CD8^+^ subsets, and the number of functions detected for each T cell population. Individual data points and median percentage of the parent CD4^+^ or CD8^+^ response (open bars) are shown for each of the functional populations indicated on the *x*-axis. The pie charts summarize the fractions of AMA1-specific CD4^+^ or CD8^+^ T cells that are positive for a given number of functions.
(PDF)

Figure S6 **Gating strategy for analysis of AMA1-specific T cell responses.** Representative flow cytometry plots are shown for the analysis of AMA1-specific T cell responses from volunteers immunized with ChAd63-MVA AMA1. (**A**) Initial gating used (from top left to bottom right) forward scatter area (FSC-A) versus forward scatter height (FSC-H) to remove doublet events and select singlet cells; then following this small lymphocytes were gated using FSC-A versus side scatter area (SSC-A); then live CD14^−^ CD20^−^ CD3^+^ cells were selected; then CD4 versus CD8 was used to select the total CD4^+^ CD8^−^ cell population and vice versa for the CD8^+^ CD4^−^ population. Cytokine (IFN-γ, IL-2 and TNFα) and CD107a gating using bivariate plots is shown for (**B**) CD4^+^ cells and (**C**) CD8^+^ cells. (**B**) Representative plots for un-stimulated (UNS), AMA1 peptide stimulated (AMA1), SEB, uRBC and iRBC stimulated samples are shown. IFN-γ (top row), IL-2 (second row), TNFα (third row) and CD107a (bottom row) for the CD8^−^ CD4^+^ T cell population were analyzed using bivariate plots. Percentages refer to the % of CD8^−^ CD4^+^ cells that express the specific cytokine or marker. Background responses in UNS or uRBC control cells were subtracted from the AMA1 and iRBC response respectively during the analysis. (**C**) Same analysis as in (**B**), except for the CD4^−^ CD8^+^ T cell population.
(PDF)

Figure S7 **Individual IgG ELISA data.** Total IgG ELISA responses against 3D7 PfAMA1 as measured in the serum over time following immunization in (**A**) Group 1A (*n* = 4), (**B**) Group 1B (*n* = 4), (**C**) Group 2A (*n* = 4), and (**D**) Group 2B (*n* = 4). MVA dosage explained in Figure 1. Volunteer I = 2B (i), Volunteers II–IV = 2B (ii).
(PDF)

Figure S8 **Relationship between total IgG ELISA titers and % GIA.** (**A**) Spearman's correlation of serum IgG ELISA titers against AMA1 for the 3D7 versus FVO alleles at day 84, *n* = 8. (**B**) Relationship between 3D7 strain % GIA and FVO strain % GIA using purified IgG at 10 mg/mL.
(PDF)

Figure S9 **Baseline NAb responses against ChAd63.** NAb titers against ChAd63 were assayed in the pre-immunization (day 0) serum of all volunteers. (**A**) Individual and median responses in Groups 1 and 2 are shown. The dotted lines indicate the level at which responses were classed as negative (titer<1∶18) and high (titers>1∶200). There was no significant difference between the volunteers enrolled into Group 1 or Group 2 (*n* = 8 per group, *P* = 0.18, Mann Whitney test). There were no significant correlations in either (**B**) Group 1 or (**C**) Group 2 between these low level pre-existing NAb titers and ChAd63 AMA1 immunogenicity as assessed by peak total summed AMA1 ELISPOT response at day 14 (blue) or anti-AMA1 (3D7) total IgG ELISA at day 28 (red). These were selected as the peak of the priming immune responses following ChAd63 AMA1 immunization. r_s_ = 0.36, *P* = 0.39 (ELISA) or r_s_ = −0.53, *P* = 0.20 (ELISPOT) in Group 1; and r_s_ = 0.32, *P* = 0.43 (ELISA) or r_s_ = −0.02, *P* = 0.98 (ELISPOT) in Group 2.
(PDF)

Protocol S1 **Trial Protocol.**
(PDF)

Supplementary Information S1 **Inclusion and exclusion criteria for volunteers in study.**
(DOCX)

Table S1 **Assessment of severity of AEs.**
(PDF)

Table S2 **Assessment of severity of local AEs.**
(PDF)

Table S3 **Assessment of relationship of AE to vaccination.**
(PDF)

Table S4 **AMA1 overlapping peptides.** 20mer peptides overlapping by 10 amino acids (aa) were generated for the whole of the AMA1 vaccine insert present in the ChAd63 and MVA vaccines. Peptides were divided into pools containing up to 10 peptides and were divided up according to whether they were 3D7 strain specific (3 pools, *n* = 24), FVO specific (3 pools, *n* = 24), common peptides (CP; 3 pools, *n* = 28), FVO terminus peptides (FVOT; 1 pool, *n* = 7). A single pool of tPA peptides (*n* = 5) was used and these were 15mers overlapping by 10aa. Amino acids that were substituted to prevent potential N-linked glycosylation are indicated in bold.
(PDF)

Table S5 **Vaccine versus Native peptides.** 15mer peptide sequences are shown, and pools indicated: V = sequence found in vaccine insert (*n* = 21); N = sequence found in native parasite (*n* = 21). Amino acids that were substituted to prevent potential N-linked glycosylation are highlighted in bold.
(PDF)
