# Supplementary material for: Phase Ia Clinical Evaluation of the Safety and Immunogenicity of the Plasmodium falciparum Blood-Stage Antigen AMA1 in ChAd63 and MVA Vaccine Vectors
Source: PLoS One. 2012 Feb 21;7(2):e31208. doi: 10.1371/journal.pone.0031208 (PMC3283618; doi:10.1371/journal.pone.0031208)
Supplement: Figure S1 — Individual e x-vivo IFN-γ ELISPOT data. ELISPOT responses to the AMA1 insert (summed response across all the individual peptide pools) are shown over time following immunization in (A) Group 1A (n = 4), (B) Group 1B (n = 4), (C) Group 2A (n = 4), and (D) Group 2B (n = 4). Individual responses are shown for each volunteer. MVA dosage explained in Figure 1. In Group 2B volunteer I = 2B (i), volunteers II–IV = 2B (ii). Note due to allele-specific peptide overlap some responses can be potentially counted twice. Individual breakdowns of the ELISpot responses are shown in Figure S2, and data in Figure S3C show that on average one quarter to one third of the total summed response can be attributed to FVO allele-specific peptides. (PDF) [file pone.0031208.s002.pdf]

Figure S1

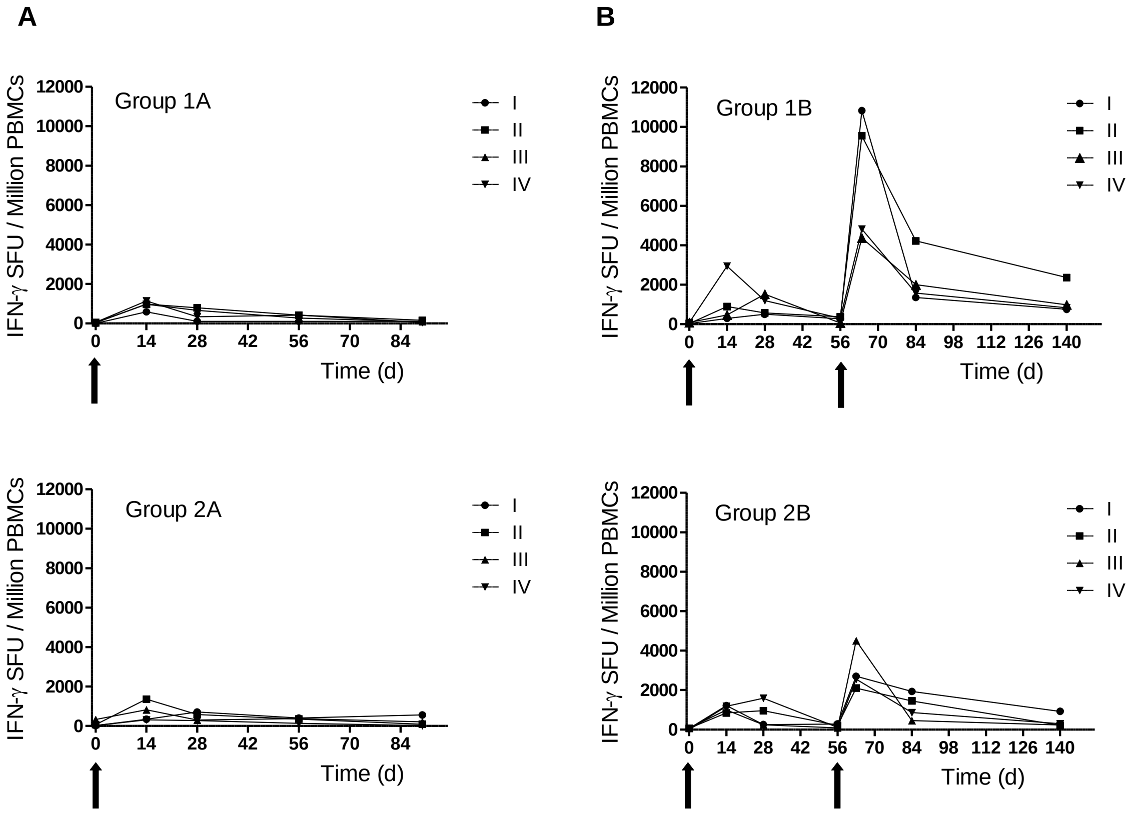

Figure S1. Individual *ex-vivo* IFN-γ ELISPOT data. ELISPOT responses to the AMA1 insert (summed response across all the individual peptide pools) are shown over time following immunization in **(A)** Group 1A (*n* = 4), **(B)** Group 1B (*n* = 4), **(C)** Group 2A (*n* = 4), and **(D)** Group 2B (*n* = 4). Individual responses are shown for each volunteer. MVA dosage explained in Figure 1. In Group 2B volunteer I = 2B (i), volunteers II-IV = 2B (ii). Note due to allele-specific peptide overlap some responses can be potentially counted twice. Individual breakdowns of the ELISpot responses are shown in Figure S2, and data in Figure S3C show that on average one quarter to one third of the total summed response can be attributed to FVO allele-specific peptides.
