# Supplementary material for: Phase Ia Clinical Evaluation of the Safety and Immunogenicity of the Plasmodium falciparum Blood-Stage Antigen AMA1 in ChAd63 and MVA Vaccine Vectors
Source: PLoS One. 2012 Feb 21;7(2):e31208. doi: 10.1371/journal.pone.0031208 (PMC3283618; doi:10.1371/journal.pone.0031208)
Supplement: Figure S2 — Breakdown of e x-vivo IFN-γ ELISPOT data according to total response. (A) The % of the amino acid sequence within the AMA1 vaccine insert that is attributable to each peptide pool is shown. (B) Data show the total response to each peptide pool within the AMA1 insert for each volunteer at day 14 after ChAd63 AMA1 immunization. (C) Data show the total response to each peptide pool within the AMA1 insert for each volunteer at the peak of the response at day 63 after ChAd63-MVA immunization. (D) The median IFN-γ response to each peptide pool within the AMA1 insert according to group (G1 or G2) and immunization regime (Ad = ChAd63, AdM = ChAd63-MVA) at the peak time-point (day 14 after ChAd63 prime and day 63 after ChAd63-MVA prime-boost). MVA dosage explained in Figure 1. Volunteer I = 2B (i), Volunteers II–IV = 2B (ii). (PDF) [file pone.0031208.s003.pdf]

Figure S2

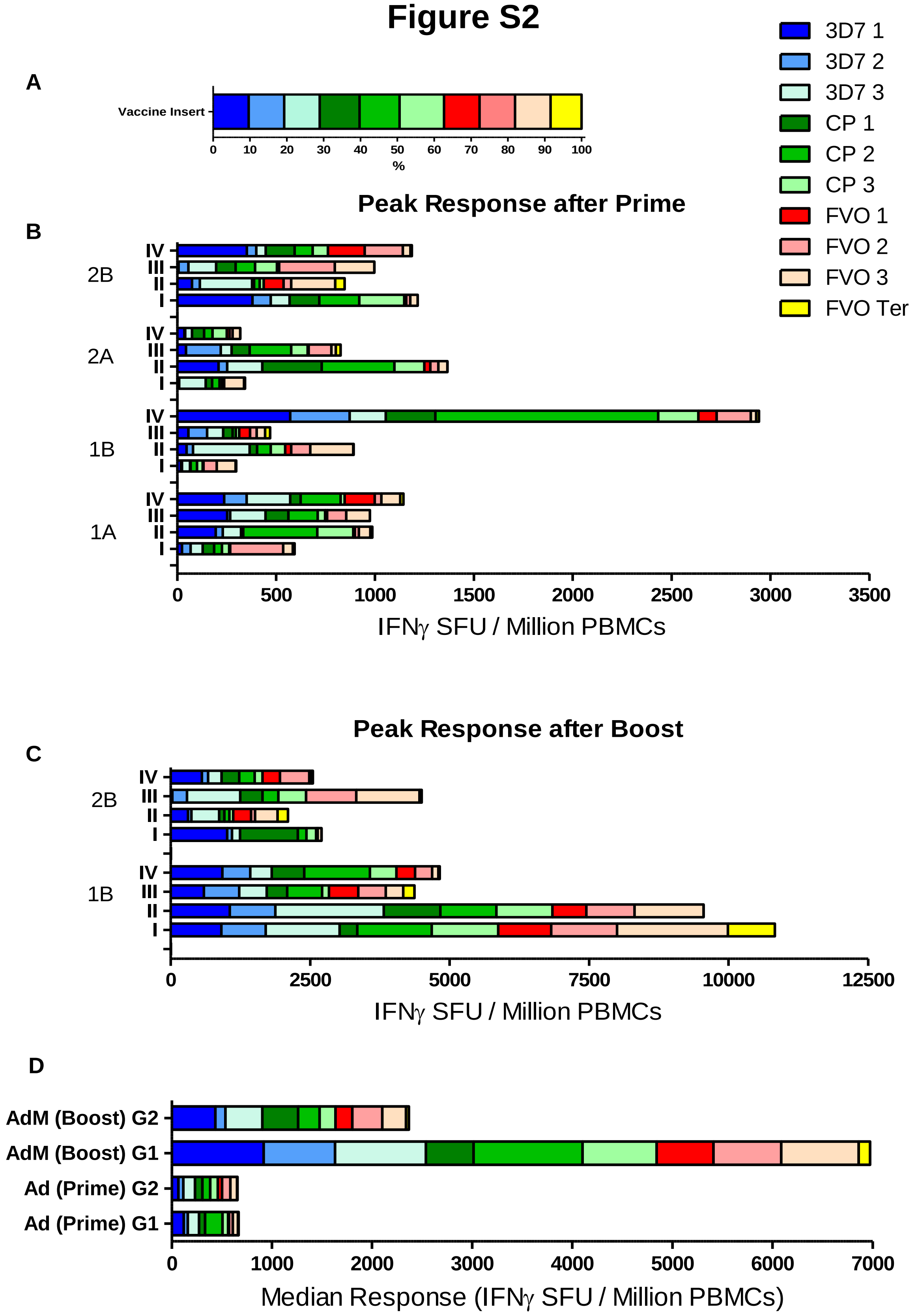

A
Vaccine Insert
0
10
20
30
40
50
60
70
80
90
100
%
3D7 1
3D7 2
3D7 3
CP 1
CP 2
CP 3
FVO 1
FVO 2
FVO 3
FVO Ter
B
Peak Response after Prime
2B
2A
1B
1A
IV
III
II
I
0
500
1000
1500
2000
2500
3000
3500
IFNγ SFU / Million PBMCs
C
Peak Response after Boost
2B
1B
IV
III
II
I
0
2500
5000
7500
10000
12500
IFNγ SFU / Million PBMCs
D
AdM (Boost) G2
AdM (Boost) G1
Ad (Prime) G2
Ad (Prime) G1
0
1000
2000
3000
4000
5000
6000
7000
Median Response (IFNγ SFU / Million PBMCs)

Figure S2. Breakdown of e*x-vivo* IFN-γ ELISPOT data according to total response. (A) The % of the amino acid sequence within the AMA1 vaccine insert that is attributable to each peptide pool is shown. **(B)** Data show the total response to each peptide pool within the AMA1 insert for each volunteer at day 14 after ChAd63 AMA1 immunization. **(C)** Data show the total response to each peptide pool within the AMA1 insert for each volunteer at the peak of the response at day 63 after ChAd63-MVA immunization. **(D)** The median IFN-γ response to each peptide pool within the AMA1 insert according to group (G1 or G2) and immunization regime (Ad = ChAd63, AdM = ChAd63-MVA) at the peak time-point (day 14 after ChAd63 prime and day 63 after ChAd63-MVA prime-boost). MVA dosage explained in Figure 1. Volunteer I = 2B (i), Volunteers II-IV = 2B (ii).
