# Supplementary material for: Phase Ia Clinical Evaluation of the Safety and Immunogenicity of the Plasmodium falciparum Blood-Stage Antigen AMA1 in ChAd63 and MVA Vaccine Vectors
Source: PLoS One. 2012 Feb 21;7(2):e31208. doi: 10.1371/journal.pone.0031208 (PMC3283618; doi:10.1371/journal.pone.0031208)
Supplement: Figure S3 — Breakdown of e x-vivo IFN-γ ELISPOT data according to % response. (A) Data show the % of the total response to each peptide pool within the AMA1 insert for each volunteer at day 14 after ChAd63 immunization. (B) Data show the % of the total response to each peptide pool within the AMA1 insert for each volunteer at the peak of the response (day 63) after ChAd63-MVA immunization. (C) The median response to peptide pool within the AMA1 insert according to group (G1 or G2) and immunization regime (Ad = ChAd63, AdM = ChAd63-MVA) at the peak time-point (day 14 after ChAd63 prime and day 63 after ChAd63-MVA prime-boost). MVA dosage explained in Figure 1. Volunteer I = 2B (i), Volunteers II–IV = 2B (ii). (PDF) [file pone.0031208.s004.pdf]

Figure S3

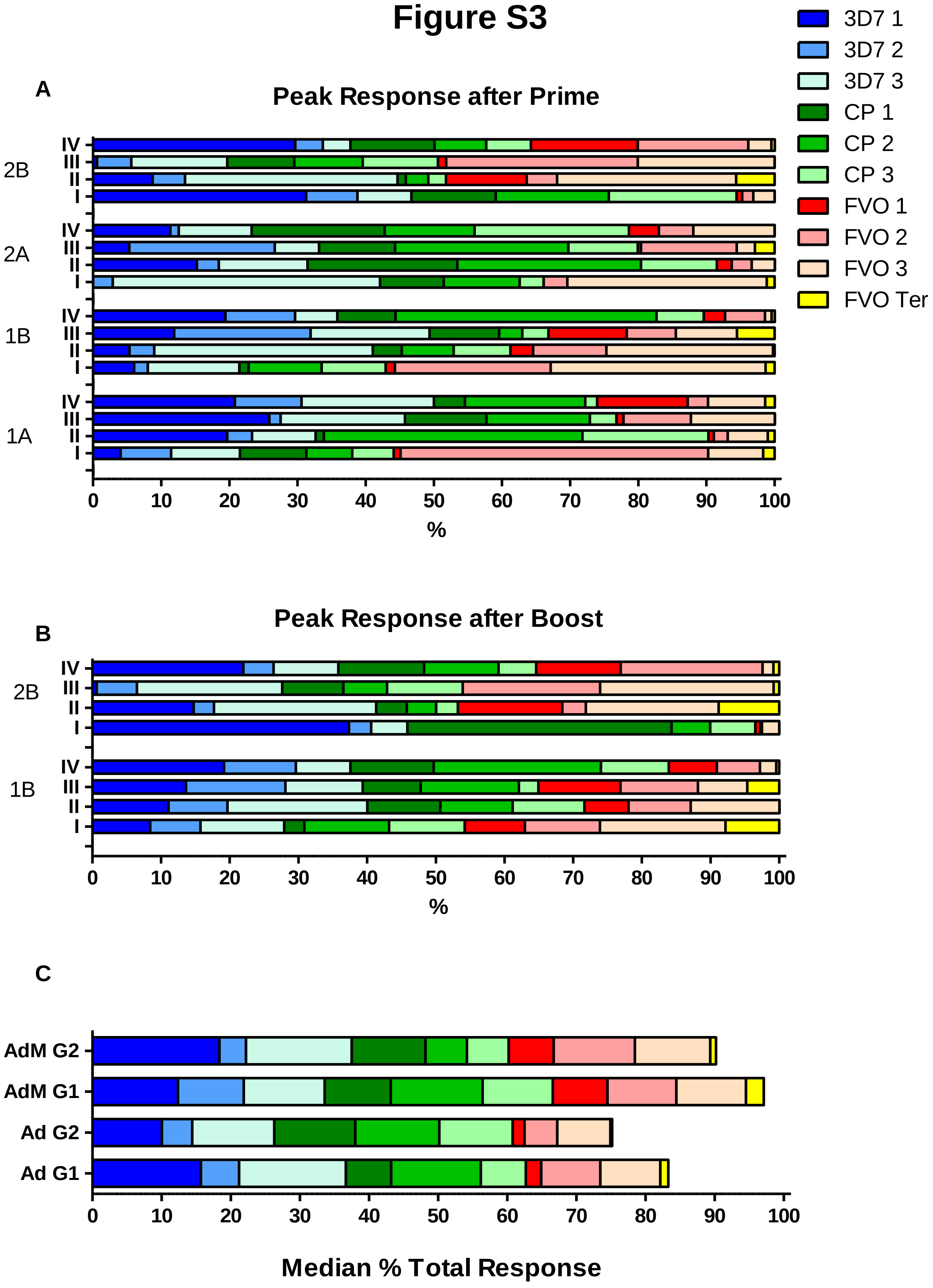
A
Peak Response after Prime
2B
2A
1B
1A
IV
III
II
I
0 10 20 30 40 50 60 70 80 90 100
%
B
Peak Response after Boost
2B
1B
IV
III
II
I
0 10 20 30 40 50 60 70 80 90 100
%
C
AdM G2
AdM G1
Ad G2
Ad G1
0 10 20 30 40 50 60 70 80 90 100
Median % Total Response
3D7 1
3D7 2
3D7 3
CP 1
CP 2
CP 3
FVO 1
FVO 2
FVO 3
FVO Ter

Figure S3. Breakdown of e*x-vivo* IFN-γ ELISPOT data according to % response. (A) Data show the % of the total response to each peptide pool within the AMA1 insert for each volunteer at day 14 after ChAd63 immunization. **(B)** Data show the % of the total response to each peptide pool within the AMA1 insert for each volunteer at the peak of the response (day 63) after ChAd63-MVA immunization. **(C)** The median response to peptide pool within the AMA1 insert according to group (G1 or G2) and immunization regime (Ad = ChAd63, AdM = ChAd63-MVA) at the peak time-point (day 14 after ChAd63 prime and day 63 after ChAd63-MVA prime-boost). MVA dosage explained in Figure 1. Volunteer I = 2B (i), Volunteers II-IV = 2B (ii).
