# Supplementary material for: Phase Ia Clinical Evaluation of the Safety and Immunogenicity of the Plasmodium falciparum Blood-Stage Antigen AMA1 in ChAd63 and MVA Vaccine Vectors
Source: PLoS One. 2012 Feb 21;7(2):e31208. doi: 10.1371/journal.pone.0031208 (PMC3283618; doi:10.1371/journal.pone.0031208)
Supplement: Figure S4 — T cell responses to tPA sequence, Vaccine and Native pools. (A) Data show ex-vivo IFN-γ ELISPOT responses to the tPA peptide pool for all volunteers at each time-point during the trial (n = 16 for d0–d56; n = 8 for d63–d140). Individual data points are shown as well as the median. Nominal cut-off for a significant response marked as 50 SFU/million PBMC. (B) Data show ex-vivo IFN-γ ELISPOT responses to peptide pools for Vaccine and Native sequences for all volunteers at the peak time-point after the prime (n = 16) and peak time-point after the boost (n = 8). Individual data points and median are shown. The responses shown represent data measured using a single pool of peptides (n = 21 peptides in each). In each graph: Group 1A (open circles); Group 1B (open squares); Group 2A (closed circles); Group 2B (closed squares). (PDF) [file pone.0031208.s005.pdf]

Figure S4

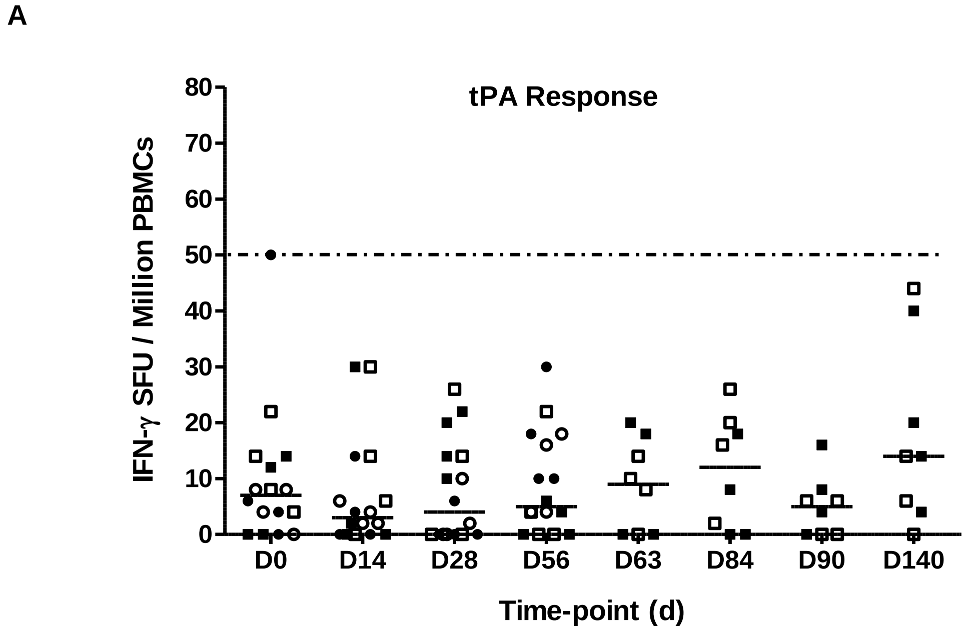

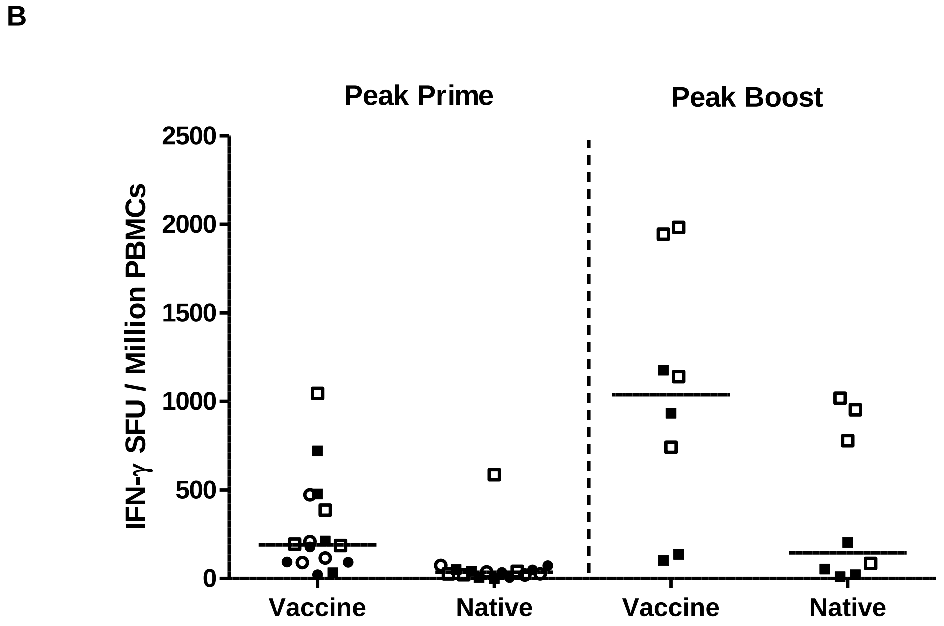

Figure S4: T cell responses to tPA sequence, Vaccine and Native pools. (A) Data show *ex-vivo* IFN-γ ELISPOT responses to the tPA peptide pool for all volunteers at each time-point during the trial (n = 16 for d0-d56; n = 8 for d63-d140). Individual data points are shown as well as the median. Nominal

cut-off for a significant response marked as 50 SFU/million PBMC. **(B)** Data show *ex-vivo* IFN-γ ELISPOT responses to peptide pools for Vaccine and Native sequences for all volunteers at the peak time-point after the prime (*n* = 16) and peak time-point after the boost (*n* = 8). Individual data points and median are shown. The responses shown represent data measured using a single pool of peptides (*n* = 21 peptides in each). In each graph: Group 1A (open circles); Group 1B (open squares); Group 2A (closed circles); Group 2B (closed squares).
