# Supplementary material for: Phase Ia Clinical Evaluation of the Safety and Immunogenicity of the Plasmodium falciparum Blood-Stage Antigen AMA1 in ChAd63 and MVA Vaccine Vectors
Source: PLoS One. 2012 Feb 21;7(2):e31208. doi: 10.1371/journal.pone.0031208 (PMC3283618; doi:10.1371/journal.pone.0031208)
Supplement: Figure S5 — T cell multi-functionality following ChAd63-MVA AMA1 immunization. The multi-functionality of the CD3+ T cell responses was assessed by polychromatic flow cytometry and ICS. Frozen PBMCs from day 84 were re-stimulated with a pool of AMA1 peptides and cells were stained as described. Gating strategy and representative plots are shown in Figure S6. The multi-functional compositions of the T cell responses following ChAd63-MVA immunization are shown for (A) CD4+ and CD8+ T cells in Group 1B following AMA1 peptide re-stimulation, and (B) CD4+ and CD8+ T cells in Group 2B following AMA1 peptide re-stimulation. Responses are grouped and colour-coded according to the CD4+ and CD8+ subsets, and the number of functions detected for each T cell population. Individual data points and median percentage of the parent CD4+ or CD8+ response (open bars) are shown for each of the functional populations indicated on the x-axis. The pie charts summarize the fractions of AMA1-specific CD4+ or CD8+ T cells that are positive for a given number of functions. (PDF) [file pone.0031208.s006.pdf]

Figure S5

A

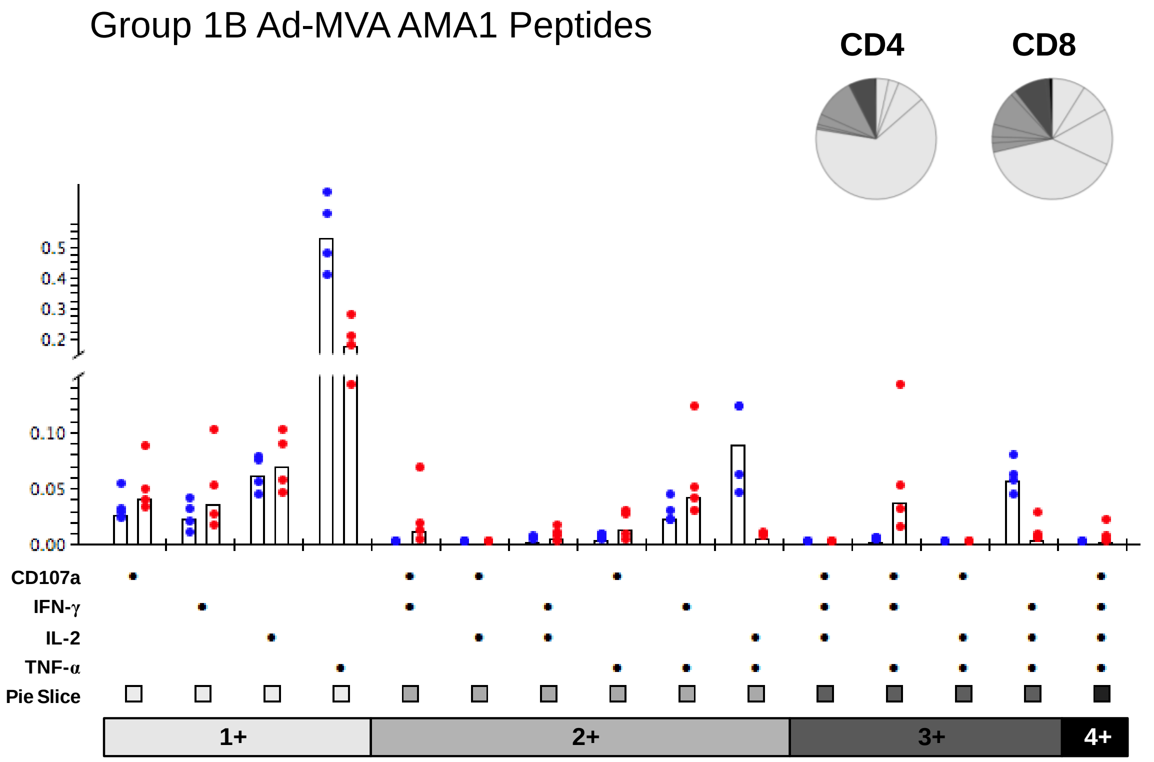
Group 1B Ad-MVA AMA1 Peptides
CD4
CD8
0.5
0.4
0.3
0.2
0.10
0.05
0.00
CD107a
IFN-γ
IL-2
TNF-α
Pie Slice
1+
2+
3+
4+

B

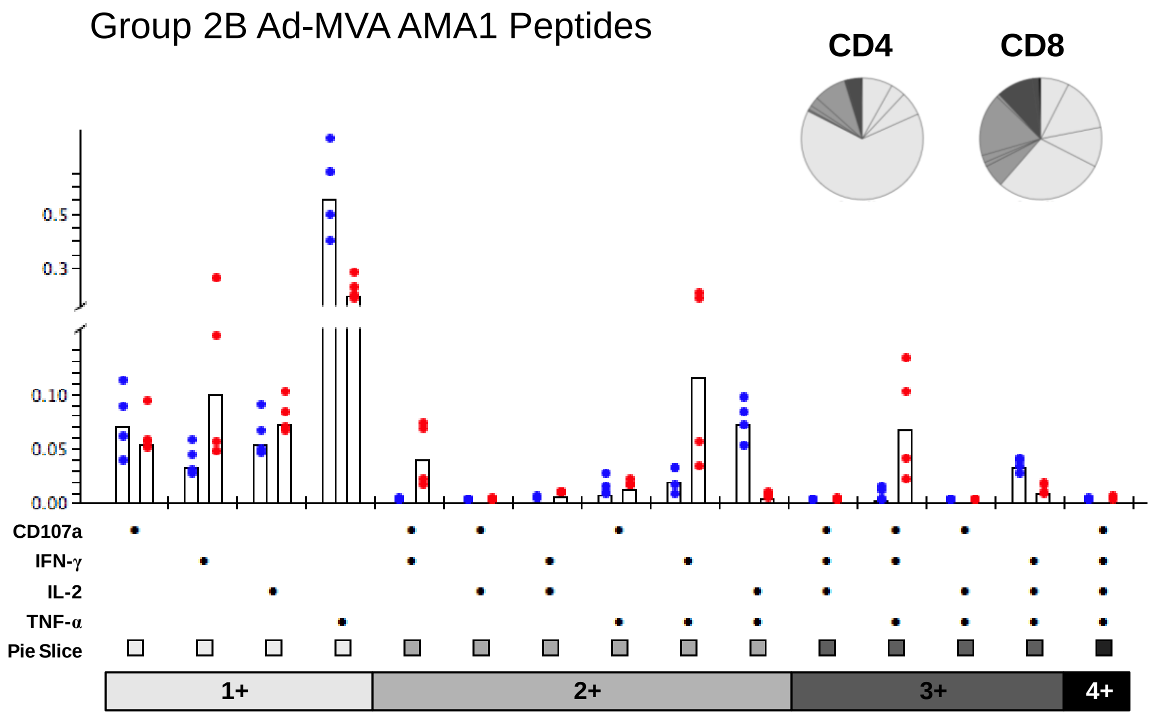
Group 2B Ad-MVA AMA1 Peptides
CD4
CD8
0.5
0.3
0.10
0.05
0.00
CD107a
IFN-γ
IL-2
TNF-α
Pie Slice
1+
2+
3+
4+

Figure S5. T cell multi-functionality following ChAd63-MVA AMA1 immunization. The multi-functionality of the $CD3^+$ T cell responses was assessed by polychromatic flow cytometry and ICS. Frozen PBMCs from day 84 were re-stimulated with a pool of AMA1 peptides and cells were stained as described. Gating strategy and representative plots are shown in Figure S6. The multi-functional compositions of the T cell responses following ChAd63-MVA immunization are shown for **(A)** $CD4^+$ and $CD8^+$ T cells in Group 1B following AMA1 peptide re-stimulation, and **(B)** $CD4^+$ and $CD8^+$ T cells in Group 2B following AMA1 peptide re-stimulation. Responses are grouped and colour-coded according to the $CD4^+$ and $CD8^+$ subsets, and the number of functions detected for each T cell population. Individual data points and median percentage of the parent $CD4^+$ or $CD8^+$ response (open bars) are shown for each of the functional populations indicated on the *x*-axis. The pie charts summarize the fractions of AMA1-specific $CD4^+$ or $CD8^+$ T cells that are positive for a given number of functions.
