# Supplementary material for: Phase Ia Clinical Evaluation of the Safety and Immunogenicity of the Plasmodium falciparum Blood-Stage Antigen AMA1 in ChAd63 and MVA Vaccine Vectors
Source: PLoS One. 2012 Feb 21;7(2):e31208. doi: 10.1371/journal.pone.0031208 (PMC3283618; doi:10.1371/journal.pone.0031208)
Supplement: Figure S6 — Gating strategy for analysis of AMA1-specific T cell responses. Representative flow cytometry plots are shown for the analysis of AMA1-specific T cell responses from volunteers immunized with ChAd63-MVA AMA1. (A) Initial gating used (from top left to bottom right) forward scatter area (FSC-A) versus forward scatter height (FSC-H) to remove doublet events and select singlet cells; then following this small lymphocytes were gated using FSC-A versus side scatter area (SSC-A); then live CD14− CD20− CD3+ cells were selected; then CD4 versus CD8 was used to select the total CD4+ CD8− cell population and vice versa for the CD8+ CD4− population. Cytokine (IFN-γ, IL-2 and TNFα) and CD107a gating using bivariate plots is shown for (B) CD4+ cells and (C) CD8+ cells. (B) Representative plots for un-stimulated (UNS), AMA1 peptide stimulated (AMA1), SEB, uRBC and iRBC stimulated samples are shown. IFN-γ (top row), IL-2 (second row), TNFα (third row) and CD107a (bottom row) for the CD8− CD4+ T cell population were analyzed using bivariate plots. Percentages refer to the % of CD8− CD4+ cells that express the specific cytokine or marker. Background responses in UNS or uRBC control cells were subtracted from the AMA1 and iRBC response respectively during the analysis. (C) Same analysis as in (B), except for the CD4− CD8+ T cell population. (PDF) [file pone.0031208.s007.pdf]

Figure S6

A

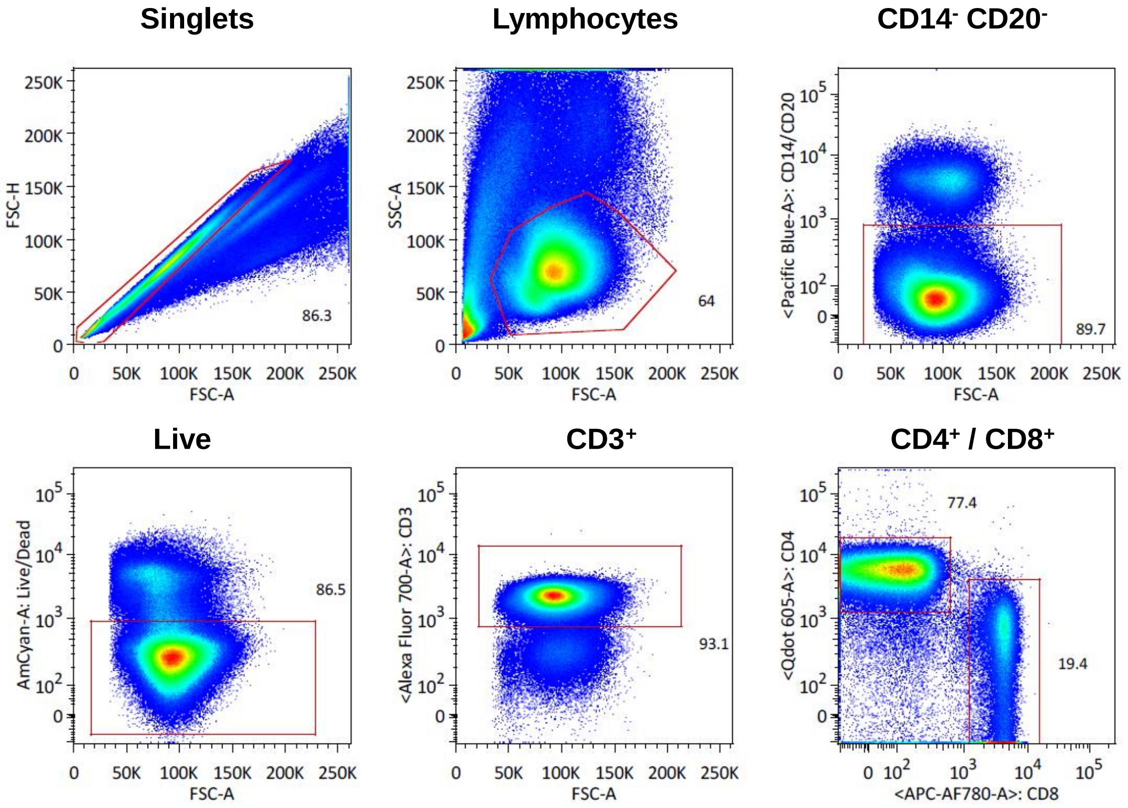

Singlets
Lymphocytes
CD14⁻ CD20⁻
Live
CD3⁺
CD4⁺ / CD8⁺
FSC-H
SSC-A
<Pacific Blue-A>: CD14/CD20
AmCyan-A: Live/Dead
<Alexa Fluor 700-A>: CD3
<Qdot 605-A>: CD4
FSC-A
<APC-AF780-A>: CD8
86.3
64
89.7
86.5
93.1
77.4
19.4

B

CD4$^+$ cells

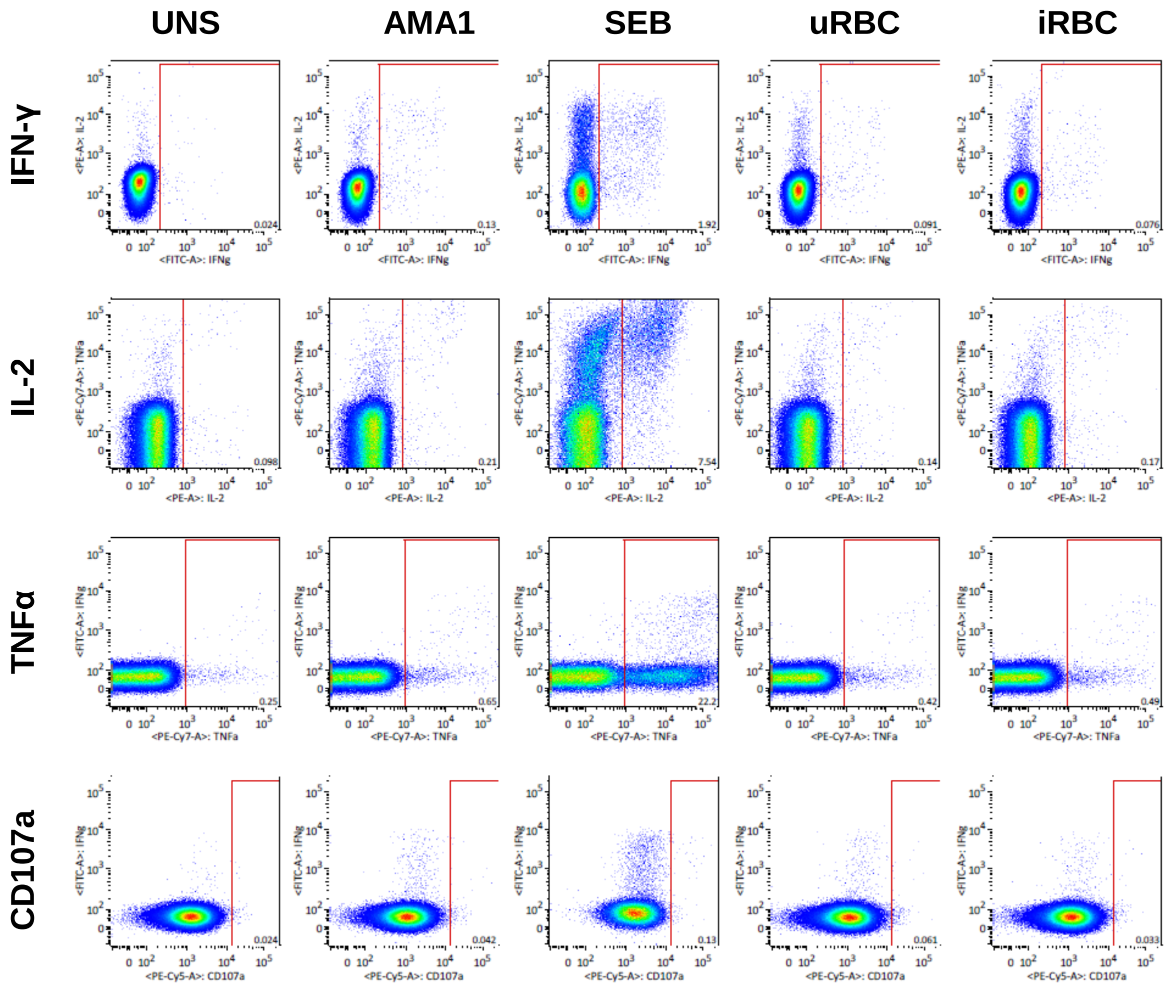
UNS
AMA1
SEB
uRBC
iRBC
IFN-γ
IL-2
TNFα
CD107a
<FITC-A>: IFNg
<PE-A>: IL-2
<PE-Cy7-A>: TNFa
<PE-Cy5-A>: CD107a
0.024
0.13
1.92
0.091
0.076
0.098
0.21
7.54
0.14
0.17
0.25
0.65
22.2
0.42
0.49
0.024
0.042
0.13
0.061
0.033

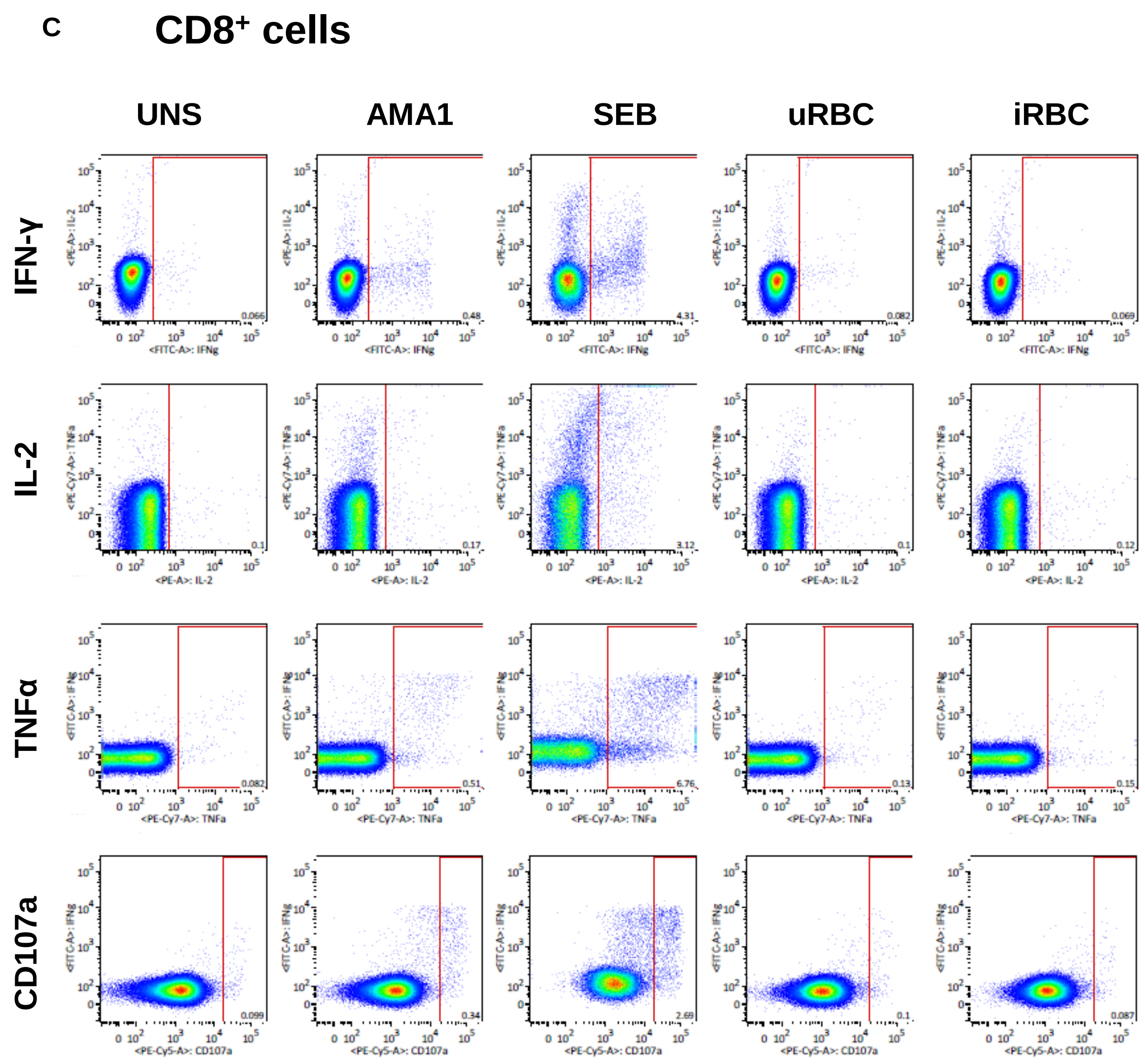

Figure S6. Gating strategy for analysis of AMA1-specific T cell responses. Representative flow cytometry plots are shown for the analysis of AMA1-specific T cell responses from volunteers immunized with ChAd63-MVA AMA1. **(A)** Initial gating used (from top left to bottom right) forward scatter area (FSC-A) versus forward scatter height (FSC-H) to remove doublet events and select singlet cells; then following this small lymphocytes were gated using FSC-A versus side scatter area (SSC-A); then live $CD14^-$ $CD20^-$ $CD3^+$ cells were selected; then CD4 versus CD8 was used to select the total $CD4^+$ $CD8^-$ cell population and vice versa for the $CD8^+$ $CD4^-$ population. Cytokine (IFN-γ, IL-2 and TNFα) and CD107a gating using bivariate plots is shown for **(B)** $CD4^+$ cells and **(C)** $CD8^+$ cells. **(B)** Representative plots for un-stimulated (UNS), AMA1 peptide stimulated (AMA1), SEB, uRBC and iRBC

stimulated samples are shown. IFN-γ (top row), IL-2 (second row), TNFα (third row) and CD107a (bottom row) for the $CD8^-$ $CD4^+$ T cell population were analyzed using bivariate plots. Percentages refer to the % of $CD8^-$ $CD4^+$ cells that express the specific cytokine or marker. Background responses in UNS or uRBC control cells were subtracted from the AMA1 and iRBC response respectively during the analysis. **(C)** Same analysis as in **(B)**, except for the $CD4^-$ $CD8^+$ T cell population.
