# Supplementary material for: Phase Ia Clinical Evaluation of the Safety and Immunogenicity of the Plasmodium falciparum Blood-Stage Antigen AMA1 in ChAd63 and MVA Vaccine Vectors
Source: PLoS One. 2012 Feb 21;7(2):e31208. doi: 10.1371/journal.pone.0031208 (PMC3283618; doi:10.1371/journal.pone.0031208)
Supplement: Figure S7 — Individual IgG ELISA data. Total IgG ELISA responses against 3D7 PfAMA1 as measured in the serum over time following immunization in (A) Group 1A (n = 4), (B) Group 1B (n = 4), (C) Group 2A (n = 4), and (D) Group 2B (n = 4). MVA dosage explained in Figure 1. Volunteer I = 2B (i), Volunteers II–IV = 2B (ii). (PDF) [file pone.0031208.s008.pdf]

Figure S7

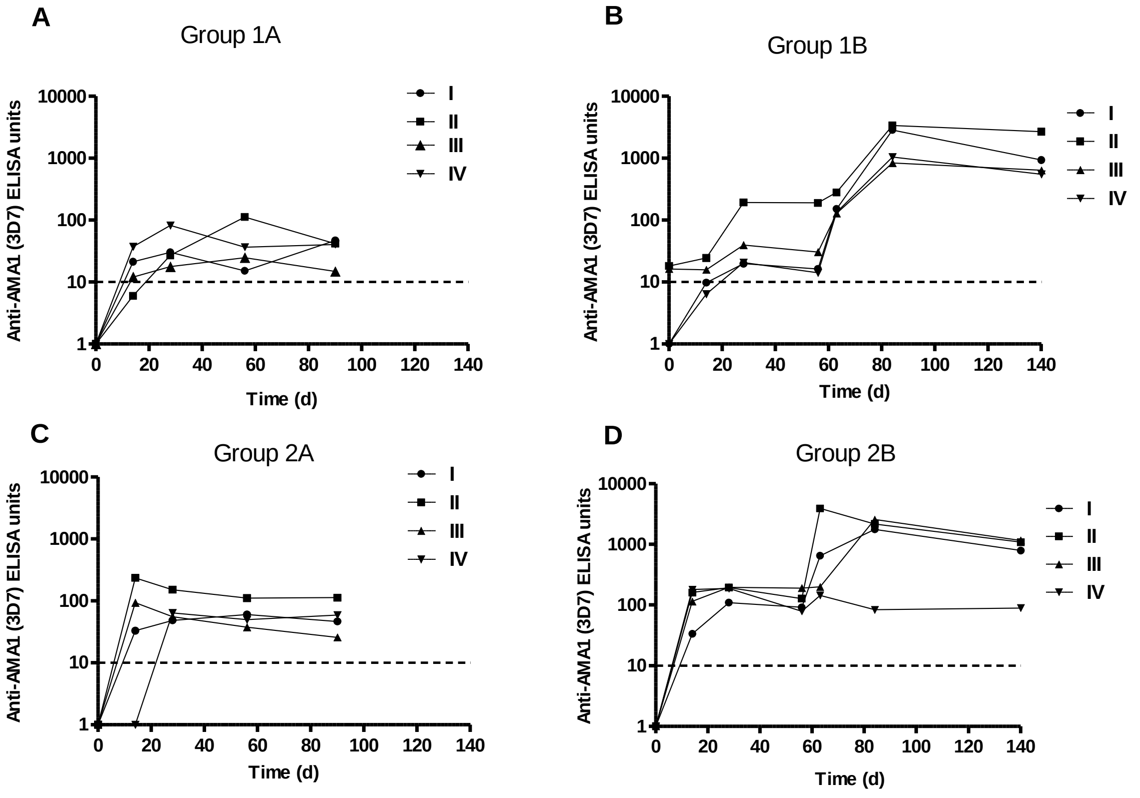

Figure S7. Individual IgG ELISA data. Total IgG ELISA responses against 3D7 PfAMA1 as measured in the serum over time following immunization in **(A)** Group 1A (*n* = 4), **(B)** Group 1B (*n* = 4), **(C)** Group 2A (*n* = 4), and **(D)** Group 2B (*n* = 4). MVA dosage explained in Figure 1. Volunteer I = 2B (i), Volunteers II-IV = 2B (ii).
