# Supplementary material for: Phase Ia Clinical Evaluation of the Safety and Immunogenicity of the Plasmodium falciparum Blood-Stage Antigen AMA1 in ChAd63 and MVA Vaccine Vectors
Source: PLoS One. 2012 Feb 21;7(2):e31208. doi: 10.1371/journal.pone.0031208 (PMC3283618; doi:10.1371/journal.pone.0031208)
Supplement: Figure S8 — Relationship between total IgG ELISA titers and % GIA. (A) Spearman's correlation of serum IgG ELISA titers against AMA1 for the 3D7 versus FVO alleles at day 84, n = 8. (B) Relationship between 3D7 strain % GIA and FVO strain % GIA using purified IgG at 10 mg/mL. (PDF) [file pone.0031208.s009.pdf]

Figure S8

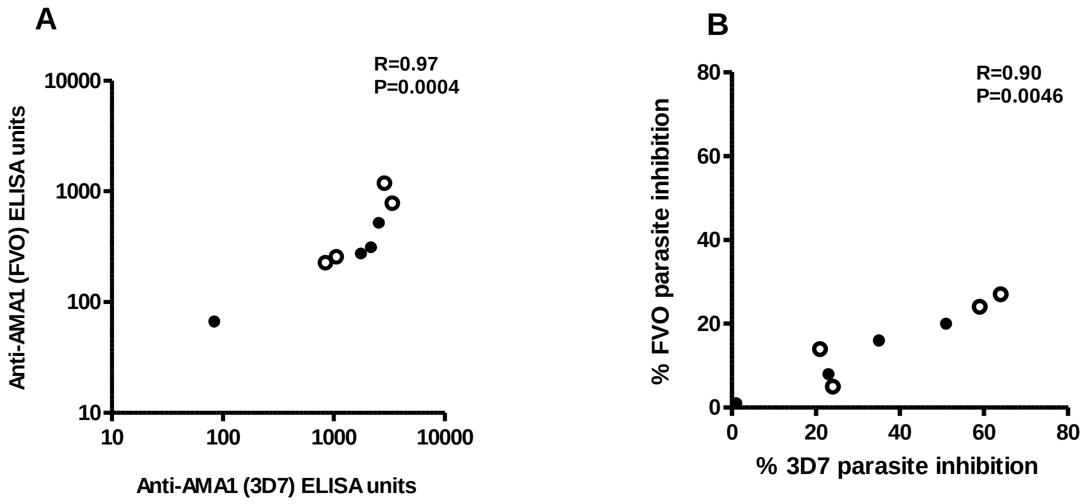

Figure S8. Relationship between total IgG ELISA titers and % GIA. (A) Spearman's correlation of serum IgG ELISA titers against AMA1 for the 3D7 versus FVO alleles at day 84, *n* = 8. **(B)** Relationship between 3D7 strain % GIA and FVO strain % GIA using purified IgG at 10mg/mL.
