# Supplementary material for: Phase Ia Clinical Evaluation of the Safety and Immunogenicity of the Plasmodium falciparum Blood-Stage Antigen AMA1 in ChAd63 and MVA Vaccine Vectors
Source: PLoS One. 2012 Feb 21;7(2):e31208. doi: 10.1371/journal.pone.0031208 (PMC3283618; doi:10.1371/journal.pone.0031208)
Supplement: Figure S9 — Baseline NAb responses against ChAd63. NAb titers against ChAd63 were assayed in the pre-immunization (day 0) serum of all volunteers. (A) Individual and median responses in Groups 1 and 2 are shown. The dotted lines indicate the level at which responses were classed as negative (titer<1∶18) and high (titers>1∶200). There was no significant difference between the volunteers enrolled into Group 1 or Group 2 (n = 8 per group, P = 0.18, Mann Whitney test). There were no significant correlations in either (B) Group 1 or (C) Group 2 between these low level pre-existing NAb titers and ChAd63 AMA1 immunogenicity as assessed by peak total summed AMA1 ELISPOT response at day 14 (blue) or anti-AMA1 (3D7) total IgG ELISA at day 28 (red). These were selected as the peak of the priming immune responses following ChAd63 AMA1 immunization. rs = 0.36, P = 0.39 (ELISA) or rs = −0.53, P = 0.20 (ELISPOT) in Group 1; and rs = 0.32, P = 0.43 (ELISA) or rs = −0.02, P = 0.98 (ELISPOT) in Group 2. (PDF) [file pone.0031208.s010.pdf]

Figure S9

A

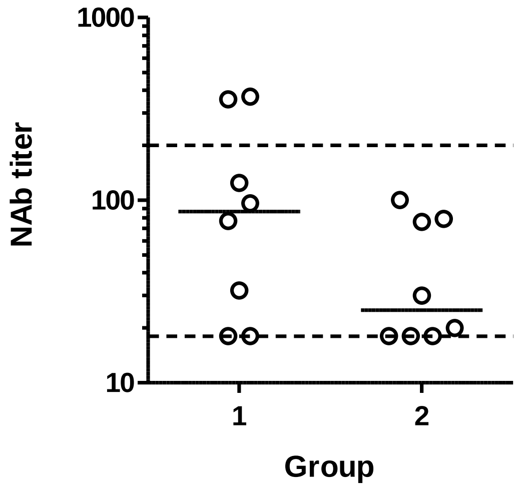

B

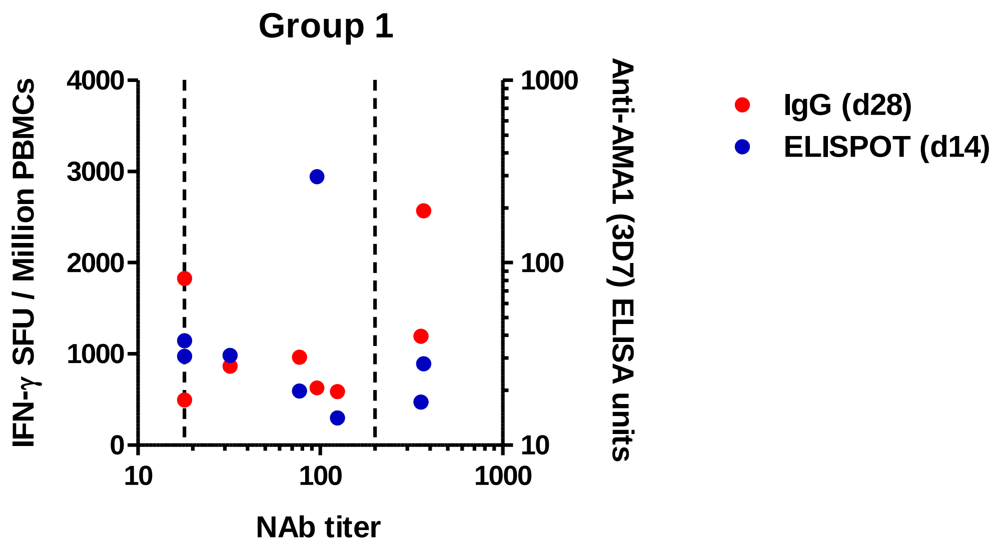

C

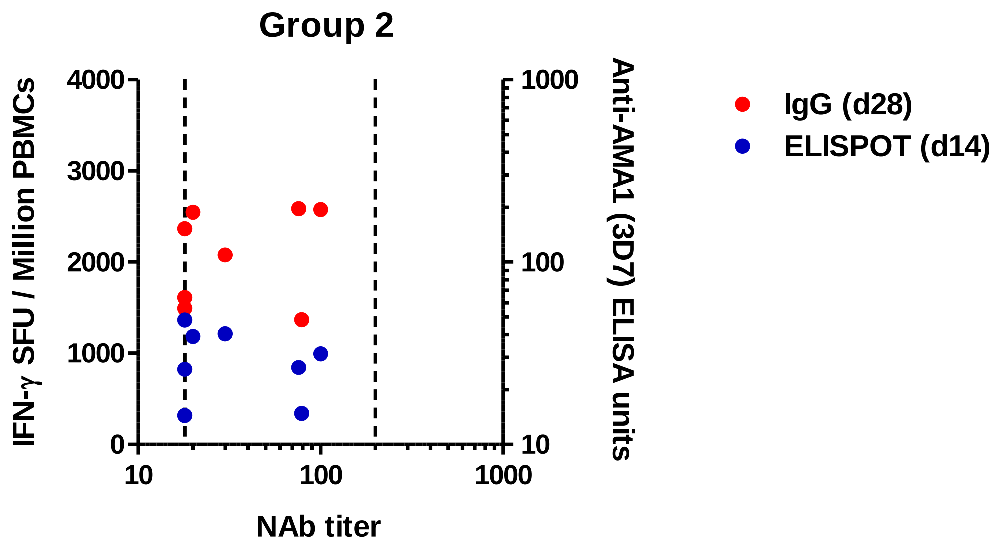

Figure S9. Baseline NAb responses against ChAd63. NAb titers against ChAd63 were assayed in the pre-immunization (day 0) serum of all volunteers. **(A)** Individual and median responses in Groups 1 and 2 are shown. The dotted lines indicate the level at which responses were classed as negative (titer <1:18) and high (titers >1:200). There was no significant difference between the volunteers enrolled into Group 1 or Group 2 (*n* = 8 per group, *P* = 0.18, Mann Whitney test). There were no significant correlations in either **(B)** Group 1 or **(C)** Group 2 between these low level pre-existing NAb titers and ChAd63 AMA1 immunogenicity as assessed by peak total summed AMA1 ELISPOT response at day 14 (blue) or anti-AMA1 (3D7) total IgG ELISA at day 28 (red). These were selected as the peak of the priming immune responses following ChAd63 AMA1 immunization. r_s = 0.36, *P* = 0.39 (ELISA) or r_s = -0.53, *P* = 0.20 (ELISPOT) in Group 1; and r_s = 0.32, *P* = 0.43 (ELISA) or r_s = -0.02, *P* = 0.98 (ELISPOT) in Group 2.
