# Supplementary material for: Phase Ia Clinical Evaluation of the Safety and Immunogenicity of the Plasmodium falciparum Blood-Stage Antigen AMA1 in ChAd63 and MVA Vaccine Vectors
Source: PLoS One. 2012 Feb 21;7(2):e31208. doi: 10.1371/journal.pone.0031208 (PMC3283618; doi:10.1371/journal.pone.0031208)
Supplement: Protocol S1 — Trial Protocol. (PDF) [file pone.0031208.s011.pdf]

UNIVERSITY OF OXFORD

Study Reference: VAC 036

A phase I study to assess the safety and immunogenicity of new malaria vaccine candidates AdCh63 AMA1 alone and with MVA AMA1

Sponsor: University of Oxford

Chief Investigator: Professor Adrian V.S. Hill

Modification History

Version	Date	Author(s)
1	06/08/07	Geraldine O’Hara, Simon Draper, Adrian Hill.
2	20/09/07	Geraldine O’Hara.
3	28/02/09	Geraldine O’Hara, Simon Draper, Adrian Hill.
4	14/05/09	Christopher Duncan
5	06/10/2009	Susanne Sheehy, Christopher Duncan, Adrian Hill
6	16/12/2009	Susanne Sheehy, Alison Lawrie
6.1	23/02/2010	Susanne Sheehy
7.0	26/02/2010	Susanne Sheehy, Alison Lawrie.
8.0	02/06/2010	Susanne Sheehy, Adrian Hill

Changes since version 7.0

Section	Modification
1.0 Synopsis	Updated to reflect change in dose of MVA AMA1
3.6 Simian Adenoviruses	Update Safety and Immunogenicity Data for AdCh63-MVA MSP1 & AdCh63-MVA AMA1
3.9 Potential Risks	Updated Potential Adverse Events for AdCh63 AMA1 & MVA AMA1
5.1 Vaccine insert, formulation, storage and accountability	Dose of MVA AMA1 changed
5.3 MVA AMA1 Dose	Dose of MVA AMA1 updated
7.2 Study Groups	Dose of MVA AMA1 updated

A phase I study to assess the safety and immunogenicity of new malaria vaccine candidates AdCh63 AMA1 alone and with MVA AMA1

Study Code: VAC 036

EudraCT Number: 2007-004567-21

Chief Investigator:	Professor Adrian V.S. Hill<br><br>Centre for Clinical Vaccinology and Tropical Medicine<br>Churchill Hospital, Old Road, Headington<br>Oxford, OX3 7LJ<br>Tel: 01865 857417<br>Fax: 01865 857471<br>Email: adrian.hill@ndm.ox.ac.uk
Principle Investigator:	Dr Tom Doherty<br>Hospital for Tropical Diseases<br>Mortimer Market<br>Capper Street<br>London<br>WC1E 6JB<br>Tel : 08451 555 000 x5409<br>Fax : 0207 3809761<br>Email : tom.doherty@uclh.nhs.uk
Centres:	Centre for Clinical Vaccinology and Tropical Medicine<br>Churchill Hospital, Old Road, Headington<br>Oxford, OX3 7LJ<br>Tel: 01865 857417<br>Fax: 01865 857471<br>(Principle Investigator at Site: Prof Adrian Hill)<br><br>University College London Clinical Research Facility<br>c/o Rayne Building<br>5 University Street<br>London<br>WC1E 6JJ<br>Tel : 020 7679 6351<br>Fax : 020 7691 2838<br>(Principle Investigator at Site : Dr Tom Doherty)
Sponsoring Institution	University of Oxford<br>Clinical Trials and Research Governance<br>Manor House, The John Radcliffe Hospital,<br>Headington, Oxford, OX3 9DZ<br>Tel: 01865 743004

	Fax: 01865 743002<br>Email: heather.house@admin.ox.ac.uk
Clinical Project Manager	Dr Alison Lawrie<br>CCVTM, Oxford<br>Tel: 01865 857382
Local Safety Committee	Dr Brian Angus<br>Centre for Clinical Vaccinology and Tropical Medicine<br>Churchill Hospital, Old Road, Headington<br>Oxford, OX3 7LJ<br>Tel: 01865 553 20289<br>Email: brian.angus@ndm.ox.ac.uk
External Monitor	Appledown Clinical Research Limited<br>Orchard's End,<br>Greenland's Lane,<br>Prestwood,<br>Gt. Missenden,<br>Bucks.<br>HP16 9QX<br>Tel/Fax: +44 (0)1494 867676

Investigator Agreement

"I have read this protocol and agree to abide by all provisions set forth therein.

I agree to comply with the principles of the International Conference on Harmonisation Tripartite Guideline on Good Clinical Practice."

--	--	------------------------
Chief Investigator	Investigator Signature	Date
Professor Adrian Hill		

Confidentiality Statement

This document contains confidential information that must not be disclosed to anyone other than the Sponsor, the Investigator Team, and members of the Independent Ethics Committee. This information cannot be used for any purpose other than the evaluation or conduct of the clinical investigation without the prior written consent of Professor Adrian Hill.

TABLE OF CONTENTS

1. SYNOPSIS 9
2. ABBREVIATIONS 10
3. BACKGROUND AND RATIONALE 11
3.1 The need for a new vaccine against malaria 11
3.2 Lifecycle of the malaria parasite 11
3.3 Rationale for a blood stage vaccine against malaria 11
3.4 AMA1 as an insert 12
3.5 Human adenovirus as a vector 14
3.6 Simian adenoviruses 15
3.7 Clinical trials with malaria vaccines 18
3.8 Study overview 38
3.9 Potential Risks 41
3.10 Known Potential Benefits 42
4. OBJECTIVES 43
4.1 Primary Objectives 43
4.2 Secondary Objectives 43
5. INVESTIGATIONAL PRODUCTS 44
5.1 Vaccine insert, formulation, storage and accountability 44
5.2 AdCh63 AMA1 dose 44
5.3 MVA AMA1 dose 44
5.4 Vaccine administration 45
5.5 Minimising environmental contamination with Genetically Modified Organisms (GMO) ... 45
6. RECRUITMENT AND WITHDRAWAL OF TRIAL VOLUNTEERS 46
6.1 Volunteers 46
6.2 Informed Consent 46
6.3 Inclusion and Exclusion Criteria 46
6.4 Withdrawal of Volunteers 49
6.5 Compliance with Dosing Regime 49
6.6 Pregnancy 49

7. TRIAL DESIGN 50
7.1 Primary and Secondary Endpoints 50
7.2 Study Groups 50
7.3 First Volunteers 50
7.4 Duration of Study 51
7.5 Definition of the Start and End of the Trial 51
8. TREATMENT OF TRIAL VOLUNTEERS 52
8.1 Study procedures 52
8.2 Study visits 53
9. ASSESSMENT OF SCIENTIFIC OBJECTIVES 57
9.1 Primary Outcome Measures 57
9.2 Secondary Outcome Measures 57
10. ASSESSMENT OF SAFETY 58
10.1 Interim Safety Review 58
10.2 Definitions 58
10.3 Causality Assessment 59
10.4 Reporting Procedures for All Adverse Events 60
10.5 63
10.6 Reporting Procedures for Serious Adverse Events 63
10.7 Procedures to be followed in the event of abnormal findings 63
10.8 Local Safety Committee 63
10.9 Safety Profile Review 64
11. STATISTICS 65
12. QUALITY CONTROL AND QUALITY ASSURANCE PROCEDURES 66
13. ETHICS 67
13.1 Declaration of Helsinki 67
13.2 ICH Guidelines for Good Clinical Practice 67
13.3 Informed Consent 67
13.4 Independent Ethics Committee (IEC) 67

13.5 Volunteer Confidentiality 67
14. DATA HANDLING AND RECORD KEEPING 68
14.1 Data Handling 68
14.2 Record Keeping 68
14.3 Source Data and Case Report Forms (CRFs) 68
14.4 Data Protection 68
15. FINANCING AND INSURANCE..69
15.1 Financing 69
15.2 Insurance 69
15.3 Compensation..69
References..70

1. SYNOPSIS

**Title**	**A phase I study to assess the safety and immunogenicity of new malaria vaccine candidates AdCh63 AMA1 alone and with MVA AMA1**
**Trial Centres**	Centre for Clinical Vaccinology and Tropical Medicine (CCVTM)<br>Old Road, Headington, Oxford, OX3 7LJ, UK<br><br>University College London Clinical Research Facility (UCL CRF)<br>C/O Rayne Building, 5 Universiry Street, London, WC1E 6JJ
**Trial Identifier**	VAC 036
**Clinical Phase**	I
**Design**	Open label observational study
**Population**	Healthy adults aged 18 – 50
**Sample Size**	**Group 1**<br>**Subgroup A, (1A)**: 4 volunteers; 1 dose of AdCh63 AMA1 5 x 10^9 vp intramuscularly<br>**Subgroup B (1B):** 4 volunteers,1 dose of AdCh63 AMA1 5 x 10^9 vp intramuscularly and 1 dose MVA AMA1 2.5 x 10^8 pfu 8 weeks later intramuscularly<br><br>**Group 2**<br>**Subgroup A (2A)**: 4 volunteers; 1 dose of AdCh63 AMA1 5 x 10^{10} vp intramuscularly<br>**Subgroup B** (**2B) :** 4 volunteers,1 dose of AdCh63 AMA1 5 x 10^{10} vp intramuscularly and 1 dose MVA AMA1 1.25 x 10^8 pfu 8 weeks later intramuscularly<br><br>Total: 16 volunteers
**Follow-up duration**	Approximately 3 - 6 months (This is an estimate and may vary in accordance with the specified time windows for each attendance.)
**Planned Trial Period**	12 months
**Primary Objective**	To assess the safety in healthy volunteers of two new candidate malaria vaccines, AdCh63 AMA1 administered alone, and with MVA AMA1, in a prime-boost regime.
**Secondary Objective**	To assess the humoral and cellular immune responses generated by AdCh63 AMA1, when administered to healthy volunteers alone and with MVA AMA1.
**Investigational Products**	1. AdCh63 AMA1<br>2. MVA AMA1
**Form**	Liquid
**Dose**	AdCh63 AMA1: 5 x 10^9 vp, 5 x 10^{10} vp<br>MVA AMA1: 2.5 x 10^8 pfu<br>MVA AMA1: 1.25 x 10^8 pfu
**Route**	Intramuscular injection in the deltoid region of the arm

2. ABBREVIATIONS

**AdCh63**	Chimpanzee adenovirus 63
**AdCh63 AMA1**	Recombinant chimpanzee adenovirus 63 encoding apical membrane antigen 1
**AdHu**	Human adenovirus
**AdHu5**	Human adenovirus serotype 5
**AE**	Adverse event
**AMA1**	Apical membrane antigen 1
**CCVTM**	Centre for Clinical Vaccinology and Tropical Medicine
**CBF**	Clinical Bio manufacturing Facility
**CRF**	Case Report Form
**CSO / CS**	Circumsporozoite protein
**ELISPOT**	Enzyme-linked immunospot
**FBC**	Full blood count
**GCP**	Good Clinical Practice
**GIA**	Growth Inhibition Assays
**GMO**	Genetically modified organism
**GTAC**	Gene Therapy Advisory Committee
**HBsAg**	Hepatitis B Surface Antigen
**HCG**	Human Chorionic Gonadotrophin
**HCV**	Hepatitis C virus
**HIV**	Human immunodeficiency virus
**HLA**	Human leukocyte antigen
**IDT**	Impfstoffwerk Dessau-Tornau
**IEC**	Independent Ethics Committee
**LSM**	Local safety monitor
**MHRA**	Medicines and Healthcare products Regulatory Agency
**MSP**	Merozoite Surface Protein
**MVA**	Modified Vaccinia Virus Ankara
**NHS**	National Health Service
**RESA**	Ring Erythrocyte surface antigen
**SAE**	Serious adverse event
**SOP**	Standard Operating Procedure
**SUSAR**	Suspected unexpected serious adverse reaction
**µg**	microgram
**vp**	viral particle

3. BACKGROUND AND RATIONALE

3.1 The need for a new vaccine against malaria

Malaria is the pre-eminent tropical parasitic disease and one of the top three killers among communicable diseases. The numbers are remarkable: there are 300 to 500 million clinical cases every year, about one million deaths, mostly of children, are attributable to this disease [1]. Every 40 seconds a child dies of malaria, resulting in a daily loss of more than 2,000 young lives worldwide [2]. Malaria also remains a hazard for travellers visiting malaria-endemic regions. The development of resistance in Anopheles mosquitoes to certain insecticides and resistance of parasites to chemotherapeutic agents has led to an increasing global burden and an urgent need for a new, effective intervention [3]. A method of immunoprophylaxis, such as a malaria vaccine, is an exciting and promising potential approach. To provide a coordinated global approach to fighting malaria, the Roll Back Malaria (RBM) Partnership was launched in 1998 by the World Health Organization (WHO), the United Nations Children's Fund (UNICEF), the United Nations Development Programme (UNDP) and the World Bank. A major goal of the RBM Partnership is to support the development of a vaccine against malaria.

3.2 Lifecycle of the malaria parasite

In order to understand the development of a vaccine against malaria, it is helpful to review the lifecycle of the malaria parasite. Malaria parasites have a complex life cycle. Infected mosquitoes transmit the malaria sporozoites in their saliva to humans while taking a blood meal. The sporozoites travel from the skin through the blood stream and invade liver cells (hepatocytes), where they mature into merozoites. After 6 to 7 days, the infected hepatocytes rupture, releasing a large number of merozoites into the bloodstream. These merozoites then invade red blood cells, (erythrocytes), where they multiply and, after two days, cause the erythrocyte to rupture releasing progeny merozoites that invade new erythrocytes to perpetuate the erythrocytic cycle. This is the blood stage of malaria. Infection by sporozoites and their liver-stage development are clinically silent stages of malaria. It is the subsequent blood-stage infection that can lead to the rapid onset of symptoms and complications associated with the disease. A small percentage of the merozoites do not multiply after invading erythrocytes, but instead differentiate into sexual forms (gametocytes). When ingested by a mosquito, male and female sexual forms can unite, creating a zygote. The zygote matures and releases sporozoites, which migrate to the mosquito's salivary glands, thus completing the lifecycle. Vaccines against every stage of malaria have been considered.

3.3 Rationale for a blood stage vaccine against malaria

Immunity to malaria develops naturally over time following frequent exposure. No one overriding correlate of protection has been described, however, the cumulative acquisition of protective antibodies against blood-stage antigens is well documented and arguably essential for natural

immunity [4]. The immunity that develops confers protection against severe disease such as cerebral malaria, hypoglycaemia, metabolic acidosis and renal failure. However this immunity is dependent on repeated boosting by regular infection and is therefore present in adults and not children who remain at risk of severe disease from malarial infection. A blood stage vaccine against malaria is an attractive proposition as it mimics natural immunity and if partially effective will lead to a reduction in disease severity whilst still allowing natural immunity to develop [4].

There are several lines of evidence supporting the feasibility of a blood stage vaccine: induction of protective immunity with defined antigens in animal models [5, 6]; in experimental models, the ability of adoptively transferred B cells from immune donors into B cell-deficient mice to clear parasites [6]; the age-related acquisition of immunity against severe clinical malaria in endemic regions [7, 8]; and perhaps most importantly the ability of passively transferred antibodies from immune adults to protect against natural and challenge infections with *P. falciparum* [9-11].

Natural immunity to malaria targets multiple blood stage antigens and is slowly acquired. PfEMP1 is a potential target antigen but is highly polymorphic, and immunity is variant specific [7]. Field studies have shown that more conserved merozoite surface antigens such as MSP-1 and AMA1 are also targets of naturally occurring protective blood stage immunity.

To date blood stage malaria vaccine development has focussed upon recombinant protein-in-adjuvant formulations, mainly targeting antigens involved in erythrocyte invasion. Preclinical work has shown it is possible to induce protective antibody responses, however phase 1 clinical trials have been disappointing (see section 3.7). Our previous strategy at Oxford has been to target pre-erythrocytic malaria using heterologous prime boost strategies with viral vectors which stimulate cell mediated immunity. Several studies have suggested that generating cellular immunity in conjunction with antibody responses may increase the efficacy of blood stage malaria vaccines [12]. In the *P. yoelii* blood stage murine model, recombinant AdHu5-MVA MSP-1 prime boost regime can induce complete protection to blood stage challenge and partial protection against liver stage disease [13, 14]. Vaccine induced blood stage immunity correlates with MSP-1 specific antibodies, whilst MSP-1 specific T cell responses protect against the liver stage parasite. Similar viral vectored vaccines targeting *P. falciparum* MSP-1 have shown efficacy in a standardised growth inhibition assay (GIA), comparable to any protein adjuvant vaccine, (A. Goodman et al, unpublished).

3.4 AMA1 as an insert

Apical membrane antigen -1, (AMA1), is a type I integral membrane protein with a large N-terminal cysteine rich ectodomain followed by a single transmembrane domain and a short C-terminal cytoplasmic tail. The N-terminus has 16 cysteine residues forming 8 disulphide bonds which are

conserved amongst most species. The ectodomain is subdivided into three disulphide bridged domains and the erythrocyte binding activity is associated with domains I and II.

AMA1 is produced by mature *P. falciparum* schizonts in infected erythrocytes [15, 16], and localizes in the microneme, an apical secretory organelle of the merozoite involved in red cell invasion [16]. The protein is subsequently exported to the merozoite surface at around the time of rupture of the schizont-infected erythrocyte [17]. AMA1 does not enter the erythrocyte during invasion, but remains at the invasion interface and in the areas of the merozoite that have not yet entered the erythrocyte [17]. Although its exact function remains undetermined, these observations suggest that AMA1 performs a role during merozoite invasion of erythrocytes.

AMA1 has become a leading candidate vaccine antigen. This is based on several facts, most notably that in several field studies an association was found between antibodies to the ectodomain of AMA1 and protection against clinical malaria [18]. Also, in the Gambia, presence of antibodies to AMA1 and MSP-1_{19} has been shown to enhance clearance of chloroquine resistant parasites *in vivo* [19].

It has been possible with suitable adjuvantation to induce protective antibody responses with this recombinant antigen in several simian and murine studies [20-22]. Mouse antibodies induced by an AMA1 peptide show growth inhibitory activity [23]. Several human clinical trials have been carried out with recombinant AMA1 and various adjuvants, and have generated antibodies with low to moderate levels of activity in growth inhibition assays (see section 3.7).

Viral-vectored vaccines for AMA1 have been shown to be immunogenic in mouse models [23, 24]. In the macaque based *P. knowlesi* model the US Navy group have shown significant efficacy using a combination of DNA and NYVAC vectored vaccines. This has been attributed to the use of AMA1 with MSP-1_{42} [25]. Similar pre clinical work using the blood stage antigen MSP-1 as an insert in an adenovirus prime – MVA boost regime has shown the ability to induce high titre protective antibody responses in the murine *P. yoelii* model. In addition, using this vaccination regime we can demonstrate multi-stage immunity, with MSP-1_{42} specific T cells providing clearance of late liver stage parasites [13]. AMA1 should also provide such multi stage immunity, given its expression by the sporozoite [26] and also presumably by merozoites during development of late liver stage parasites.

AMA1 polymorphism presents a potential issue for the development of a widely effective vaccine. Most allelic diversity is reported in domains I and III in response to immune selection pressure [27]. Studies of naturally exposed individuals have shown both strain-specific and cross-reactive antibody responses, but only responses against the entire ectodomain, rather than individual epitopes correlate with protection [18]. In this trial we attempt to address the issue of polymorphism of AMA1, at least in part, by including two quite divergent alleles of AMA1 (3D7 and FVO) in tandem as the vaccine insert in both AdCh63 and MVA.

3.5 Human adenovirus as a vector

Human adenoviruses, (AdHu), are attractive viral vectors. They possess a genetically stable virion so that inserts of foreign genes are not deleted. Also adenoviruses can infect large numbers of cells and the transferred information remains epichromosomal thus avoiding any potential for insertional mutagenesis. Replication defective adenovirus can be engineered by deletion of genes from the E1 locus, which is required for viral replication, and these viruses can be propagated easily with good yields in cell lines expressing E1 from AdHu5 such as human embryonic kidney cells 293, HEK 293 [28]. Previous mass vaccination campaigns in very large numbers of US military personnel using orally administered live human adenovirus serotype 4 and 7 have shown good safety and efficacy data [29].

Human adenoviruses were initially considered as viral vectors for gene therapy, notably in cystic fibrosis where in theory, the delivery of the normal CF gene to a percentage of the cells in the lung should result in correction of the defect and a prolonged life span for those with the disorder. However, several clinical trials in humans were carried out and it was found that transgene expression was only transient, (undetectable at 40 days), and the vector was potently immunogenic [30] [31]. When administered at higher doses, via a bronchoscope, (2.1 x 10^{11} vp), local pulmonary inflammation prevented any further dose escalation. Analysis of this potent immune response to adenoviral vectors indicated it was primarily a cytotoxic T cell response directed at the vector rather than the transgene and transduced cells expressing adenoviral antigens were eliminated in 3-4 weeks [32]. Tragically in 1999 one patient from a group of 18, died during a trial of liver directed gene transfer for partial ornithine transcarbamylase deficiency using an adenoviral vector. Doses in this trial were substantially higher than previously used, (3.6 x 10^{13} vp), and also were delivered directly into the right hepatic artery [33] [34].

Potent immunogenicity and lack of prolonged transgene expression have made adenoviruses of interest as vaccine vector candidates. Indeed, recent HIV-1 vaccine clinical trials conducted by Merck and the NIH show that very high levels of CD8+ T cells can be induced by adenovirus vector immunisation, more than observed in any other trials of any vaccine type [35]. Using irradiated sporozoite vaccination as a comparison, it was shown that a single vaccination with recombinant human adenovirus encoding CS of *Plasmodium yoelii* could reduce hepatic parasite load by 93% and engender sterile protection at sporozoite challenge in 40% of mice, compared to 20% of the irradiated sporozoite group [36]. Murine models using the Sin Nombre virus, comparing AdHu5 against recombinant vaccinia virus, both expressing SNV envelope proteins showed that AdHu5 generates higher numbers of epitope specific IFN gamma producing cells than rVV [37]. Attempts to increase the levels of protection against *P. yoelii* in mice through heterologous prime boost strategies involving AdHu5 prime and MVA boost both expressing the CS gene illustrated it was possible to induce complete protection at challenge using AdHu5 as a prime and MVA as a boost. Using AdHu5 in a prime boost strategy for HIV-1 gag showed that homologous boosting did not

improve the peak post prime levels of gag specific lymphocytes, probably due to anti-vector immunity [38].

A major limiting factor to the usage of human adenovirus as a vector is the level of anti vector immunity present in humans where adenovirus is a ubiquitous infection. Estimates suggest that, depending on the geographical region, between 45 – 80 % of adults carry AdHu5-neutralising antibodies [39]. Studies have shown that immunisation with AdHu vectors in animal models in the presence of pre-exposure to human adenoviruses attenuates responses to the vaccine, probably due to the removal of virus particles by pre-existing antibodies [40-42]. Phase 1 trials of a multiclade HIV-1 vaccine delivered by a replication defective AdHu5 have had to exclude volunteers with pre-existing antibodies to AdHu5 at titres greater than 1:12 [35]

3.6 Simian adenoviruses

The prevalence of immunity to human adenovirus prompted the consideration of simian adenoviruses as vectors. They exhibit hexon structures homologous to that of human adenoviruses (fig 1). Indeed, AdCh63 hexons are most similar in sequence to AdHu4 hexons previously used by the US military in mass vaccination campaigns where over 2 million adults received tablets of serially passaged adenovirus with good safety and efficacy data [43] (Personal Communication Col. John D. Grabenstein). Hexons are the major capsid proteins in adenoviruses; they are potently immunogenic and the main target of neutralising antibodies [44]. In chimpanzee adenoviruses the E1 locus can be deleted to render viruses replication deficient and allow transcomplementation on an E1 AdHu5 complementing cell line [45]. An additional attractive observation is that the lack of sequence homology between AdHu5 and simian adenoviruses at the E1 flanking sequence prevents homologous recombination and production of replication competent virus [46]. Simian adenoviruses are not known to cause pathological illness in humans and the prevalence of antibodies to chimpanzee origin adenoviruses is less than 5% in humans residing in the US [47]. In Equatorial Africa; the natural habitat for chimpanzees prevalence is higher but still below that to anti AdHu5 immunity. Early murine work using chimpanzee adenovirus 68, AdCh68, expressing *gag* of HIV-1 showed, that in comparison with AdHu5 (9%) and poxvirus (<5%), AdCh68 was as effective at generating a transgene product specific CD8+ T cell response, with approximately 20% of all splenic CD8+ being gag specific [48]. In the same study, pre-exposure to AdHu5 abolished any protection offered by immunisation with AdHu5 but only slightly reduced that elicited by AdCh68.

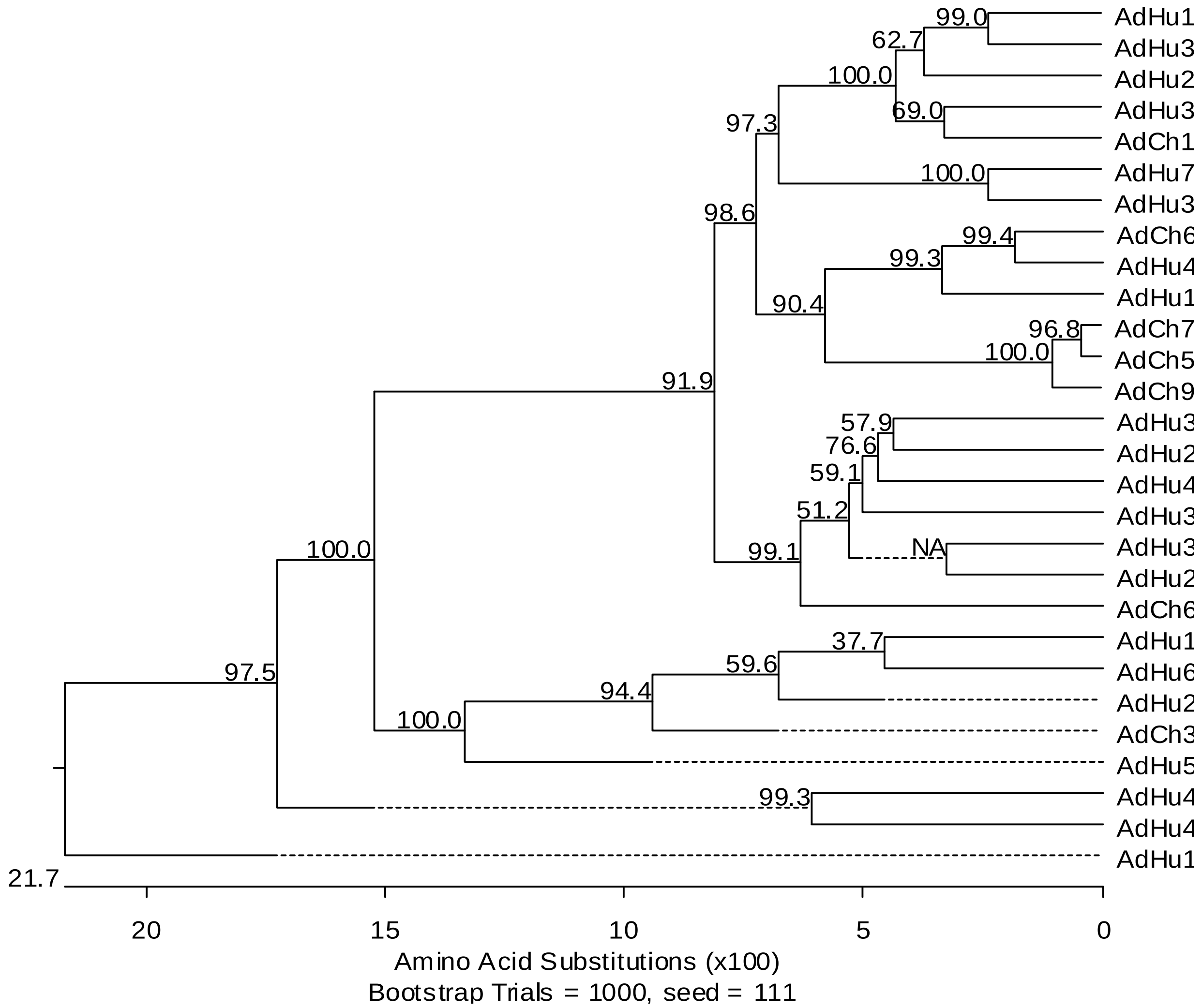

Figure 1. The phylogenetic tree of adenovirus hexons.

Using AdCh6, AdCh7 and AdCh68 in a mouse malaria model it has been shown that after a single immunisation with a simian adenoviral vector encoding ME-TRAP, sterile protection at sporozoite challenge was induced in 67 % - 92% of mice respectively. In contrast, MVA ME-TRAP and FP9 ME-TRAP alone offered no protection [49].

Measuring production of splenic peptide-specific interferon-γ (IFN-γ) secreting CD8+ T cells, (via ELISPOT assay and ICS), shows similar levels of immunogenicity in mice immunised with AdCh68 and AdCh63 expressing ME-TRAP. However at challenge with *P. berghei* AdCh63 generated 83% protection at 20 days compared to 30% protection in the AdCh68 group, (A.Reyes-Sandoval unpublished data).

More recently, extensive work has been undertaken in Oxford using adenovirus and poxvirus vectors encoding the blood-stage malaria antigens MSP-1 and AMA1 [14]. Immunising mice or rabbits with a single dose of AdCh9, AdCh7, AdCh6 or AdHu5, all expressing the blood-stage malaria antigen MSP-1, induces high levels of MSP-1_{19}-specific antibody responses. The antibody responses in the groups of mice or rabbits immunised with the simian adenoviruses are again

identical to those induced by AdHu5, showing that simian adenovirus vectors are a suitable replacement for AdHu5 as blood-stage malaria vaccine vectors (A. Goodman *et al.*, unpublished). Similarly, mice immunised with an Adhu5 vector expressing AMA1 also induce potent antibody responses against the target antigen (S. Biswas *et al.*, unpublished). These antibody responses can be boosted, by using MVA encoding the same antigen, to levels unprecedented for viral vectors – similar to many recombinant protein-in-adjuvant formulations [14]. Immunoglobulin purified from the serum of mice immunised with these MSP-1 vectors shows strong responses against both dimorphic alleles of MSP-1_{42} as measured using a standardised ELISA – endpoints of 205,154 against 3D7 strain and 99,908 against FVO strain of *P. falciparum*. Importantly, the purified IgG also yields very strong growth inhibitory activity in a standardised *in vitro* assay against *P. falciparum* – 46% inhibition against 3D7 and 52% against FVO [14]. These vectors also induce potent $CD4^+$ and $CD8^+$ T cell responses against the encoded MSP-1 antigen in BALB/c and C57BL/6 mice (A. Goodman *et al.*, unpublished). These cellular responses have been shown to be important in preclinical studies against *P. yoelii* for the induction of liver-stage immunity and for providing $CD4^+$ T cell help for B cell antibody responses [13].

Similar studies have demonstrated the potency of the simian adenovirus vectors encoding ME-TRAP. AdCh9 produced the highest levels $CD8^+$ cells, and provided 92% protection to a *P. berghei* sporozoite challenge 14 days later compared to 83% protection with AdHu5 and AdCh7. At 60 days however protection to challenge was reduced to 17% in the AdCh9 group, 50% in the AdCh7 group and was absent in all other groups (A. Reyes Sandoval, submitted). A comparison of AdCh63 and AdCh9 expressing ME-TRAP showed a similar level of splenic IFN-γ secreting cells in mice, but AdCh63 produced high responses when measured in the blood (>20,000 SFC/million PBMCs compared to 12.5 million for AdCh9). After a challenge with *P. berghei* sporozoites, AdCh63 showed 83% protection at 20 days compared to 33% in the AdCh9 group.

AdCh63 AMA1 will be manufactured under Good Manufacturing Practice conditions by the Clinical Biomanufacturing Facility (CBF), Churchill Hospital, Oxford.

Simian adenoviruses are currently being used as a vector for the first time in humans in malaria vaccine trials at the University of Oxford in the form of AdCh63 ME-TRAP, (a vaccine against the pre-erythrocytic stage of malaria) and AdCh63 MSP-1 (a vaccine against the erythrocytic stage of malaria) and in a hepatitis C vaccine trial by the same group in the form of AdCh3NSmut. They are also understood to be under development as vaccines for HIV (by GSK). Importantly, unlike vectors derived from some rare human serotypes of adenovirus, the simian vector used in this trial, AdCh63, appears in the pre-clinical studies to have similar potency to the AdHu5 strain. Many other human serotypes have been found to have lower potency.

Clinical Trials with Blood Stage Vaccines

As well as AMA1, a number of other blood-stage malaria antigens are currently being investigated in preclinical stages as potential blood stage vaccine candidates [4, 50]. These include

- MSP-1, MSP2, MSP3, MSP4, MSP5 and MSP9 (merozoite surface proteins or portions thereof either as recombinant or synthetic peptides)
- GLURP, (glutamate rich protein as a recombinant or synthetic protein)
- RAP2, (recombinant rhoptry-associated protein 2)
- EBA-175 and DBP, (erythrocyte binding proteins involved in parasite invasion)
- PfEMP1, (recombinant erythrocyte membrane protein, an antigen present on the surface of infected red blood cells.)

The first blood-stage antigen to enter clinical trials was the synthetic peptide vaccine, SPf66 which contained short peptide antigens from the blood-stage of malaria together with the pre-erythrocytic malaria antigen, circumsporozoite protein (CSP). Over 10 clinical trials were carried out with initially promising results, however further studies found its effects were small and indeed nonexistent in sub–Saharan Africa and Thailand [51].

Small scale clinical trials in Papua New Guinea of the ‘Combination B’ malaria vaccine, comprising three antigens - ring-infected erythrocyte surface antigen, (RESA), and merozoite surface proteins 1 and 2 (MSP-1 and MSP2) formulated in an oil-based adjuvant, demonstrated a 62% reduction in parasite density but no reduction in disease incidence in one subgroup of vaccinees [52]. The same vaccine was found to impact on the strain of malaria parasite found in vaccinees [53], suggesting that vaccination preferentially prevented infections with the same strain.

There have been three significant human clinical trials to date using the recombinant AMA1-C1/Alhydrogel® ± CpG vaccine at Johns Hopkins University (Baltimore, MD),[74, 94, 95] and a further trial in Australian adults and four in Malian adults or children. In the first US trial, 30 subjects received up to 3 immunisations (Day 0, 28 and 180) with either 5, 20, or 80 µg doses of AMA1-C1/Alhydrogel®.[96] Adverse events related to vaccination were limited to local injection site reactions, which were mild to moderate in severity. A dose response was noted, with the 80 µg group giving the highest antibody levels.

This led to a randomized, double blind, controlled trial of AMA1-C1/ Alhydrogel® in Mali. 54 healthy Malian adults were randomized 2:1 to receive either AMA1-C1/Alhydrogel® (at 5, 20, or 80 µg doses) or the Recombivax® Hepatitis B vaccine. Study participants received 3 immunisations on study days 0, 28, and 360. No vaccine-related serious or Grade 3 events were observed. Anti-

AMA1 antibody levels were assessed up to 2 weeks after the second immunisation in all dose groups, and a significant response was seen in the 80 µg dose group in comparison to those who received Recombivax®.[44]

The third trial was a Phase I/IIb randomized, controlled study of the safety and immunogenicity of AMA1-C1/Alhydrogel® in Malian children.[98] In Phase Ib, 36 children were randomized to receive either 20µg AMA1-C1/Alhydrogel®, 80 µg AMA1-C1/Alhydrogel® or the comparator vaccine (Hiberix®) with two doses given one month apart. Phase IIb enrolled an additional 300 children, who were followed through the malaria transmission season to assess biological impact. No impact of vaccination was seen on the primary endpoint; the frequency of parasitaemia measured as episodes >3000/microL/day at risk. There was a negative impact of vaccination on haemoglobin during clinical malaria, with a mean incidence of haemoglobin <8.5 g/dL in children who received AMA1-C1, although these differences were not significant after correction for multiple tests.[98] Studies adjuvanting the AMA1-C1/Alhydrogel® vaccine with CPG 7909 oligonucleotide have completed two phase Ia trials in the USA and one Phase Ib trial in Malian adults, demonstrating enhanced antibody production and growth inhibition in *in vitro* assays.[72-74, 95] A phase I/IIa study of the safety, immunogenicity, parasite growth inhibitory activity and protective efficacy of AMA1-C1/Alhydrogel® + CPG 7909 is currently underway in Oxford.

Field trials are also being carried out in Mali using an AMA1 based recombinant protein vaccine adjuvanted with AS02A (FMP2.1/AS02A).[75] This follows a promising phase Ia trial where all 23 volunteers seroconverted, but showed disappointing results in a subsequent Phase IIa sporozoite challenge study.[76, 77] Antibodies produced were capable of inhibiting parasite growth *in vitro* and volunteers had measurable FMP2.1 specific lymphocytes on IFN gamma ELIspot.

A more recent study assessed the safety and immunogenicity of recombinant AMA1 (FVO allele) in a dose-escalating Phase Ia trial.[71] 8-10 volunteers received three 10µg or 50µg doses at 4-week intervals with three different adjuvants; Alhydrogel, Montanide ISA720 and AS02. Antibody responses were highest in the AS02 groups, and these showed activity in an *in vitro* GIA up to 80%. All formulations showed distinct and in some cases marked reactogenicity profiles, which were more apparent for the Montanide ISA 720 and AS02 adjuvant groups.[71]

A more recent study assessed the safety and immunogenicity of recombinant AMA1 (FVO allele) in a dose-escalating phase Ia trial. 8-10 volunteers received three 10µg or 50µg doses at 4-week intervals with three different adjuvants, Alhydrogel, Montanide ISA720 and AS02. Antibody responses were highest in the AS02 groups, and these showed activity in an *in vitro* growth inhibition assay up to 80%. All formulations showed distinct and in some cases marked reactogenicity profiles, which were more apparent for the Montanide and AS02 adjuvant groups [61].

A long synthetic peptide derived from MSP-3, containing B- and T-cell epitopes, was tested in Burkina Faso, and generated long-lasting antibodies that show *in vitro* activity [63].

Currently a phase I/IIa trial is underway in the US examining the use of human adenovirus serotype 5 as a vector expressing CS and / or AMA1, however no data are as yet published.

Clinical Trials in Oxford:

Since August 1999 a series of Phase I clinical studies in Oxford have assessed the prime-boost immunisation approach in healthy, malaria-naïve human volunteers. These studies have used different vectors, including MVA, FP9 and AdCh63. The ‘inserts’ or antigens inserted into these vaccines include ME-TRAP, (a surface protein from the sporozoite stage of *P. falciparum* malaria with a selection of T-cell epitopes) CSO or CS, (circumsporozoite protein, again from *P. falciparum* malaria) and L3SEPTL (six pre-erythrocytic antigens linked together to produce a 3240-aa-long polyprotein) [64]. Studies using these vectors and inserts in various combinations have demonstrated safety and immunogenicity, with significant *ex vivo* ELISPOT responses [65-67]. Following immunisation, most subjects then participate in a subsequent study in Oxford where they are challenged with the 3D7 strain of *P. falciparum* (a fully drug sensitive strain) under close observation to assess vaccine efficacy. To date our vaccines have targeted pre-erythrocytic diseases with some success [65, 68, 69].

Human clinical trials using a plasmid DNA vectored vaccines against malaria were carried out as early as 1998 where a lone DNA vaccine was trialed. This generated moderate immunogenicity but no protection against malaria [70]. Initial studies in Oxford elected to use a heterologous prime boost approach to try and amplify the immune response and increase protection. The earliest trials used a DNA-MVA combination, both expressing ME-TRAP. This combination proved strikingly immunogenic, producing over 1000 antigen specific cells per million peripheral blood mononuclear cells with three DNA priming immunisations. At challenge vaccinees showed a delay to parasitemia, indicating partial protection [71]. A phase IIb trial in The Gambia, however, did not show any significant protection against malaria infection in semi-immune adults [72].

Following murine work which showed FP9-MVA in a prime boost strategy was more immunogenic and protective than DNA-MVA [73], clinical trials using this approach were carried out. FP9-MVA demonstrated promising results, including complete protection against malaria challenge in the initial two out of five vaccinated subjects who had received two doses of FP9 ME-TRAP followed by one dose of MVA ME-TRAP [69]. Furthermore, several immunisation regimes using the ME-TRAP insert have led to highly statistically significant delays in time to patient parasitaemia. Analysis of this delay shows that it corresponds to a vaccine-induced, greater than 80% reduction in liver stage parasite number on day 6.5 post-sporozoite challenge for DNA-MVA and about a 92% reduction with FP9-MVA [74]. However, even the latter regime fails to induce sterile immunity

in the great majority of vaccinees. Importantly, however these studies have identified a cellular immunological correlate of protective efficacy: vaccinees with T cell responses > 1000 SFU/million PBMCs were very likely to be protected.

To date only two trials in Oxford have examined the effect of blood stage antigens on humans. The first used a virosomal vaccine formulation, PEV3A, which included two cyclised peptides from CS and AMA1 on the surface of virosomes [23]. During this trial following sporozoite challenge we did not observe sterile protection in any volunteers, however parasite growth rate was significantly reduced in the blood prior to patency, one volunteer had a very late diagnosis (day 20 compared to 11.8 days in unvaccinated controls,), and PCR results from PEV3A vaccinated volunteers showed evidence of early control of parasitemia by some volunteers [62]. The significant reduction in growth rates of parasite in the blood of vaccines provides the first evidence of protection provided by a blood-stage malaria vaccine in any challenge study. Because CS is not a blood-stage antigen it is clear that protection must have been caused by the AMA1 component of the vaccine, which is from the domain III region of the ectodomain. This result strongly supports the development of further AMA1 vaccines that may have greater protective efficacy.

The second trial in Oxford examining the effect of blood stage antigens on humans uses AdCh63 and MVA as viral vectors encoding the antigen MSP-1 and is currently underway (VAC037 – interim safety data below).

The AdCh63 AMA1 vaccine we intend to use should further improve on current trials by using a potently immunogenic simian adenovirus which will circumvent problems linked to pre-existing immunity in humans to AdHu vectors and also induce potent antibody and T cell response to our candidate antigen AMA1.

Current Clinical Trials using simian adenoviruses

1. **Liver Stage Malaria Vaccines**

AdCh63 ME-TRAP & MVA ME-TRAP – Safety Data

AdCh63 is currently in use with the ME-TRAP insert, a liver stage antigen in a phase I dose escalation clinical trial in Oxford (VAC033 / GTAC133) and a phase IIa trial with sporozoite challenge (MAL034 / OXREC: 09/H064/9). 38 volunteers have received AdCh63 ME-TRAP alone at doses from 1 x 10^8 vp to 2 x 10^{11} vp and 47 volunteers have received AdCh63 ME-TRAP at doses from 1 x 10 vp to 2 x 10^{11} vp, followed by MVA ME-TRAP boosting with no serious adverse events (see safety data below). AdCh63 ME-TRAP administered intramuscularly is better tolerated and more immunogenic than the same dose administered by the intradermal route (O'Hara, Duncan et al, manuscript in preparation).

MVA ME-TRAP has been administered to more than 700 volunteers in the UK, Gambia and Kenya in prime-boost regimes with no serious adverse events to date [23-25, 52, 102, 103]. MVA ME-TRAP has traditionally been administered by the intradermal route with significant but transient local injection-site reactions (see safety data below). We have recently demonstrated that there is no significant attenuation of immunogenicity when MVA ME-TRAP is administered intramuscularly but there is a significant reduction in the frequency of local injection site reactions (O'Hara, Duncan et al, manuscript in preparation).

The simian adenovirus/MVA-vectored heterologous prime-boost regime generates unprecedented cellular and humoral immune responses to TRAP and has recently demonstrated sterile protection against heterologous sporozoite challenge in 2/8 vaccinated volunteers (O'Hara et al, submitted). A repeat of this sporozoite challenge study with an additional 12 prime-boost vaccinated volunteers is underway in which the protected volunteers will be re-challenged in parallel to assess the durability of the vaccine-induced protective efficacy. Initial results are expected in early March 2010 (Duncan et al, unpublished).

Safety Data (Interim analysis 8th Feb 2010)

There have been no serious adverse events (SAEs) following AdCh63 ME-TRAP or MVA ME-TRAP to date. Safety data from two current trials MAL034 (phase IIa efficacy trial) and VAC033 (phase I safety trial) are presented below.

MAL034 Safety Data

Adverse Event Number of events (% of volunteers)	5×10^{10} vp AdCh63 ME-TRAP I.M. Number of events (% of volunteers) N = 31			2×10^{8} pfu MVA ME-TRAP I.D. Number of events (% of volunteers) N = 21		
	Mild	Moderate	Severe	Mild	Moderate	Severe
Local pain	20 (65)	0	0	14 (62)	5 (24)	0
Local swelling	7 (23)	0	0	10 (46)	7 (33)	4 (20)
Local erythema	10 (32)	0	0	10 (46)	11 (54)	0
Local warmth	1 (3)	0	0	19 (91)	0	0
Local pruritus	3 (10)	0	0	18 (86)	0	0
Local scaling	0	0	0	12 (57)	0	0
Local adenopathy	0	0	0	1 (5) Supraclavicular<br>3 (14) Axillary	0	0
Axillary tenderness	1 (3)	0	0	3 (14)	0	0
Headache	19 (61)	0	1 (3)	12 (57)	0	0
Malaise	6 (20)	0	0	8 (38)	0	0
Myalgia	4 (13)	0	0	5 (24)	0	0
Fatigue	13 (42)	0	0	11 (52)	0	0
Feverishness	8 (26)	0	0	8 (38)	0	0
Fever	2 (6)	2 (6)	0	3 (14)	3 (14)	0
Arthralgia	5 (16)	0	0	7 (34)	1 (5)	0
Nausea	3 (10)	1 (3)	0	5 (24)	0	0
Coryza	2 (6)	0	0	3 (14)	0	0
Urticaria	2 (1)	0	0	0	0	0
Sore throat	3 (6)	0	0	1 (5)	0	0
Back pain	1 (3)	0	0	1 (5)	0	0
Migraine	0	0	0	1 (5)	0	0

Table 1 *All solicited and unsolicited local and systemic adverse events considered possibly, probably or definitely vaccine-related, MAL 034. Interim analysis at 8th Feb 2010.*

The vast majority of local and systemic adverse events (AEs) following AdCh63 ME-TRAP were mild in severity (94%) and all resolved completely. Overall 75% of volunteers experienced one or more systemic AEs and 75% one or more local injection-site reactions (which were all mild). There were three moderate-grade systemic AEs: two occurred in one volunteer - an episode of vomiting 48 hours post-vaccination which lasted for less than 12 hours, which was considered probably related to vaccination, and a short-lived fever of 38.3C on the evening of vaccination which resolved by the following morning. Although the episode of vomiting was considered probably vaccine-related due to the close proximity to vaccination, the volunteer described a household contact that had also experienced a short-lived episode of vomiting and it is therefore more likely this was actually associated with food poisoning. This volunteer also experienced a severe headache on the evening of vaccination which was migranous in nature and was considered definitely related to vaccination. This required oral paracetamol and resolved the following morning. The final moderate AE was a fever of 38.4C in a separate volunteer on the evening of vaccination which resolved the following morning. The AE profile of AdCh63 ME-TRAP is very similar to that extensively described for human adenovirus vectored vaccines.

MVA ME-TRAP was more reactogenic than AdCh63 ME-TRAP and this was largely due to an increase in the frequency and severity of local reactions. Overall 90% of volunteers experienced one or more systemic AEs and all experienced one or more injection-site reactions. Four AEs were considered severe post-MVA ME-TRAP; all were local swelling (as defined by swelling of > 5cm in diameter). The mean maximal diameter of severe swelling in these volunteers was 7.45 cm and the mean duration of severe intensity was 3.25 days, with a mean duration of 11.5 days in total. All post MVA adverse events resolved. The mean duration of all systemic adverse events was 48 hours; the mean duration of all local adverse events was 5 days. Moderate erythema (54% of volunteers), swelling (33% of volunteers) and pain (24% of volunteers) were also observed. Three volunteers experienced moderate-grade fever (all < 38.5C) for less than 24 hours. One volunteer also experienced moderate-grade arthralgia for less than 24 hours which resolved with paracetamol.

VAC033 Safety Data

Full safety data for AdCh63 ME-TRAP at various doses and routes is summarised in Tables 2 & 3. The intramuscular route is associated with significantly reduced frequency of injection-site reactogenicity (Figure 7). With AdCh63 ME-TRAP 5×10^{10} vp intramuscularly (the dose used in MAL034, given to 8 volunteers) 62.5% of volunteers experienced at least one local AE and 62.5% at least one systemic AE. There were no severe AEs and only one moderate fever (< 38.5C). This is comparable to the reactogenicity profile seen in MAL034 described above. Four adverse events following AdCh63 ME-TRAP in VAC033 were graded as severe; these occurred in the same individual in an earlier group 13 days following administration of 1×10^{10} vp by the intradermal

route. These were a cluster of symptoms associated with an upper respiratory tract infection including fever, malaise, coryza and headache. Although recorded by the volunteer as moderate in severity, they resulted in one day of absenteeism from employment; therefore a grading of severe was attributed. These symptoms resolved completely within one week, and were considered possibly related to vaccination due to the fact they occurred within 14 days post-vaccination, although were more likely related to an inter-current upper respiratory tract infection. At the highest dosage of AdCh63 ME-TRAP (2 x 10^{11} vp, administered intramuscularly) the frequency and intensity of local and systemic AEs increased. 90% of volunteers experienced a local injection site reaction the most frequent of which was pain. This was of moderate severity in 40% of volunteers. All volunteers experienced at least one systemic AE. The frequency of fever increased to 60% however fever was of moderate severity (>37.9C) in 2/10 volunteers. In addition one volunteer experienced a cluster of ‘flu-like symptoms (feverishness, myalgia and arthralgia) 12 hours post vaccination which persisted for 24 hours, required paracetamol, and resulted in one day of absenteeism from employment, and were therefore graded as severe. All AEs fully resolved. The mean duration of both injection-site and systemic reactions and was 48 hours. There were no SAEs.

**Adverse Event**	**AdCh63 ME-TRAP Route and Dose**						
	**Intradermal**				**Intramuscular**		
	**1 x 10^8 (n = 8)**	**1 x 10^9 (n = 8)**	**1 x 10^{10} (n = 8)**	**5 x 10^{10} (n = 8)**	**1 x 10^{10} (n = 4)**	**5 x 10^{10} (n = 8)**	**2 x 10^{11} (n = 10)**
**Pain – No. (%)**							
**Mild**	2 (25)	4 (50)	4 (50)	5 (62.5)	2 (50)	4 (37.5)	5 (50)
**Moderate**	1 (12.5)	0	0	1 (12.5)	0	0	4 (40)
**Severe**	0	0	0	0	0	0	0
**Erythema – No. (%)**							
**Mild**	8 (100)	7 (87.5)	8 (100)	8 (100)	2 (50)	2 (25)	2 (20)
**Moderate**	0	0	0	0	0	0	0
**Severe**	0	0	0	0	0	0	0
**Swelling – No. (%)**							
**Mild**	7 (87.5)	7 (87.5)	8 (100)	8 (100)	1 (25)	1 (12.5)	2 (20)
**Moderate**	0	0	0	0	0	0	0
**Severe**	0	0	0	0	0	0	0
**Warmth – No. (%)**							
**Mild**	2 (25)	3 (37.5)	5 (62.5)	6 (75)	1 (25)	1 (12.5)	5 (50)
**Moderate**	0	0	0	0	0	0	0
**Severe**	0	0	0	0	0	0	0
**Scaling – No. (%)**							
**Mild**	3 (37.5)	2 (25)	5 (62.5)	6 (75)	1 (25)	0	0
**Moderate**	0	0	0	0	0	0	0
**Severe**	0	0	0	0	0	0	0
**Pruritus – No. (%)**							
**Mild**	2 (25)	4 (50)	5 (62.5)	7 (87.5)	0	1 (12.5)	0
**Moderate**	0	0	0	0	0	0	0
**Severe**	0	0	0	0	0	0	0
**Blistering – No. (%)**	0	0	0	0	0	0	0
**Total – No. (%)**							
**Mild**	24 (100)	27 (100)	35 (100)	40 (100)	7 (50)	9 (62.5)	14 (90)
**Moderate**	1 (12.5)	0	0	1 (12.5)	0	0	4 (40)
**Severe**	0	0	0	0	0	0	0

Table 2 *Local solicited adverse events following AdCh63 ME-TRAP considered possibly, probably or definitely vaccine-related. The first episode of each AE is recorded. The highest-intensity AE per subject is listed. Data from ongoing phase I dose finding study, VAC 033.*

Adverse Event	AdCh63 ME-TRAP Route and Dose						
	Intradermal				Intramuscular		
	1 x 10^8 (n = 8)	1 x 10^9 (n = 8)	1 x 10^{10} (n = 8)	5 x 10^{10} (n = 8)	1 x 10^{10} (n = 4)	5 x 10^{10} (n = 8)	2 x 10^{11} (n = 10)
**Headache – No. (%)**							
**Mild**	1 (12.5)	1 (12.5)	1 (12.5)	5 (62.5)	2 (50)	5 (50)	8 (70)
**Moderate**	1 (12.5)	0	0	1 (12.5)	0	0	0
**Severe**	0	0	1 (12.5)	0	0	0	0
**Malaise – No. (%)**							
**Mild**	1 (12.5)	2 (25)	3 (37.5)	5 (62.5)	2 (50)	6 (62.5)	6 (60)
**Moderate**	0	0	0	1 (12.5)	0	0	0
**Severe**	0	0	1 (12.5)	0	0	0	0
**Fatigue – No. (%)**							
**Mild**	4 (50)	2 (25)	8 (100)	6 (62.5)	2 (50)	5 (50)	8 (80)
**Moderate**	0	0	0	1 (12.5)	0	0	0
**Severe**	0	0	0	0	0	0	0
**Myalgia – No. (%)**							
**Mild**	1 (12.5)	1 (12.5)	4 (50)	3 (37.5)	0	3 (37.5)	7 (70)
**Moderate**	0	0	0	0	0	0	0
**Severe**	0	0	0	0	0	0	1 (10)
**Arthralgia – No. (%)**							
**Mild**	1 (12.5)	1 (12.5)	3 (37.5)	3 (37.5)	0	2 (25)	6 (60)
**Moderate**	0	0	0	0	0	0	0
**Severe**	0	0	0	0	0	0	1 (10)
**Pyrexia – No. (%)**							
**Mild**	1 (12.5)	0	1 (12.5)	3 (37.5)	1 (12.5)	1 (12.5)	3 (30)
**Moderate**	0	1 (12.5)	0	0	0	1 (12.5)	3 (20)
**Severe**	0	0	1 (12.5)	0	0	0	0
**Feverish – No. (%)**							
**Mild**	1 (12.5)	1 (12.5)	3 (37.5)	4 (50)	0	4 (50)	6 (60)
**Moderate**	1 (12.5)	0	1 (12.5)	1 (12.5)	0	0	0
**Severe**	0	0	0	0	0	0	1 (10)
**Nausea – No. (%)**							
**Mild**	0	0	3 (37.5)	2 (25)	1 (12.5)	0	5 (50)
**Moderate**	0	0	0	0	0	0	0
**Severe**	0	0	0	0	0	0	0
**Adenopathy – No. (%)**							
**Mild**	0	0	0	1 (12.5)	0	0	0
**Moderate**	0	0	1 (12.5)	0	0	0	0
**Severe**	0	0	0	0	0	0	0
**Total – No. (%)**							
**Mild**	10 (62.5)	8 (62.5)	26 (100)	32 (75)	8 (75)	26 (62.5)	49 (100)
**Moderate**	2 (12.5)	1 (12.5)	1 (12.5)	4 (12.5)	0	1 (12.5)	3 (20)
**Severe**	0	0	3 (12.5)	0	0	0	3 (10)

Table 3 *Systemic solicited adverse events following AdCh63 ME-TRAP considered possibly, probably or definitely vaccine-related. The first episode of each AE is recorded. The highest-intensity AE per subject is listed. Data from ongoing phase I dose finding study, VAC 033.*

Full safety data for **MVA ME-TRAP** at 2 x 10^8 pfu by administered by intradermal and intramuscular routes is summarised in Tables 4-5. The intramuscular route is associated with significantly reduced frequency of injection-site reactogenicity (Figure 7).

22 Volunteers have been vaccinated with 2 x 10^8 pfu MVA ME-TRAP by the intradermal route in VAC033. Local reactogenicity post MVA ME-TRAP is presented in Table 4, according to dose of AdCh63 ME-TRAP received. Four injection-site reactions were classed as severe; including 2 episodes of swelling (> 50 mm) an episode of pain at the vaccine site and a report of warmness to touch. These all occurred in volunteers primed by the intradermal route. The episodes of swelling occurred immediately following intradermal MVA ME-TRAP, peaking in diameter at 60mm and 55mm respectively and reducing to 4mm and 7mm respectively within 7 days. The total duration of

detectible swelling was 49 and 37 days respectively. No specific action was required. The episode of transient pain occurred immediately following vaccination requiring oral analgesia, reduced in intensity to moderate within 24 hours and had resolved by day 9 post vaccination. The episode of transient severe local warmth settled in intensity within 24 hours and persisted for 4 days post vaccination. All episodes fully resolved.

Injection-site reactions post intradermal MVA ME-TRAP were more persistent than with intradermal AdCh63 ME-TRAP but in the majority of cases have resolved by the D140 visit (3 months post vaccination with MVA ME-TRAP) where a faint papule is visible at the site of vaccination.

When administered by the intramuscular route (to 4 volunteers) the frequency of injection-site reactions was significantly attenuated (Figure 2) and the duration and severity of injection-site reactions was reduced. The majority of injections site reactions were of mild severity, and resolved within a mean of 3.5 days. There were no persistent visible skin changes when MVA ME-TRAP was administered intramuscularly.

**Adverse Event**	**MVA ME-TRAP 2 x 10^8 pfu I.D. Boost**						**MVA ME-TRAP 2 x 10^8 pfu I.M. Boost**
**Number**	*AdCh63 ME-TRAP Priming (All I.D. administration)*				*AdCh63 ME-TRAP Priming (All I.M. administration)*		*AdCh63 ME-TRAP Priming (All I.M. administration)*
	**1 x 10^8 (n = 4)**	**1 x 10^9 (n = 4)**	**1 x 10^{10} (n = 4)**	**5 x 10^{10} (n = 4)**	**5 x 10^{10} (n = 4)**	**2 x 10^{11} (n = 2)**	**2 x 10^{11} (n = 4)**
**Pain**							
**Mild**	3	3	3	3	3	2	3
**Moderate**	1	0	1	0	0	0	1
**Severe**	0	0	0	1	0	0	0
**Erythema**							
**Mild**	2	3	2	2	4	2	2
**Moderate**	2	1	2	2	0	0	0
**Severe**	0	0	0	0	0	0	0
**Swelling**							
**Mild**	3	2	2	2	1	2	0
**Moderate**	0	1	2	1	3	0	0
**Severe**	1	1	0	0	0	0	0
**Warmth**							
**Mild**	2	2	3	3	3	1	0
**Moderate**	0	0	1	1	0	0	0
**Severe**	0	1	0	0	0	0	0
**Scaling**							
**Mild**	4	4	3	2	2	1	0
**Moderate**	0	0	0	1	0	0	0
**Severe**	0	0	0	0	0	0	0
**Pruritus**							
**Mild**	4	4	4	2	3	2	0
**Moderate**	0	0	0	1	0	0	0
**Severe**	0	0	0	0	0	0	0
**Blistering**							
**Mild**	0	0	0	0	0	0	0
**Moderate**	0	0	0	0	0	0	0
**Severe**	0	0	0	0	0	0	0
**Total – No. (%)**							
**Mild**	18 (100)	18 (100)	17 (100)	14 (100)	16 (100)	10 (100)	5 (100)
**Moderate**	3 (50)	2 (25)	6 (50)	6 (75)	3 (75)	0	1 (25)
**Severe**	1 (25)	2 (50)	0	1 (25)	0	0	0

Table 4 *Local solicited adverse events following MVA ME-TRAP considered possibly, probably or definitely vaccine-related. The first episode of each AE is recorded. The highest-intensity AE per subject is listed. Data from ongoing phase I dose finding study, VAC 033.*

Four systemic adverse events post intradermal MVA ME-TRAP were grade 3 (severe). All occurred in volunteers primed with intradermal AdCh63 ME-TRAP. Three occurred in a volunteer primed with AdCh63 ME-TRAP 1 x 10^8 vp intradermally (two episodes of adenopathy and one of feverishness), and one in a volunteer primed with AdCh63 ME-TRAP 5 x 10^{10} vp intradermally (an episode of severe muscular spasm – torticollis). In the first volunteer both cervical and axillary adenopathy occurred immediately post vaccination and were considered severe by investigators; these episodes of adenopathy resolved without action within 15 days. This volunteer experienced a single episode of grade 3 feverishness with a report of rigors approximately 12 hours post vaccination, although no fever was documented. This resolved within 24 hours and did not recur. The episode of torticollis experienced by the second volunteer occurred several hours post vaccination and had resolved with physiotherapy and muscle relaxants by day 11 post-vaccination. There were no moderate or severe systemic AEs following intramuscularly administered MVA ME-TRAP.

Adverse Event	MVA ME-TRAP 2 x 10^8 pfu I.D. Boost						MVA ME-TRAP 2 x 10^8 pfu I.M. Boost
**Number**	*AdCh63 ME-TRAP Prime (All I.D. administration)*				*AdCh63 ME-TRAP Priming (All I.M. administration)*		*AdCh63 ME-TRAP Priming (All I.M. administration)*
	**1 x 10^8 (n = 4)**	**1 x 10^9 (n = 4)**	**1 x 10^{10} (n = 4)**	**5 x 10^{10} (n = 4)**	**5 x 10^{10} (n = 4)**	**2 x 10^{11} (n = 2)**	**2 x 10^{11} (n = 4)**
**Headache**							
**Mild**	1	1	2	1	2	1	2
**Moderate**	1	0	1	0	0	0	0
**Severe**	0	0	0	0	0	0	0
**Malaise**							
**Mild**	0	1	3	2	2	0	1
**Moderate**	1	0	0	0	0	0	0
**Severe**	0	0	0	0	0	0	0
**Fatigue**							
**Mild**	1	2	2	3	2	2	1
**Moderate**	0	0	1	0	0	0	0
**Severe**	0	0	0	0	0	0	0
**Myalgia**							
**Mild**	1	1	2	1	2	2	2
**Moderate**	0	0	0	0	0	0	0
**Severe**	0	0	0	0	0	0	0
**Arthralgia**							
**Mild**	3	1	3	1	2	2	1
**Moderate**	0	0	0	0	0	0	0
**Severe**	0	0	0	0	0	0	0
**Pyrexia**							
**Mild**	1	0	0	0	0	0	0
**Moderate**	0	0	1	1	0	0	0
**Severe**	0	0	0	0	0	0	0
**Feverish**							
**Mild**	1	1	3	2	1	1	1
**Moderate**	0	0	1	0	0	0	0
**Severe**	1	0	0	0	0	0	0
**Nausea**							
**Mild**	1	1	3	1	0	0	0
**Moderate**	0	0	0	0	0	0	0
**Severe**	0	0	0	0	0	0	0
**Adenopathy**							
**Mild**	1	1	0	0	0	0	0
**Moderate**	0	0	0	0	0	0	0
**Severe**	1	0	0	0	0	0	0
**Total – No.**							
**Mild**	10 (100)	9 (75)	18 (100)	11 (75)	11 (75)	8 (100)	8 (50)
**Moderate**	2 (25)	0	4 (25)	1 (25)	0	0	0
**Severe**	2 (25)	0	0	1 (25)*	0	0	0

Table 5 *Systemic solicited adverse events following MVA ME-TRAP considered possibly, probably or definitely vaccine-related. The first episode of each AE is recorded. The highest-intensity AE per subject is listed. *Refers to acute torticollis. Data from ongoing phase I dose finding study, VAC 033.*

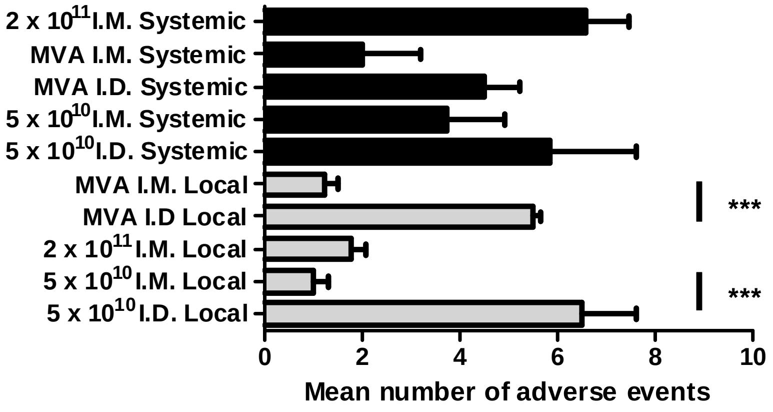

Figure 2 *Mean number of AEs per volunteer in each dose group, emphasizing the reduction in frequency of injection-site reactions with the use of the intramuscular route for both vaccines AdCh63 ME-TRAP and MVA ME-TRAP.*
MVA = MVA ME-TRAP
2 x 10^{11} = AdCh63 ME-TRAP 2 x 10^{11} vp
5 x 10^{10} = AdCh63 ME-TRAP 5 x 10^{10} vp
I.M. = Intramuscular route
I.D. = Intradermal route
*Bars represent mean with error bars representing SEM. Comparison of means by paired Student's t Test. *** $P < 0.01$.*

AdCh63 ME-TRAP and MVA ME-TRAP - Immunogenicity and Efficacy Data (Interim analysis VAC033 and MAL034)

Heterologous prime-boost with AdCh63 ME-TRAP and MVA ME-TRAP generates unprecedented liver-stage antigen-specific cellular immune responses of a magnitude 5-10 times greater than previous vectored sub-unit T cell inducing malaria vaccines (Figures 3 & 4). The vaccine-induced immune responses are of a greater magnitude when the priming vaccination AdCh63 ME-TRAP is administered by the intramuscular route (Figures 5 & 6). There is no influence of the route of MVA ME-TRAP on the immunogenicity of the boost (Figure 7). Increasing the dose of AdCh63 ME-TRAP to 2 x 10^{11} vp does not significantly increase the vaccine-induced immune response (Figure 8).

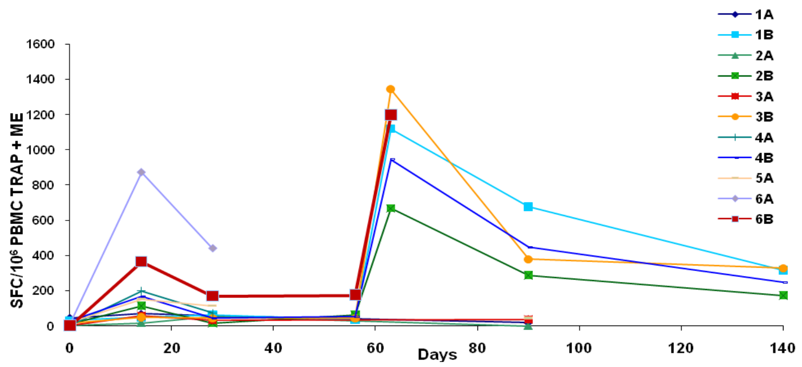

Figure 3 *VAC 033 Interim immunogenicity. Each line represents group mean ELISPOT responses to ME+TRAP (n = 4 in each group, n = 2 in group 6A). 'A' groups received AdCh63 ME-TRAP alone. 'B' groups received MVA ME-TRAP boost 8-16 weeks post prime.*

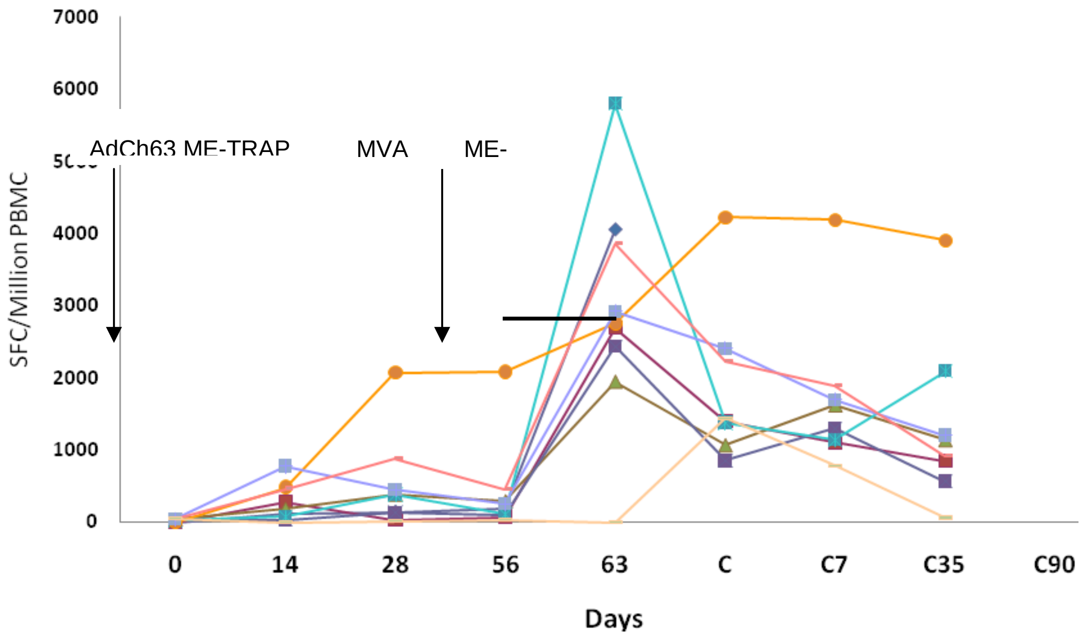

Figure 4 *MAL 034 Interim immunogenicity. Horizontal line represents mean of group response at day 63, each coloured line one individual volunteer's ELISPOT response to pooled ME-TRAP antigens.*

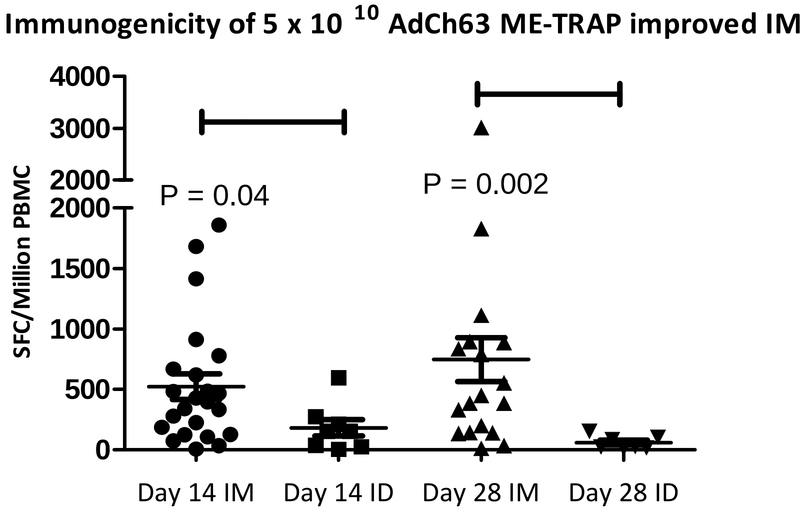

Figure 5 *Comparison of median Day 14 and Day 28 ELISPOT responses AdCh63 ME-TRAP administered IM (n = 23) versus ID (n = 8) at the same dose, by Mann-Whitney U Test. Data from VAC033 and MAL034*

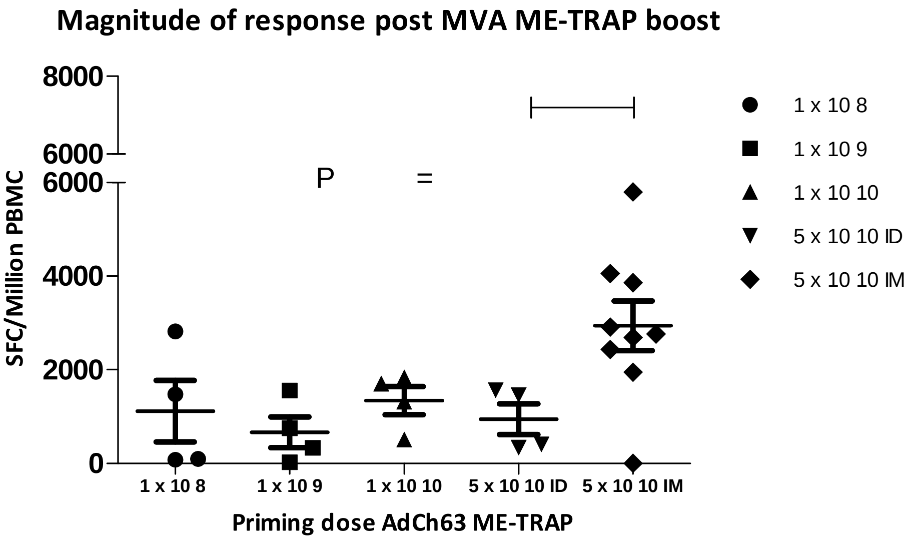

Figure 6 *Magnitude of mean post-boost ELISPOT responses by dosage of AdCh63 ME-TRAP priming vaccination, by Unpaired two-tailed Student's t test. N = 4 for each dose group ID, n = 9 for 5 x 10^{10} IM Adch63 ME-TRAP. Data from VAC033 and MAL034*

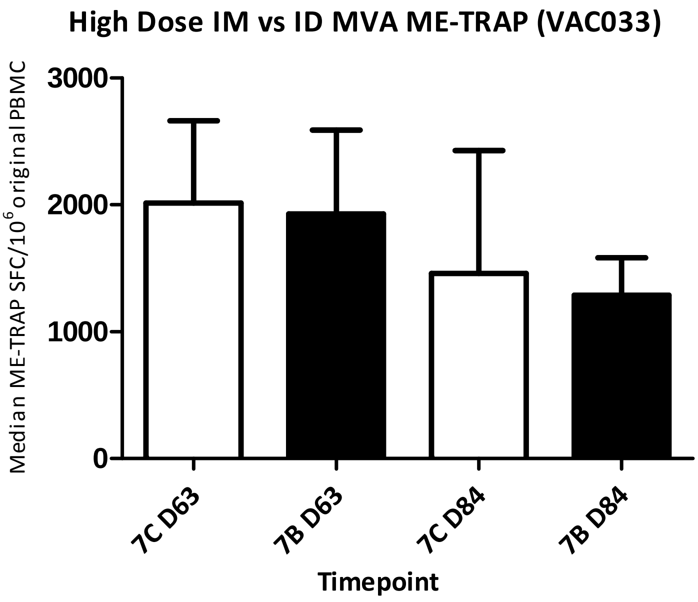

Figure 7 *Influence of MVA ME-TRAP route in VAC033.Filled bars represent I.D. MVA ME-TRAP, clear bars represent I.M. MVA ME-TRAP. No significant differences between key time-points post-boost identified (comparison of medians by Mann Whitney U test).*

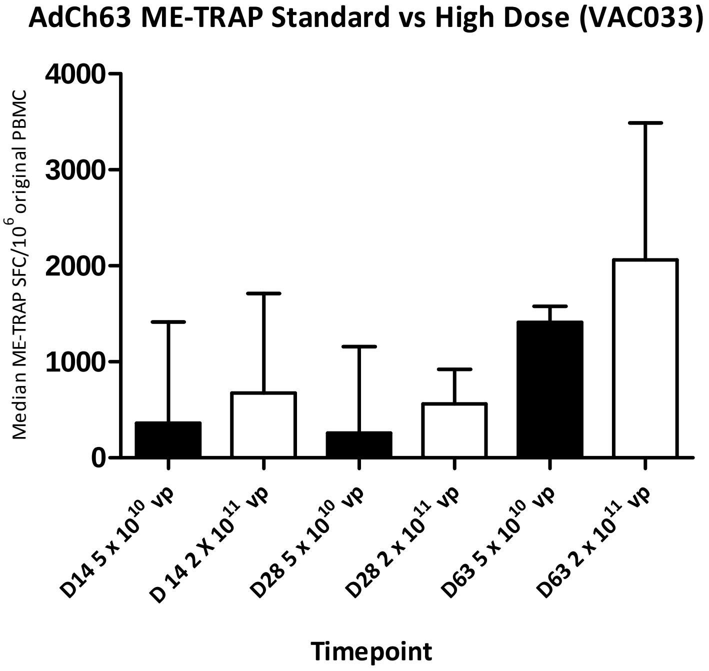

Figure 8 *Magnitude of antigen-specific responses following AdCh63 ME-TRAP at standard dose (5 x 10^{10} vp, filled bars) compared to high dose (2 x 10^{11} vp, clear bars) in VAC033. No significant differences between key time-points identified (comparison of medians by Mann Whitney U test).*

Eight volunteers in group 1 (AdCh63 ME-TRAP, MVA ME-TRAP prime-boost) and 10 volunteers in group 2 (AdCh63 ME-TRAP alone) of MAL 034 underwent malaria sporozoite challenge to determine vaccine efficacy. Two volunteers in group 1 (25% of those challenged) were completely protected against malaria infection (Figure 9). Protective efficacy in these volunteers was significantly correlated with the frequency of CD8+ T cells producing IFN-gamma quantified by flow cytometry (data not shown). Further immunological analysis is ongoing.

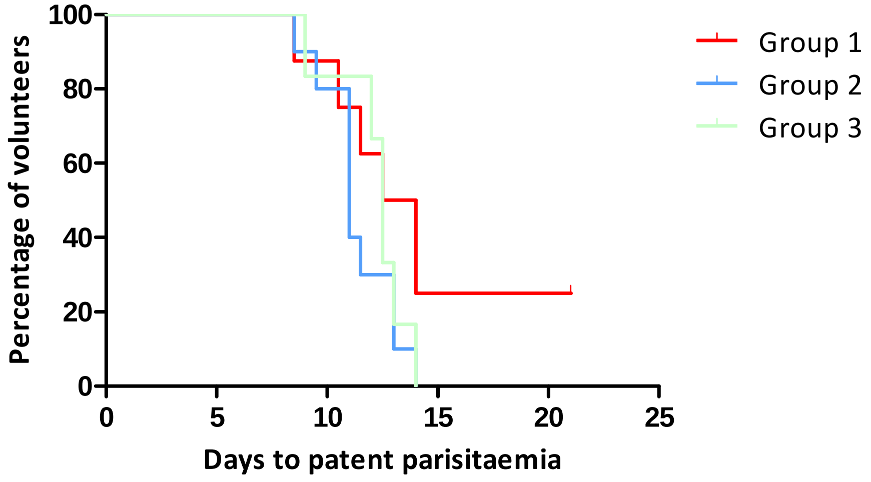

Figure 9 *Kaplan-Meier survival curve of days to patent parasitaemia, demonstrating 25% sterile protection*
Group 1: AdCh63 ME-TRAP 5 x 1010vp boosted with MVA ME-TRAP 2 x 10^8pfu
Group 2: AdCh63 ME-TRAP alone, Group 3: controls

Blood Stage Vaccines

VAC037 (GTAC 166): AdCh63 is currently in use with the MSP1 insert in a phase I/IIa dose escalation clinical trial in Oxford. The trial design includes AdCh63 MSP1 administered alone and with MVA MSP1 as part of a heterologous prime boost schedule with sporozoite challenge of 3 volunteers in the higher dose group to assess the safety of this challenge model following blood-stage vaccination. The route of all vaccinations is intramuscular. The current status of this trial is summarised in Table 6:

Group Number	Vaccination 1	Vaccination 2 (8 wks later)	Sporozoite Challenge	Number of volunteers
**1A**	AdCh63 MSP1 5 x 10^9 vp	-	-	2/2 enrolled
**1B**	AdCh63 MSP1 5 x 10^9 vp	MVA MSP1 5 x 10^8 pfu	-	4/4 enrolled
**2A**	AdCh63 MSP1 5 x 10^{10} vp	-	-	2/2 enrolled
**2B**	AdCh63 MSP1 5 x 10^{10} vp	MVA MSP1 5 x 10^8 pfu	-	5/5 enrolled
**2C**	AdCh63 MSP1 5 x 10^{10} vp	MVA MSP1 5 x 10^8 pfu	3 wks later	3/3 enrolled

Table 6 *Current status of VAC 037 (GTAC 166). 3 volunteers undergo sporozoite challenge.*

Safety Data (Interim Analysis May 2010)

At the most recent interim analysis AdCh63 MSP1 demonstrates an excellent safety profile similar to that of AdCh63 ME-TRAP. 16 volunteers have completed 14 days follow-up post AdCh63 MSP1 (Table 7). The vast majority of local and systemic adverse events (AEs) following AdCh63 MSP1 were mild in severity (97%) and all resolved completely. Overall, 94% of volunteers experienced one or more systemic AEs; these were all mild with the exception of a headache of moderate severity starting on day of vaccination, lasting 2 days reported by one volunteer. 69% of volunteers experienced one or more local injection-site reactions which were all mild in intensity with the exception of one case of moderate pain (post AdCh63 MSP1 5 x 10^{10} vp). This adverse event profile is similar to that observed with AdCh63 ME-TRAP, supporting previous evidence that the adverse event profile of virally vectored vaccines is more related to the viral vector used, than the insert expressed.

Adverse Event	5 x 10^9 vp AdCh63 MSP1 I.M. Number of events (% of volunteers) N = 6			5 x 10^{10} vp AdCh63 MSP1 I.M. Number of events (% of volunteers) N = 10		
	Mild	Moderate	Severe	Mild	Moderate	Severe
**Local pain**	3 (50%)	0	0	6 (50%)	1 (10%)	0
**Local swelling**	2 (33%)	0	0	1 (10%)	0	0
**Local erythema**	4 (66%)	0	0	1 (10%)	0	0
**Local warmth**	0	0	0	3 (30%)	0	0
**Local pruritus**	0	0	0	0	0	0
**Local scaling**	0	0	0	0	0	0
**Local adenopathy**	0	0	0	0	0	0
**Headache**	1 (17%)	0	0	9 (80%)	1 (10%)	0
**Malaise**	2 (33%)	0	0	4 (40%)	0	0
**Myalgia**	2 (33%)	0	0	5 (50%)	0	0
**Fatigue**	1 (17%)	0	0	2 (10%)	0	0
**Feverishness**	1 (17%)	0	0	2 (10%)	0	0
**Fever**	1 (17%)	0	0	0	0	0
**Arthralgia**	0	0	0	2 (20%)	0	0
**Nausea**	1 (17%)	0	0	1 (10%)	0	0
**Coryza &/or sore throat**	1 (17%)	0	0	4 (40%)	0	0
**Other**	3 (33%)*	0	0	2 (20%)**	0	0

Table 7 *All solicited and unsolicited local and systemic adverse events considered possibly, probably or definitely related to AdCh63 MSP1 from VAC037 (Interim analysis). * Hordeleum (onset 1 day post vaccination), diarrhoea and abdominal cramps (onset 3 days post vaccination). **Platelets 132 (noted 30 days post immunization, resolved within 32 days), ALT 69, (noted 21 days post immunization, resolved within 37 days),*

At the most recent interim analysis, 12 volunteers had completed 7 days follow-up post boost with MVA MSP1 (Table 8). 10 of these 12 volunteers had MVA MSP1 administered as one injection. 2 volunteers had the vaccination split as two injections of 2.5 x 10^8 pfu in each deltoid.

100% of volunteers experienced one or more local AE post MVA MSP1. 100% of volunteers described injection site pain, which was moderate or severe in intensity in 75% of volunteers. Other local site reactions were mild in severity with the exception of 2 volunteers who had moderate or severe erythema post vaccination. In both cases the erythema was of delayed onset (3-5 days post vaccination) and between 3 and 8cm distal to, and not including the vaccine site. In

both cases, the volunteers were systemically well. This may represent "non-specific activation of the inflammatory system" post vaccination.[104]

100% of volunteers described one or more systemic AE post MVA MSP1. The majority were mild in severity but 3 volunteers (25%) described severe systemic adverse events resulting in one day of absenteeism from work in all 3 cases. These included rigors, malaise, myalgia, fatigue and feverishness which all developed within 24 hours of vaccination and fully resolved within 5 days.

2 volunteers received 5 x 10^8 pfu of MVA MSP1 but administered as two separate injections of 2.5 x 10^8 pfu in each deltoid in order to assess if split dosing reduced local site pain and systemic reactinogenicity. Both these volunteers experienced severe systemic adverse events and either moderate or severe local pain, and so split dosing of MVA MSP1 was not continued.

MVA MSP1 was more reactogenic than MVA ME-TRAP, however the dose of MVA MSP1 in VAC037 (5 x 10^8 pfu) was more than double that used in VAC033 & MAL034 (2 x 10^8 pfu). The dose of MVA MSP1 to be used in future studies will be 1 - 2.5 x 10^8 pfu and we anticipate that this dose reduction will mean less frequent and severe adverse events will be noted in these trials.

Adverse Event	5 x 10^8 pfu MVA MSP1 I.M.<br>Number of events (% of volunteers)<br>N = 12		
	Mild	Moderate	Severe
**Local pain**	4 (33%)	6 (50%)	3 (25%)
**Local swelling**	3 (25%)	0	0
**Local erythema**	8 (67%)	1 (8%)	1 (8%)
**Local warmth**	5 (42%)	0	0
**Local pruritus**	1 (8%)	0	0
**Local scaling**	0	0	0
**Local adenopathy**	0	0	0
**Diarrhoea**	0	0	0
**Headache**	6 (42%)	3 (25%)	1 (8%)
**Malaise**	6 (50%)	0	3 (25%)
**Myalgia**	3 (25%)	1 (8%)	2 (17%)
**Fatigue**	9 (67%)	0	3 (25%)
**Feverishness**	1 (8%)	1 (8%)	2 (17%)
**Fever**	0	0	1 (8%)
**Arthralgia**	3 (25%)	0	1 (8%)
**Nausea**	3 (25%)	1 (8%)	1 (8%)
**Coryza &/or sore throat**	1 (8%)	0	0
**Other**	6* (50%)	1** (8%)	2*** (17%)

Table 8 *All solicited and unsolicited local and systemic adverse events 7 days considered possibly, probably or definitely related to MVA MSP1 (VAC037 Interim analysis). *Oesophagitis (onset on day of immunization), cervical lymphadenopathy and neck tenderness (onset 9 days post immunization), vasovagal response to venepuncture, tingling of fingers lasting 30 minutes (1 day post immunization) and elevation of ALT to 61 (onset 8 days post immunization lasting 21 days). **Bruising at vaccination site *** Rigors (onset on day of immunization)*

Immunology & Efficacy

Interim analysis of explorative immunology show excellent ex-vivo ELIspot responses with responses post AdCh63 MSP1 and post MVA MSP1 exceeding those previously seen in our group post AdCh63 vectored priming vaccinations (Figure 10). A dose dependant increase in the magnitude of response at both prime and boost was seen.

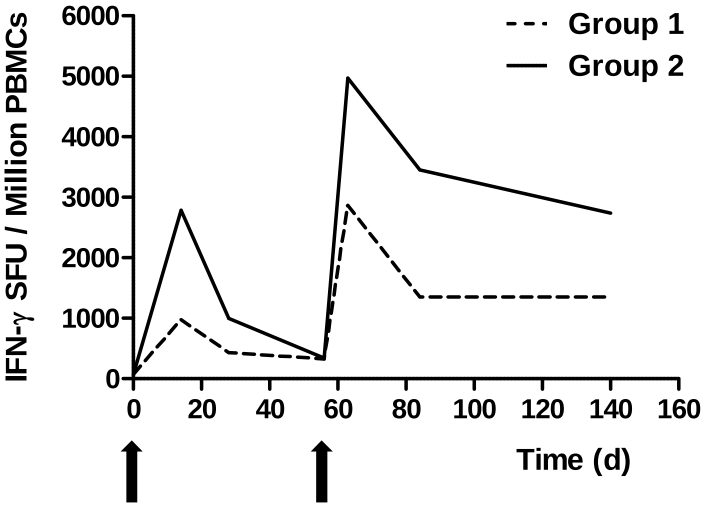

Figure 10a *Median ex-vivo IFN-γ ELISPOT responses to the MSP1 insert (summed response across all the individual peptide pools) are shown over time in PBMCs for each group (analysis includes all applicable data for each group and time-point).*

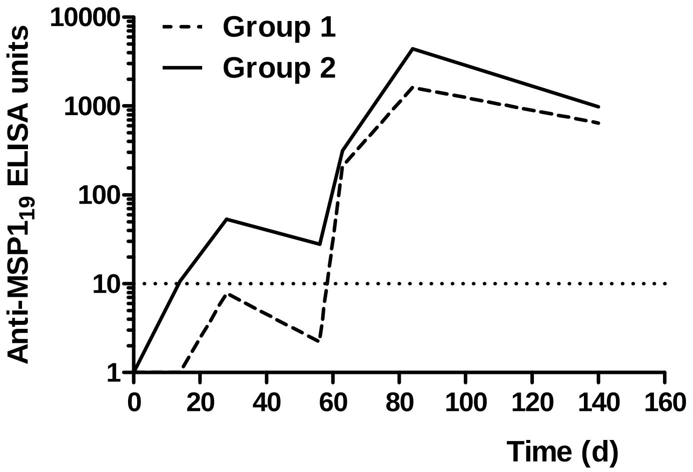

Figure 10b *Total IgG ELISA responses against 3D7 PfMSP1$_{19}$ as measured in the serum over time following AdCh63-MVA MSP1 immunization. The geometric mean response is shown for each group (analysis includes all applicable data for each group and time-point). Individual responses are shown in Supplementary Figure S3. * P = 0.026 by Mann-Whitney U test.*

A delay to patent parasitaemia was seen in all three individuals vaccinated with AdCh63-MVA MSP1 and challenged (*Figure 11*). This is the first demonstration of significant protective efficacy of any vaccine encoding a blood-stage malaria antigen (*Sheehy S et al, unpublished*).

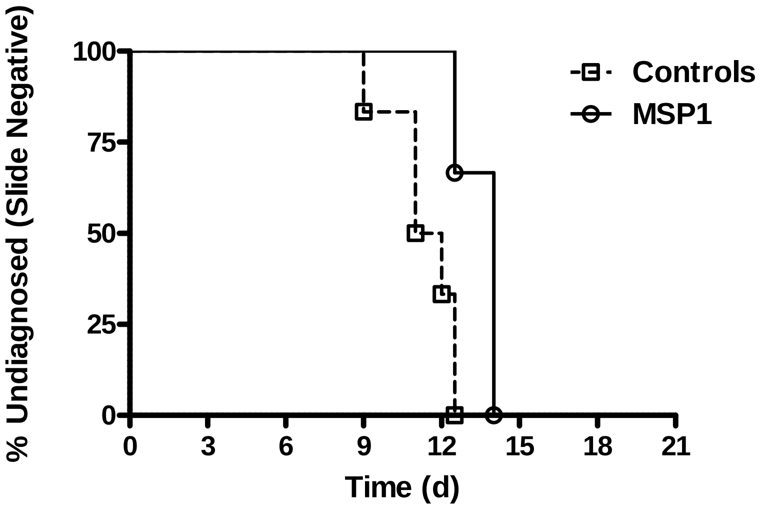

Figure *11* *Kaplan-Meier Survival Analysis of time to patent parsitaemia in days for MSP1 prime-boost vaccinated volunteers (n=3) vs controls (n=6). Median time to patent paristaemia 14 days for vaccineess vs 11.5 days for unvaccinated controls. P=0.03 Log-Rank Test.*

3.8 Study overview

This is an open label phase I study, to assess the safety of two novel malaria vaccines, AdCh63 AMA1, with or without MVA AMA1. This follows promising preclinical studies of AdCh63 AMA1. All volunteers recruited will be healthy adults. Safety data will be collected for each of the regimens. Secondary aims of this study will be to assess the immune responses generated by each of these regimens.

Group Number	Vaccination 1	Vaccination 2 (8 wks later)	Number of volunteers
**1A**	AdCh63 AMA1 5 x 10^9 vp	-	3/4 enrolled
**1B**	AdCh63 AMA1 5 x 10^9 vp	MVA AMA1 2.5 x 10^8 pfu	4/4 enrolled
**2A**	AdCh63 AMA1 5 x 10^{10} vp	-	1/4 enrolled
**2B**	AdCh63 AMA1 5 x 10^{10} vp	MVA AMA1 1.25 x 10^8 pfu	3/4 enrolled

Table 9 *Current status of VAC 036.*

AdCh63 AMA1 We believe that 5 x 10^9 vp of AdCh63 AMA1 is a suitable starting dose for volunteers in this trial. AMA1 has been administered to humans in multiple trials without any evidence of serious safety issues (see above). Also, AdCh63 has been safely administered to 99 individuals at doses up to 2 x 10^{11} vp. We have elected to administer AdCh63 AMA1 intramuscularly given the favourable safety and immunogenicity profile of this route of administration with AdCh63 ME TRAP and AdCh63 MSP-1(Section 3.7).

In group 1, 8 volunteers will receive a single dose of AdCh63 AMA1 at a dose of 5 x 10^9 vp intramuscularly. Only if satisfactory review of safety data and adverse events from this group are provided by the local safety committee, and after a minimum safety interval of two weeks, will we increase the dose of AdCh63 AMA1 to 5 x 10^{10}vp intramuscularly for group 2 (8 volunteers).

Interim safety data: AdCh63 AMA1: June 2010

To date, AdCh63 AMA1 has an excellent safety profile similar to AdCh63 MSP1 & AdCh63 ME-TRAP (Table 10).

**Adverse Event**	**5 x 10^9 vp AdCh63 AMA1 I.M. Number of events (% of volunteers) N = 7**			**5 x 10^{10} vp AdCh63 AMA1 I.M. Number of events (% of volunteers) N = 4**		
	Mild	Moderate	Severe	Mild	Moderate	Severe
**Local pain**	3 (43%)	0	0	6 (100%)	1 (25%)	0
**Local swelling**	0	0	0	1 (25%)	0	0
**Local erythema**	1 (14%)	0	0	3 (75%)	0	0
**Local warmth**	2 (29%)	0	0	0	0	0
**Local pruritus**	0	0	0	0	0	0
**Local scaling**	0	0	0	0	0	0
**Local adenopathy**	0	0	0	0	0	0
**Diarrhoea**	0	0	0	0	0	0
**Headache**	3 (29%)	0	0	3 (75%)	0	0
**Malaise**	1 (14%)	0	0	3 (75%)	0	0
**Myalgia**	2 (29%)	0	0	2 (25%)	0	0
**Fatigue**	2 (29%)	0	0	1 (25%)	0	0
**Feverishness**	0	0	0	3 (50%)	0	0
**Fever**	0	0	0	0	0	0
**Arthralgia**	0	0	0	2 (25%)	0	0
**Nausea**	1 (14%)	0	0	0	0	0
**Coryza/sore throat/cough**	3 (29%)	0	0	1 (25%)	0	0
**Other**	2 (29%)*	0	0	0	0	0

Table 10 *All solicited and unsolicited local and systemic adverse events 14 days considered possibly, probably or definitely related to AdCh63 AMA1 (VAC036 Interim analysis). *Dysmenorrhoea & Abdominal pain*

MVA AMA1 In each group, 4 volunteers will receive a second vaccination with MVA AMA1 intramuscularly 8 weeks later. The MVA vector has been safely utilised in many vaccine

candidates previously and doses up to 5 x 10^8 pfu have been safely administered intramuscularly with reduced local reactogenicity compared to intradermal administration [67]. Preclinically, intramuscular administration of MVA MSP-1 has been shown to induce greater antibody responses in comparison to intradermal administration of the same vaccine (Goodman et al submitted, Douglas et al submitted). However, given the marked reactogenicity seen in VAC037 with 5 x 10^8 pfu of MVA MSP1, we proposed to use the lower dose of 2.5 x 10^8 pfu for MVA AMA1 in this study. To date, 4 volunteers in group 1B have been boosted with 2.5 x 10^8 pfu for MVA AMA1 (Table 11). All volunteers experienced local pain which was moderate in intensity in 3 volunteers (75%), All volunteers experienced systemic adverse events which were severe in intensity in 3 volunteers (75%), resulting in absenteeism from work. Given this marked reactinogenicity profile, the dose of MVA AMA1 will be reduced to 1.25 x 10^8 pfu for the remaining volunteers (group 2B).

**Adverse Event**	**2.5 x 10^8 pfu MVA AMA1 I.M.**<br>**Number of events (% of volunteers)**<br>**N = 4**		
	Mild	Moderate	Severe
**Local pain**	1 (25%)	3 (75%)	0
**Local swelling**	2 (50%)	0	0
**Local erythema**	4 (100%)	0	0
**Local warmth**	2 (50%)	0	0
**Local pruritus**	0	0	0
**Local scaling**	0	0	0
**Local adenopathy**	0	0	0
**Diarrhoea**	0	0	0
**Headache**	3 (75%)	1 (25%)	0
**Malaise**	0	1 (25%)	3 (75%)
**Myalgia**	1 (25%)	1 (25%)	2 (50%)
**Fatigue**	1 (25%)	2 (50%)	2 (50%)
**Feverishness**	2 (50%)	0	1 (25%)
**Fever**	2 (50%)	0	0
**Arthralgia**	0	1 (25%)	1 (25%)
**Other**	2* (25%)	0	3** (75%)

Table 11 *All solicited and unsolicited local and systemic adverse events 7 days considered possibly, probably or definitely related to MVA AMA1 (VAC036 Interim analysis). *Bruising at vaccine site & chills ** 2 cases of rigors and 1 delirium*

Interim Immunogenicity Data

Following priming with AdCh63 AMA1, MVA AMA1 has induced good immunogenicity, with ex-vivo IFN-γ ELISPOT responses to the AMA1 insert of more than 9000 sfu/10^6 PBMC in the volunteers for whom we have preliminary immunogenicity data (Figure 12).

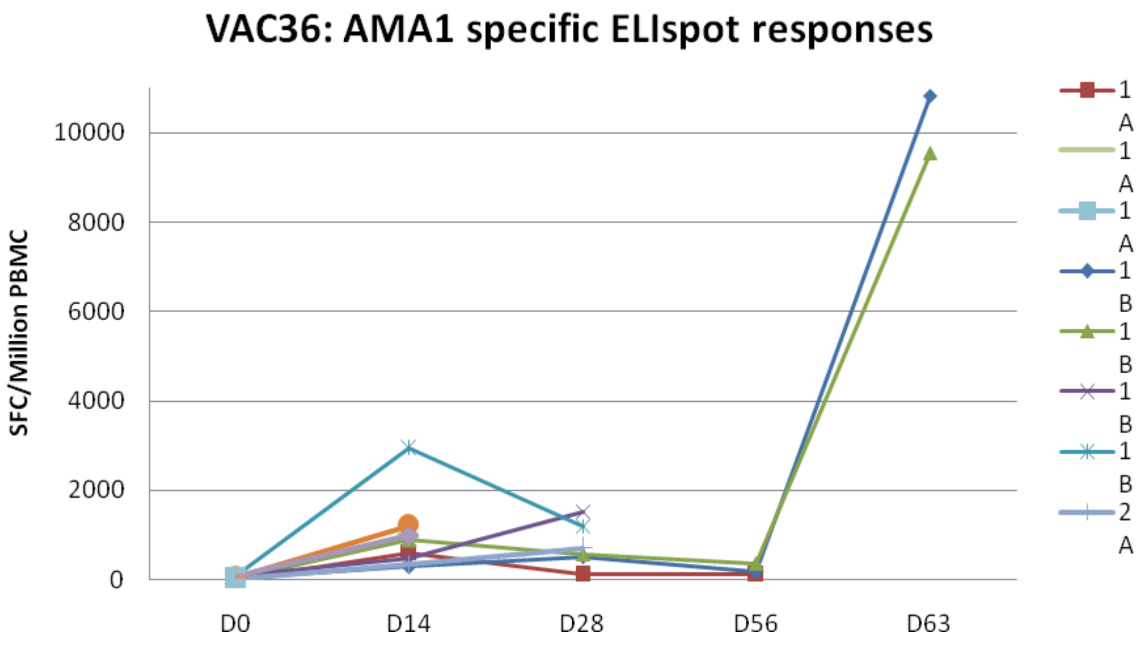

Figure 12 *Interim immunogenicity data: Ex-vivo IFN-γ ELISPOT responses to the AMA1 insert (summed response across all the individual peptide pools) are shown over time in PBMCs for each volunteer*

3.9 Potential Risks

The general risks to participants in this Phase I study are associated with phlebotomy and with vaccination. The volume of blood drawn over the 1 year study period (approximately 400-700 ml), should not compromise these otherwise healthy volunteers. Potential risks include local and systemic reactions, which are described below.

Local reactions

Mild tenderness, bruising, light-headedness or, rarely, syncope, may result from venepuncture. It is possible to predict side effects for AdCh63 AMA1 from the interim safety data, other clinical trials using AdCh63 ME-TRAP and AdCh63 MSP-1 and previous AdHu trials. Adverse events such as pain would be expected to occur frequently. Less frequent adverse events are likely to include erythema, swelling, itching and warmth. Adverse events are likely to be mild in nature and resolve within approximately 48 hours.

The local adverse event profile post intramuscular vaccination with 1.25 x 10^8 pfu MVA AMA1 is likely to be less severe than that seen in volunteers receiving 2.5 x 10^8 pfu and similar to that seen

previously with MVA ME-TRAP. It is expected that the majority of injection site reactions will be of mild severity, resolving within 24 – 72 hours, although there is a possibility of moderate arm pain.,

During the manufacturing process of AdCh63 AMA1 a biocide named Kathon will be used. Kathon is added to body washes, conditioners, liquid soaps, shampoos and wipes as a preservative. The maximum dose is 0.1% for 'rinse off' products and for 'leave on' products it is 0.05%. It has been approved by regulatory authorities throughout the world as a preservative in these products. As a skin sensitiser it is known to cause contact dermatitis. An internal study SOP/P22 was set up to look at the levels of Kathon that were removed during the final purification step of buffer exchange. This study utilized high performance liquid chromatography and showed that trace amounts of Kathon may be left on the column after carrying out the rinse and sanitisation steps. However, the study confirmed greater than 99.9975% removal of Kathon to approximately 30 fold less than the limits for 'leave on' products containing Kathon. We will exclude anyone from the study with a history of clinically significant contact dermatitis or sensitivity to Kathon.

Systemic Reactions

Common systemic adverse events post viral vectored vaccines include headache, feverishness, fatigue, and malaise. Generally volunteers report a transient flu like illness within 24 hours of vaccination which resolves rapidly. Given existing data for AdCh63 vectored vaccines in Oxford, including AdCh63 AMA1, it is anticipated that the majority of systemic adverse events post AdCh63 AMA1 in this trial will be mild in intensity.

The systemic adverse event profile post vaccination with 1.25×10^8 pfu MVA AMA1 is likely to be less severe than that seen in volunteers receiving 2.5×10^8 pfu, and similar to that seen with MVA ME-TRAP previously. It is expected that the majority of systemic adverse events will be of mild severity, resolving within 24 – 48 hours, however, there is a possibility that despite the dose reduction volunteers experience moderate or severe 'flu' like symptoms as seen with 2.5×10^8 pfu MVA AMA1.

As with any other vaccine, the Guillain-Barré syndrome, (GBS) or immune-mediated reactions that can lead to organ damage including serious allergic reactions may occur.

3.10 Known Potential Benefits

Volunteers will not benefit directly from participation in this study. However, it is hoped that the information gained from this study will contribute to the development of a safe and effective malaria vaccine regime. The only benefits for participants would be information about their general health status.

4. OBJECTIVES

4.1 Primary Objectives

To assess the safety in healthy volunteers of two candidate malaria vaccines, AdCh63 AMA1 with MVA AMA1 in a prime-boost regimen. The safety of AdCh63 administered alone, and combined with MVA AMA1 will be assessed. The specific endpoints for safety and reactogenicity will be actively and passively collected data on adverse events.

4.2 Secondary Objectives

To assess the cellular and humoral immune response generated, and whether this is affected by immunity to human adenovirus, by AdCh63 AMA1 when administered individually and sequentially with MVA AMA1 in a prime-boost regime to healthy volunteers.

.

5. INVESTIGATIONAL PRODUCTS

Two investigational vaccines will be used in this study namely and AdCh63 AMA1 and MVA AMA1.

5.1 Vaccine insert, formulation, storage and accountability

The Insert in both the AdCh63 and MVA vaccines is the same and consists of a rationally-designed composite sequence from the *P. falciparum* blood-stage antigen AMA1. The sequence of the AMA1 insert in this vaccine contains from N- to C- terminus: the leader sequence from human tissue plasminogen activator (tPA) followed in-frame by the sequences encoding the ectodomain (amino acids 25-546) of *P. falciparum* (strain 3D7) AMA1 followed by the ectodomain plus C-terminal transmembrane region (amino acids 25-574) of *P. falciparum* (strain FVO) AMA1. These two regions represent two of the most diverged allelic variants of AMA1 (3D7 and FVO), and are separated by a flexible linker sequence (GGGPGGG that we have used safely in another malaria construct in phase I/II trials).

AdCh63 AMA1 will be manufactured under Good Manufacturing Practice conditions by the Clinical Biomanufacturing Facility (CBF), Churchill Hospital, Oxford. AdCh63 AMA1 will be supplied as a liquid in sterile aliquots in glass vials.

MVA AMA1 is manufactured under Good Manufacturing Practice conditions by Impfstoffwerk Dessau-Tornau (IDT), Germany. MVA AMA1 Lot 0010809 is supplied as a liquid formulation in Tris buffer (10 mM Tris, 140 mM NaCl, pH 7.7),at a concentration of 1.24. x 10^9 plaque-forming units (pfu)/mL. The virus suspension is supplied as sterile 0.2 mL aliquots in 2.0 mL clear glass injection vials. The single doses of MVA AMA1 to be used in this study will be 2.5 x 10^8 pfu and 1.25 x 10^8 pfu delivered intramuscularly. Final batch certification and associated labelling takes place at the Clinical Biomanufacturing Facility, Churchill Hospital, Oxford.

The vials will be stored between –65^{0}C and –90^{0}C, in a locked, temperature monitored freezer at the University of Oxford, Churchill Hospital. All movements of the study vaccines between CBF and the University of Oxford and between the locked freezer and clinic room will be documented.

5.2 AdCh63 AMA1 dose

The doses of AdCh63 AMA1 to be used in this study will be 5 x 10^9 and 5 x 10^{10} vp.

AdCh63 AMA1, Lot 01 Fill 09-04 is supplied in glass vials at a concentration of 1.67 x 10 11 VP. mL in a total volume of 0.65 mL.

5.3 MVA AMA1 dose

The dose of MVA AMA1 will be 2.5 x 10^8 pfu or 1.25 x 10^8 pfu administered intramuscularly.

The vaccine concentration is 1.24 x 10^9 pfu/mL.

5.4 Vaccine administration

On vaccination day, vaccines will be allowed to thaw to room temperature and administered within 1 hour. Depending on dosage one or more vials of vaccine may be used. For the 5 x 10^{9} vp dose, vaccine will be drawn up and diluted to obtain a suitable volume for intramuscular injection. The vaccines will be administered intramuscularly over the deltoid region of the arm. The investigator will wear gloves and eye protection. During administration of the vaccines, Advanced Life Support drugs and resuscitation equipment will be immediately available for the management of anaphylaxis.

The first volunteer in groups 1 and 2 will be vaccinated alone and then reviewed 48 hours following vaccination. In the absence of any safety concern, the remaining volunteers in the respective groups will then be vaccinated. There will be a minimum interval of two weeks between the vaccination of volunteers in groups 1 and 2 to allow an independent safety review. Safety data and adverse events will be reviewed by the Local Safety Committee prior to dose escalation in group 2.

.

5.5 Minimising environmental contamination with Genetically Modified Organisms (GMO)

The study will be performed in accordance with Contained Release Regulations (2000). In order to minimise dissemination of the recombinant vectored vaccine virus into the environment, the inoculation site will be covered with a dressing after immunisation. This should absorb any virus that may leak out through the needle track. The dressing will be removed from the injection site after 30 minutes and will be disposed as GMO waste by autoclaving, in accordance with the relevant Standard Operating Procedure (SOP) and current standard UK practice.

6. RECRUITMENT AND WITHDRAWAL OF TRIAL VOLUNTEERS

6.1 Volunteers

Volunteers may be recruited by use of an advertisement formally approved by the ethics committee and distributed or posted in the following places:

- In public places with the agreement of the owner / proprietor
- In newspapers or other literature for circulation
- On a website operated by our group or with the agreement of the owner or operator
- By e-mail distribution to a group or list only with the express agreement of the network administrator or with equivalent authorisation
- On stalls or stands at exhibitions or fairs

6.2 Informed Consent

All volunteers will sign and date the informed consent form before any study specific procedures are performed. The information sheet will be made available to the volunteer at least 24 hours prior to the screening visit. At the screening visit, the volunteer will be fully informed of all aspects of the trial, the potential risks and their obligations. The following general principles will be emphasised:

- Participation in the study is entirely voluntary
- Refusal to participate involves no penalty or loss of medical benefits
- The volunteer may withdraw from the study at any time
- The volunteer is free to ask questions at any time to allow him or her to understand the purpose of the study and the procedures involved
- The study involves research of an investigational vaccine
- There is no direct benefit from participating
- The volunteer's GP will be contacted to corroborate their medical history

The aims of the study and all tests to be carried out will be explained. The volunteer will be given the opportunity to ask about details of the trial, and will then have time to consider whether or not to participate. If they do decide to participate, they will sign and date two copies of the consent form, one for them to take away and keep, and one to be stored in the CRF. These forms will also be signed and dated by the Investigator.

6.3 Inclusion and Exclusion Criteria

This study will be conducted in healthy adults, who meet the following inclusion and exclusion criteria.

Inclusion Criteria

The volunteer must satisfy all the following criteria to be eligible for the study:

- Healthy adults aged 18 to 50 years
- Able and willing (in the Investigator's opinion) to comply with all study requirements
- Willing to allow the investigators to discuss the volunteer's medical history with their General Practitioner
- For females only, willingness to practice continuous effective barrier contraception during the study and a negative pregnancy test on the day(s) of vaccination
- For males only to use barrier contraception until three months after the last vaccination
- Agreement to refrain from blood donation during the course of the study
- Written informed consent

Exclusion Criteria

The volunteer may not enter the study if any of the following apply:

- Participation in another research study involving an investigational product in the 30 days preceding enrolment, or planned use during the study period
- Prior receipt of a recombinant adenoviral vaccine.
- Administration of immunoglobulins and/or any blood products within the three months preceding the planned administration of the vaccine candidate
- Any confirmed or suspected immunosuppressive or immunodeficient state, including HIV infection; asplenia; recurrent, severe infections and chronic (more than 14 days) immunosuppressant medication within the past 6 months (inhaled and topical steroids are allowed)
- History of allergic disease or reactions likely to be exacerbated by any component of the vaccine, e.g. egg products, Kathon.
- History of clinically significant contact dermatitis
- Any history of anaphylaxis in reaction to vaccination
- Pregnancy, lactation or willingness/intention to become pregnant during the study
- History of cancer (except basal cell carcinoma of the skin and cervical carcinoma in situ)
- History of serious psychiatric condition
- Any other serious chronic illness requiring hospital specialist supervision

- Suspected or known current alcohol abuse as defined by an alcohol intake of greater than 42 units every week
- Suspected or known injecting drug abuse
- Seropositive for hepatitis B surface antigen (HBsAg)
- Seropositive for hepatitis C virus (antibodies to HCV)
- Any other significant disease, disorder or finding, which, in the opinion of the Investigator, may either put the volunteer at risk because of participation in the study, or may influence the result of the study, or the volunteer's ability to participate in the study.
- Any history of malaria or
- Travel to a malaria endemic region during the study period or within the previous six months
- Any clinically significant abnormal finding on screening biochemistry or haematology blood tests or urinalysis
- Any other finding which in the opinion of the investigators would significantly increase the risk of having an adverse outcome from participating in the protocol.

  Prevention of 'Over Volunteering'
  Volunteers will be excluded from the study if they are concurrently involved in another trial. In order to check this, volunteers will be asked to provide their National Insurance or Passport number (if they are not entitled to a NI number) and will be registered on a national database of participants in clinical trials. (*www.tops.org.uk*).

Re-vaccination exclusion criteria

The following adverse events associated with vaccine immunisation constitute absolute contraindications to further administration of vaccine. If any of these events occur during the study, the subject must be withdrawn and followed until resolution of the event, as with any adverse event.

- Anaphylactic reaction following administration of vaccine
- Pregnancy

The following adverse events constitute contraindications to administration of vaccine at that point in time; if any one of these adverse events occurs at the time scheduled for vaccination, the subject may be vaccinated at a later date, or withdrawn at the discretion of the investigator. The subject must be followed until resolution of the event as with any adverse event

- Acute disease at the time of vaccination. (Acute disease is defined as the presence of a moderate or severe illness with or without fever.) All vaccines can be administered to persons with a minor illness such as diarrhoea, mild upper respiratory infection with or without low-grade febrile illness, *i.e.*, temperature of <37.5°C/99.5°F).

- Temperature of ≥37.5°C (99.5°F) at the time of vaccination.

6.4 Withdrawal of Volunteers

Volunteers may withdraw or be withdrawn for any of the reasons given below. The reason for withdrawal will be recorded in the Case Report Form (CRF). If withdrawal is due to an adverse event, appropriate follow-up visits or medical care will be arranged until the adverse event has resolved or stabilised. Any volunteer who is withdrawn from the study may be replaced, if that is possible within the specified time frame. The Local Safety Monitor (LSM) may recommend withdrawal of volunteers (see Section 10.6).

Discontinuation Criteria

In accordance with the principles of the current revision of the Declaration of Helsinki (updated 2008) and any other applicable regulations, a volunteer has the right to withdraw from the study at any time and for any reason and is not obliged to give his or her reasons for doing so. The Investigator may withdraw the volunteer at any time in the interests of the volunteer's health and well-being. In addition the volunteer may withdraw/be withdrawn for any of the following reasons:

- Administrative decision by the Investigator
- Ineligibility (either arising during the study or retrospective, having been overlooked at screening)
- Significant protocol deviation
- Volunteer non-compliance with treatment regime or study requirements
- An adverse event which requires discontinuation of the vaccination regimen or results in inability to continue to comply with study procedures (see below)

6.5 Compliance with Dosing Regime

All doses in this vaccine study will be administered by the Investigator and recorded in the CRF. The study medication will be at no time in the possession of the volunteer and compliance will not, therefore, be an issue.

6.6 Pregnancy

Should a volunteer become pregnant during the trial, she will be followed up as other volunteers and in addition will be followed until pregnancy outcome. We will not perform venepuncture as a routine.

7. TRIAL DESIGN

7.1 Primary and Secondary Endpoints

The specific endpoints for safety and reactogenicity will be actively and passively collected data on adverse events. The specific endpoints for immunogenicity will be markers of humoral and cell-mediated immunity.

7.2 Study Groups

This is an open label Phase 1 study of a prime-boost malaria vaccination regimen using one vaccine candidate in healthy volunteers. There will be 2 study groups, each containing 8 volunteers (see table 12). Recruitment of groups will be sequential.

Group Number		Number of volunteers	Dose AdCh63 AMA1	Dose MVA AMA1
**1**	**A**	4	5 x 10^{9} vp IM	--
	**B**	4	5 x 10^{9} vp IM	2.5 x 10^{8} pfu IM
**2**	**A**	4	5 x 10^{10} vp IM	-
	**B**	4	5 x 10^{10} vp IM	1.25 x 10^{8} pfu IM

Table 12: Trial groups

7.3 First Volunteers

The first volunteer in groups 1 and 2 will be vaccinated alone and then reviewed 48 hours following vaccination. In the absence of any safety concern the remaining volunteers in the respective groups will then be vaccinated. There will be a minimum interval of two weeks between the vaccination of the first volunteer in groups 1 and 2 to allow an independent safety review. Safety data and adverse events will be reviewed by the Local Safety Committee prior to dose escalation in group 2.

All volunteers will be issued with the telephone number of the investigators and encouraged to contact the investigators if there are any problems. An investigator will be available 24 hours a day.

7.4 Duration of Study

Groups 1A, and 2A.

These groups will attend for one vaccination, on the day of enrolment. The follow up visits are as described in section 8.2, with a final visit approximately 3 months after enrolment.

Groups 1B and 2B

These groups will attend for two vaccinations, the first on the day of enrolment and the second 8 weeks later (+/- 3 week). The follow up visits are as described in section 8.2, with a final visit approximately 5 months after enrolment.

7.5 Definition of the Start and End of the Trial

The start of the trial is defined as the date of the first vaccination of the first volunteer. The end of the trial is the date of the last visit of the last volunteer.

8. TREATMENT OF TRIAL VOLUNTEERS

8.1 Study procedures

Procedures will be performed on the visit time points indicated in the schedule of procedures. Additional procedures or laboratory tests may be performed, at the discretion of the investigators e.g. urine microscopy in the event of positive urinalysis.

Observations

Observations which will be documented are pulse, blood pressure and temperature.

Blood Tests

Blood will be drawn for the following laboratory tests and processed:

1. At the Oxford Radcliffe Hospitals NHS Trust Laboratories or University College London Hospital NHS Trust Laboratories, using NHS standard procedures:

- **Haematology;** Full Blood Count
- **Biochemistry;** Sodium, Potassium, Urea, Creatinine, Albumin, Liver Function Tests
- **Diagnostic serology;** Adenovirus antibodies**,** HBsAg, HCV antibodies, HIV antibodies (Counselling will be given prior to testing blood for these blood-borne viruses)
- **Immunology;** Human Leukocyte Antigen (HLA) typing

2. At University of Oxford research laboratories:

- **Exploratory Immunology;** *Ex vivo* Elispot assays for interferon gamma and ELISA assays for antibodies to AMA1 will be performed. Other exploratory immunological assays, including ELIspot assays for interleukin-2, flow cytometry assays, antibody assays including ELISA, IFA and *in vitro* growth inhibition assays amongst others, and serum cytokine analysis, may be performed at the discretion of the investigators. These may include gene expression studies as an exploratory analysis.

Urinalysis

Urine will be tested for protein and glucose at screening. For female volunteers only, urine will be tested for beta-human chorionic gonadotrophin (HCG) at screening and immediately prior to each vaccination.

Vaccinations

Before each vaccination, the on-going eligibility of the volunteer will be reviewed. The vaccine will be administered as described above in section 5.4. The injection site will be covered with a sterile dressing and the volunteer will stay in the CCVTM for 2 hours for the first dose cohort and 1 hour

for the second dose cohort, in case of immediate adverse events. Observations will be taken 30 minutes after vaccination and the sterile dressing removed and injection site inspected. An oral thermometer, tape measure and diary card will be given to each volunteer, with instructions on use.

8.2 Study visits

The study visits and procedures will be undertaken by one of the Investigators. The procedures to be included in each visit are documented in the schedule of attendances. Each visit is assigned a time point and a window period, within which the visit will be conducted. These can be found in the schedule of attendances.

Screening Visit

All potential volunteers will have a screening visit, which may take place up to 90 days prior to vaccination. Informed consent will be taken before screening, as described in section 6.1. If consent is obtained, the screening procedures indicated in the schedule of procedures will be undertaken.

Study Visits for group 1A and 2A

Day 0 Enrolment and first vaccination

The eligibility of the volunteer will be reviewed at the end of the screening visit and again when all results from the screening visit have been considered. If eligible, a day 0 visit will be scheduled for the volunteer to receive the first dose of vaccine. Volunteers will not be considered enrolled in the study until they have received their first dose of vaccine. The first vaccine in the first volunteer will be administered at least 48 hours before any subsequent volunteers are vaccinated.

Subsequent visits; day 2 and days 14, 28, 56 90.

On subsequent visits, the volunteers will be assessed for local and systemic adverse events, using a diary card, interim history, physical examination and blood tests at the time points indicated in the schedule of attendances. Blood will also be taken for exploratory immunology analysis.

Timeline of visits

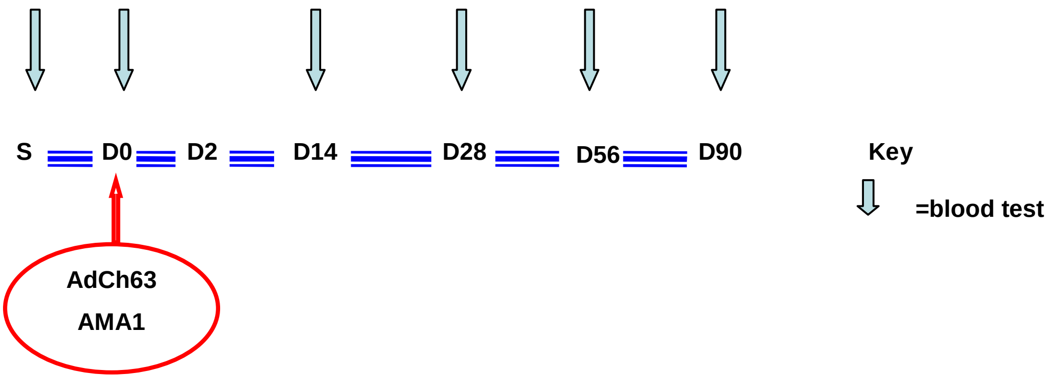

	S	V					
**Attendance number**	**1**	**2**	**3**	**4**	**5**	**6**	**7**
**Timeline (days)**		0	0+2	14	28	56	90
**Window (days)**			±1	±2	±7	±14	±14
**Inclusion / Exclusion criteria**	X						
**Informed consent**	X						
**Medical History**	X	(x)	(x)	(x)	(x)	(x)	(x)
**Physical Examination**	X	(x)	(x)	(x)	(x)	(x)	(x)
**Urinalysis**	X						
**Review contraindications**	X	X					
**Vaccination**		X					
**Observations**	X	X	X	X	X	X	X
**Local & systemic events / reactions**		X	X	X	X	X	X
**Diary cards provided**		X					
**Diary cards collected**				X			
**HLA typing (mL)**		4					
**HBV,HCV,HIV (mL)**	5						
**Anti adenovirus antibodies (mL)**		5					
**Haematology (mL)**	2			2	2	2	2
**Biochemistry (mL)**	4			4	4	4	4
**Exploratory immunology**		70		70	70	70	70
**Blood volume per visit (mL)**	11	79		76	76	76	76
**Cumulative blood volume (mL)**	11	90	90	166	242	318	394

Table 13: Schedule of attendances for group 1A and 2A

S screening visit

V vaccination visit

(x) If considered necessary, emphasising any acute complaints

Visits for groups 1B and 2B (AdCh63 AMA1 and MVA AMA1)

Day 0; Enrolment and first vaccination

The eligibility of the volunteer will be reviewed at the end of the screening visit and again when all results from the screening visit have been considered. If eligible, a day 0 visit will be scheduled for the volunteer to receive the first dose of vaccine. Volunteers will not be considered enrolled in the study until they have received their first dose of vaccine. The vaccine will be administered as described in section 5.4.

Subsequent visits (days 2, 14, 28)

On subsequent visits, the volunteers will be assessed for local and systemic adverse events, using a diary card, interim history, physical examination and blood tests at the time points indicated in the schedule of attendances. Blood will also be taken for exploratory immunology analysis.

Day 56 – Vaccination with MVA AMA1

This visit will include a follow up visit for the first vaccination and administration of the second vaccine. The eligibility of the volunteer will be reviewed prior to vaccination and the vaccine administered as described in section 5.4. The vaccine will be administered intramuscularly for all groups in the opposite arm to that used for the first vaccination.

Subsequent visits (days 58, 63, 84, 140)

On subsequent visits, the volunteers will be assessed for local and systemic adverse events, using a diary card, interim history, physical examination and blood tests as detailed in the schedule of attendances. Blood will also be taken for exploratory immunology analysis.

Timeline of visits groups 1B and 2B

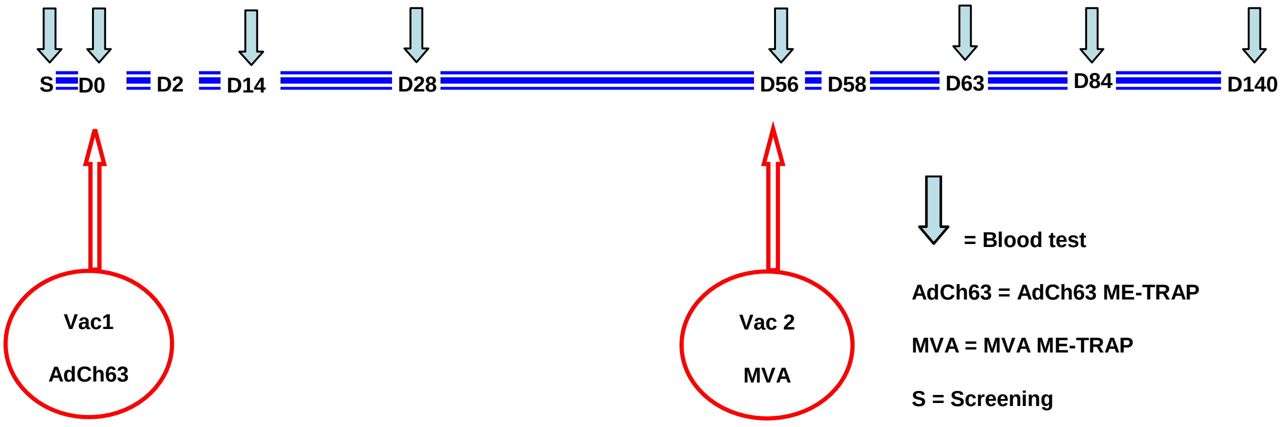

	S	V				V				
**Attendance number**	**1**	**2**	**3**	**4**	**5**	**6**	**7**	**8**	**9**	**10**
**Timeline (days)**		0	0+2	14	28	56	58	63	84	140
**Window (days)**			±1	±2	±7	±7	±1	±2	±7	±14
**Inclusion / Exclusion criteria**	X	(X)				X				
**Informed consent**	X									
**Medical History**	X	(x)	(x)	(x)	(x)	(x)	(x)	(x)	(x)	(x)
**Physical Examination**	X	(x)	(x)	(x)	(x)	(x)	(x)	(x)	(x)	(x)
**Urinalysis**	X									
**B-HCG urine test**	X	X				X				
**Review contraindications**	X	X				X				
**Vaccination**		X				X				
**Vital signs**	X	X	X	X	X	X	X	X	X	X
**Local & systemic events / reactions**		X	X	X	X	X	X	X	X	X
**Diary cards provided**		X				X				
**Diary cards collected**				X				X		
**HLA typing (mL)**		4								
**HBV,HCV,HIV (mL)**	5									
**Anti adenovirus antibodies**		5								
**Haematology (mL)**	2			2	2	2		2	2	2
**Biochemistry (mL)**	4			4	4	4		4	4	4
**Exploratory immunology**		70		70	70	70		70	70	70
**Blood volume per visit (mL)**	11	79		76	76	76		76	76	76
**Cumulative blood volume (mL)**	11	90	90	166	242	318	394	470	546	622

Table 14: Schedule of attendances for Groups 1B and 2B

S screening visit

V vaccination visit

(x) If considered necessary, emphasising any acute complaints

Trial Sites

Screening and follow-up visits will take place at both Oxford (Centre for Clinical Vaccinology and Tropical Medicine) and London sites (University College London, Clinical Research Facility). All vaccinations will take place only at the Oxford site (Centre for Clinical Vaccinology and Tropical Medicine).

9. ASSESSMENT OF SCIENTIFIC OBJECTIVES

9.1 Primary Outcome Measures

To assess the safety in healthy volunteers of two candidate malaria vaccines, AdCh63 AMA1 with MVA AMA1 in a prime-boost regimen. The safety of AdCh63 administered alone, and combined with MVA AMA1 will be assessed. The specific endpoints for safety and reactogenicity will be actively and passively collected data on adverse events.

9.2 Secondary Outcome Measures

To assess the immunogenicity of AdCh63 AMA1, when administered as a single vaccination and sequentially with MVA AMA1 in a prime-boost regimen in healthy volunteers.

10. ASSESSMENT OF SAFETY

Safety will be assessed by the frequency, incidence and nature of adverse events and serious adverse events arising during the study.

10.1 Interim Safety Review

After each dose interval we will have a safety review. This will be conducted by the external Local Safety Committee, and consist of a review of all safety data and adverse effects in volunteers before proceeding to the next dose interval.

10.2 Definitions

Adverse Event (AE)

An AE is any untoward medical occurrence in a volunteer, including a dosing error, which may occur during or after study vaccination and does not necessarily have to have a causal relationship with vaccination. An AE can therefore be any unfavourable and unintended sign (including an abnormal laboratory finding), symptom or disease temporally associated with study vaccination, whether or not considered related to study vaccination.

Adverse Drug Reaction (ADR)

An ADR is any untoward or unintended response to a medicinal product. This means that a causal relationship between the study medication and an AE is at least a reasonable possibility, i.e., the relationship cannot be ruled out.

Unexpected Adverse Reaction

An unexpected adverse reaction is where the nature or severity is not consistent with the Investigator's Brochure.

Serious Adverse Event (SAE) or Adverse Drug Reaction

An SAE is an AE that results in any of the following outcomes, whether or not considered related to the vaccine.

- Death (i.e., results in death from any cause at any time)
- Life-threatening event (i.e., the volunteer was, in the view of the investigator, at immediate risk of death from the event that occurred). This does not include an AE that, if it occurred in a more serious form, might have caused death.
- Persistent or significant disability or incapacity (i.e. substantial disruption of one's ability to carry out normal life functions).

- Hospitalisation, regardless of length of stay, even if it is a precautionary measure for continued observation. Hospitalisation (including inpatient or outpatient hospitalization for an elective procedure) for a pre-existing condition that has not worsened unexpectedly does not constitute a serious AE.
- An important medical event (that may not cause death, be life threatening, or require hospitalization) that may, based upon appropriate medical judgment, jeopardize the volunteer and/or require medical or surgical intervention to prevent one of the outcomes listed above. Examples of such medical events include allergic reaction requiring intensive treatment in an emergency room or clinic, blood dyscrasias, or convulsions that do not result in inpatient hospitalization.
- Congenital anomaly or birth defect.

Suspected Unexpected Serious Adverse Reactions (SUSARs)

A SUSAR is different from an SAE in that it is unexpected and thought to be related to the investigational product. Reports of any SUSAR will be sent to the MHRA and GTAC within 7 for fatal and life-threatening cases and within 15 days for all other SUSARs. Administration of further vaccines within the trial will be suspended until a safety review is convened.

Expected Adverse Drug Reactions

Expected local reactions to the vaccine will be recorded as AEs. These include redness, swelling, scaling, tenderness, or itching.

Expected Serious Adverse Events

No serious adverse events are expected in this study.

10.3 Causality Assessment

For every AE, an assessment of the relationship of the event to the administration of the vaccine will be undertaken. An intervention-related AE refers to an AE for which there is a probable or definite relationship to administration of a vaccine. An interpretation of the causal relationship of the intervention to the AE in question will be made, based on the type of event; the relationship of the event to the time of vaccine administration; and the known biology of the vaccine therapy (table 15).

Table 15: Guidelines for assessing the relationship of vaccine administration to an AE

0	**No Relationship**	No temporal relationship to study product ***and***<br>Alternate aetiology (clinical state, environmental or other interventions); ***and***<br>Does not follow known pattern of response to study product
1	**Possible**	Reasonable temporal relationship to study product; ***or***<br>Event not readily produced by clinical state, environmental or other interventions; ***or***<br>Similar pattern of response to that seen with other vaccines
2	**Probable**	Reasonable temporal relationship to study product; ***and***<br>Event not readily produced by clinical state, environment, or other interventions ***or***<br>Known pattern of response seen with other vaccines
3	**Definite**	Reasonable temporal relationship to study product; ***and***<br>Event not readily produced by clinical state, environment, or other interventions; ***and***<br>Known pattern of response seen with other vaccines

10.4 Reporting Procedures for All Adverse Events

All AEs occurring during the study observed by the investigator or reported by the patient, whether or not attributed to study medication, will be reported in the CRF. All AEs that result in a patient's withdrawal from the study or that are present at the end of the study, will be followed up until a satisfactory resolution occurs, or until a non-study related causality is assigned. All deaths occurring during the study will be reported to the Sponsor. For all deaths, available autopsy reports and relevant medical reports will be made available for reporting to the relevant authorities. The severity of clinical and laboratory adverse events will be assessed according to the scales in Table 16, 17 and 18.

Table 16: Severity grading criteria for adverse events

Adverse Event	Grade	Intensity
Pain at injection site	1	Pain that is easily tolerated
	2	Pain that interferes with daily activity
	3	Pain that prevents daily activity
Erythema at injection site	1	>0 - 50 mm
	2	>50 - 100 mm
	3	>100 mm
Swelling at injection site	1	>0 - 50 mm
	2	>50 - 100 mm
	3	>100 mm
Fever (oral)	1	37.6°C - 38.0°C
	2	>38.0°C – 39.0°C
	3	>39.0°C
Headache	1	Headache that is easily tolerated
	2	Headache that interferes with daily activity
	3	Headache that prevents daily activity
Nausea	1	Nausea that is easily tolerated
	2	Nausea that interferes with daily activity
	3	Nausea that prevents daily activity
Malaise	1	Malaise that is easily tolerated
	2	Malaise that interferes with daily activity
	3	Malaise that prevents daily activity
Myalgia	1	Myalgia that is easily tolerated
	2	Myalgia that interferes with daily activity
	3	Myalgia that prevents daily activity
Arthralgia	1	Joint pain that is easily tolerated
	2	Joint pain that interferes with daily activity
	3	Joint pain that prevents daily activity
Urticaria	1	Requiring no medications
	2	Requiring oral or topical treatment or IV medication or steroids for <24 hours
	3	Requiring IV medication or steroids for >24 hours

Table 17: Severity grading criteria for laboratory abnormalities

Laboratory Test	Grade 1	Grade 2	Grade 3	Grade 4 (Life threatening)
Hgb (female) – decrease from testing laboratory LLN in gm/dl	1.0 - <1.5	≥1.5 & <2.0	≥2.0	Requires transfusion
Hgb (male) – decrease from testing laboratory LLN in gm/dl	≥1.5 & <2.0	≥2.0 & <2.5	≥2.5	Requires transfusion
Absolute neutrophil count (ANC, cells/mm^3)	1000-1499	500-999	<500	<500 with fever
Leukopenia (WBC, cells/mm^3)	<3500 - ≥2500	<2500 - ≥1500	<1500	<1500 with fever
Platelets (cells/mm^3)	125,000 – 135,000	100,000 – 124,000	20,000-99,000	<20,000
Bilirubin – when accompanied by any increase in Liver Function Test increase by factor	1.1 – 1.25 x ULN	1.26 – 1.5 x ULN	1.51 – 1.75 x ULN	> 1.75 x ULN
ALT	1.25 – 2.5 x ULN	>2.6 – 5.0 x ULN	>5.0 x ULN	>10 x ULN and requires hospitalization
Creatinine	1.1 – 1.5 x ULN	>1.6 – 3.0 x ULN	>3.0 x ULN	>5.0 x ULN and requires dialysis
Urine protein	2+ or 0.5-1 gm loss/day	3+ or 1-2 gm loss/day	4+ or >2 gm loss/day	NA
Hematuria	2+ confirmed by 5-10 rbc/hpf	3+ confirmed by >10 rbc/hpf	gross, with or without clots, OR red blood cell casts	NA

Table 18: Functional scale for assessing the severity of AEs

Scale	Description	Definition
**0**		Absence of the indicated symptom
**1**	Mild	Awareness of a symptom but the symptom is easily tolerated
**2**	Moderate	Discomfort enough to cause interference with usual activity
**3**	Severe	Incapacitating; unable to perform usual activities; requires absenteeism or bed rest
**4**	Serious	Life-threatening

10.5

10.6 Reporting Procedures for Serious Adverse Events

In order to comply with current regulations on serious adverse event reporting to Health Authorities, the event will be documented accurately and notification deadlines respected. SAEs will be reported to an internal safety group, within 1 working day of the investigators being aware of their occurrence, as described in internal SOP-VC009. SAEs will not normally be reported to GTAC, unless there is a clinically important increase in occurrence rate, an unexpected outcome, or a new event that is likely to affect safety of trial volunteers. In addition to the expedited reporting above, the investigator shall submit once a year throughout the study or, on request, a safety report to the Competent Authority and Ethics Committee.

10.7 Procedures to be followed in the event of abnormal findings

Abnormal clinical findings from medical history, examination or blood tests, will be assessed as to their clinical significance using the table in Appendix A. If a test is deemed clinically significant, it may be repeated, to ensure it is not a single occurrence. If a test remains clinically significant, the volunteer will be informed and appropriate medical care arranged as appropriate and with the permission of the volunteer. Decisions to exclude the volunteer from the enrolling in the trial or to withdraw a volunteer from the trial will be at the discretion of the Investigator, following procedures for adverse events as described in section 10.1.

10.8 Local Safety Committee

A Local Safety Committee (LSC) will be appointed to provide real-time safety oversight. The LSC will review SAEs if deemed possibly, probably or definitely related to vaccination. The LSC will be notified within 1 working day of the investigators' being aware of their occurrence. The LSC has the power to terminate the study if deemed necessary following a vaccine-related SAE. The LSC will be

chaired by Dr Brian Angus, a Clinical Tutor in Medicine, Honorary Consultant Physician and Director, Centre for Tropical Medicine at the University of Oxford. There will be 2 other Consultant Physicians on the panel, as yet to be identified.

10.9 Safety Profile Review

The safety profile will be assessed on an ongoing basis by the investigators, and specifically after group one has been vaccinated, before enrolling volunteers into group two. The Local Safety Committee will perform an independent external safety review prior to dose escalation in group 2. An internal safety group will also review safety issues and SAEs as they arise.

11. STATISTICS

This is an observational and descriptive safety study, where 2 groups of 8 volunteers will be vaccinated with AdCh63 AMA1 alone or in combination with MVA AMA1 i.e. 16 volunteers will be vaccinated in total. This sample size should allow determination of the magnitude of the outcome measures, especially of serious and severe adverse events, rather than aiming to obtain statistical significance.

12. QUALITY CONTROL AND QUALITY ASSURANCE PROCEDURES

Data will be evaluated for compliance with the protocol and accuracy in relation to source documents. The study will be conducted in accordance with procedures identified in the protocol. SOPs will be used at all clinical and laboratory sites. Regular monitoring will be performed according to ICH Good Clinical Practice (GCP). Following written standard operating procedures, the monitors will verify that the clinical trial is conducted and data are generated, documented and reported in compliance with the protocol, GCP and the applicable regulatory requirements. The investigator site will provide direct access to all trial related source data/documents and reports for the purpose of monitoring and auditing by the sponsor, and inspection by local and regulatory authorities.

13. ETHICS

13.1 Declaration of Helsinki

The Investigator will ensure that this study is conducted according to the principles of the current revision of the Declaration of Helsinki 2008.

13.2 ICH Guidelines for Good Clinical Practice

The Investigator will ensure that this study is conducted in full conformity with relevant regulations and with the ICH guidelines for GCP (CPMP/ICH/135/95) July 1996.

13.3 Informed Consent

Written, informed consent will be obtained, as described in section 6.1.

13.4 Independent Ethics Committee (IEC)

A copy of the protocol, proposed informed consent form, other written volunteer information and the proposed advertising material will be submitted to an IEC (GTAC) for written approval. The Investigator will submit and, where necessary, obtain approval from the GTAC for all subsequent substantial amendments to the protocol and informed consent document. The Investigator will notify deviations from the protocol or SAEs occurring at the site to the sponsor and will notify GTAC of these in accordance with local procedures.

13.5 Volunteer Confidentiality

All data will be anonymised: volunteer data will be identified by a unique study number in CRF and database. A separate confidential file containing identifiable information will be stored in a secured location in accordance with the Data Protection Act 1998. Only the sponsor representative, investigators, the clinical monitor, the GTAC and the MHRA will have access to the records. Photographs taken of vaccination sites (if required, with the volunteer's written, informed consent) will not include the volunteer's face and will be identified by the date, trial code and subject's unique identifier. Once developed, photographs will be stored as confidential records, as above. This material may be shown to other professional staff, used for educational purposes, or included in a scientific publication.

14. DATA HANDLING AND RECORD KEEPING

14.1 Data Handling

The Chief Investigator will be the data manager with responsibility for receiving, entering, cleaning, querying, analysing and storing all data that accrues from the study. The data will be entered into the volunteers' CRFs in a paper and/or electronic format (using OpenClinica™ database stored on a secure University of Oxford server - Data will be entered in a web browser on PCs in the CCVTM building and then transferred to the OpenClinica Database by encrypted (Https) transfer. OpenClinica™ meets FDA part 11B standards).. This includes safety data, laboratory data (both clinical and immunological) and outcome data.

14.2 Record Keeping

The investigators will maintain appropriate medical and research records for this trial, in compliance with ICH E6 GCP and regulatory and institutional requirements for the protection of confidentiality of volunteers. The Chief Investigator, co-investigators and clinical research nurses will have access to records. The investigators will permit authorized representatives of the sponsor(s), and regulatory agencies to examine (and when required by applicable law, to copy) clinical records for the purposes of quality assurance reviews, audits and evaluation of the study safety and progress.

14.3 Source Data and Case Report Forms (CRFs)

All protocol-required information will be collected in CRFs designed by the investigator. All source documents will be filed in the CRF. Source documents are original documents, data, and records from which the volunteer's CRF data are obtained. For this study, these will include, but are not limited to, volunteer consent form, blood results, GP response letters, laboratory records, diaries, and correspondence. In the majority of cases, CRF entries will be considered source data as the CRF is the site of the original recording (i.e., there is no other written or electronic record of data). In this study this will include, but is not limited to medical history, medication records, vital signs, physical examination records, urine assessments, blood results, adverse event data and details of vaccinations. All source data and volunteer CRFs will be stored securely.

14.4 Data Protection

The study protocol, documentation, data and all other information generated will be held in strict confidence. No information concerning the study or the data will be released to any unauthorized third party, without prior written approval of the sponsor.

15. FINANCING AND INSURANCE

15.1 Financing

The study will be funded by research grants from the Wellcome Trust, the Department of Health, the European Commission, and the Medical Research Council, held or to be held by Professor Adrian Hill.

15.2 Insurance

Indemnity and/or compensation for harm arising specifically from an accidental injury, and occurring as a consequence of the Research Subject's participation in the Trial for which the University of Oxford is the Research Sponsor will be covered by the University of Oxford.

15.3 Compensation

Volunteers will be compensated for their time and for the inconvenience caused by procedures, as per the table below which contains approximate amounts.

- Travel expenses	£20 per visit for Oxford volunteers<br>Cost of travel for London volunteers
- Inconvenience of blood tests:	£ 6 per blood donation
- Time required for visit:	£5.50 per hour

Volunteers in group B will receive an additional £100 retention bonus per month for every month they are involved in the study after their second vaccination (in view of the extra time and inconvenience).

.

Group Number	Compensation Amount (approximate)
1A	*£265.50*
1B	*£445.00*
2A	*£232.50*
2B	*£445.00*

REFERENCES

1. Snow, R.W., et al., *The global distribution of clinical episodes of Plasmodium falciparum malaria.* Nature, 2005. **434**(7030): p. 214-7.
2. Sachs, J. and P. Malaney, *The economic and social burden of malaria.* Nature, 2002. **415**(6872): p. 680-5.
3. White, N.J., *Antimalarial drug resistance.* J Clin Invest, 2004. **113**(8): p. 1084-92.
4. Mahanty, S., A. Saul, and L.H. Miller, *Progress in the development of recombinant and synthetic blood-stage malaria vaccines.* J Exp Biol, 2003. **206**(Pt 21): p. 3781-8.
5. Kumar, S., J.E. Epstein, and T.L. Richie, *Vaccines against asexual stage malaria parasites.* Chem Immunol, 2002. **80**: p. 262-86.
6. von der Weid, T., N. Honarvar, and J. Langhorne, *Gene-targeted mice lacking B cells are unable to eliminate a blood stage malaria infection.* J Immunol, 1996. **156**(7): p. 2510-6.
7. Bull, P.C., et al., *Parasite antigens on the infected red cell surface are targets for naturally acquired immunity to malaria.* Nat Med, 1998. **4**(3): p. 358-60.
8. Bull, P.C. and K. Marsh, *The role of antibodies to Plasmodium falciparum-infected-erythrocyte surface antigens in naturally acquired immunity to malaria.* Trends Microbiol, 2002. **10**(2): p. 55-8.
9. Bouharoun-Tayoun, H., et al., *Antibodies that protect humans against Plasmodium falciparum blood stages do not on their own inhibit parasite growth and invasion in vitro, but act in cooperation with monocytes.* J Exp Med, 1990. **172**(6): p. 1633-41.
10. Cohen, S., G.I. Mc, and S. Carrington, *Gamma-globulin and acquired immunity to human malaria.* Nature, 1961. **192**: p. 733-7.
11. McGregor, I.A., *The Passive Transfer of Human Malarial Immunity.* Am J Trop Med Hyg, 1964. **13**: p. SUPPL 237-9.
12. Good, M.F., et al., *Development and regulation of cell-mediated immune responses to the blood stages of malaria: implications for vaccine research.* Annu Rev Immunol, 2005. **23**: p. 69-99.
13. Draper, S.J., et al., *Recombinant viral vaccines expressing merozoite surface protein-1 induce antibody- and T cell-mediated multistage protection against malaria.* Cell Host Microbe, 2009. **5**(1): p. 95-105.
14. Draper, S.J., et al., *Effective induction of high-titer antibodies by viral vector vaccines.* Nat Med, 2008. **14**(8): p. 819-21.
15. Narum, D.L. and A.W. Thomas, *Differential localization of full-length and processed forms of PF83/AMA1 an apical membrane antigen of Plasmodium falciparum merozoites.* Mol Biochem Parasitol, 1994. **67**(1): p. 59-68.
16. Crewther, P.E., et al., *Plasmodium falciparum: two antigens of similar size are located in different compartments of the rhoptry.* Exp Parasitol, 1990. **70**(2): p. 193-206.
17. Waters, A.P., et al., *A merozoite receptor protein from Plasmodium knowlesi is highly conserved and distributed throughout Plasmodium.* J Biol Chem, 1990. **265**(29): p. 17974-9.
18. Polley, S.D., et al., *Human antibodies to recombinant protein constructs of Plasmodium falciparum Apical Membrane Antigen 1 (AMA1) and their associations with protection from malaria.* Vaccine, 2004. **23**(5): p. 718-28.
19. Pinder, M., et al., *Immunoglobulin G antibodies to merozoite surface antigens are associated with recovery from chloroquine-resistant Plasmodium falciparum in Gambian children.* Infect Immun, 2006. **74**(5): p. 2887-93.

20. Narum, D.L., et al., *Immunization with parasite-derived apical membrane antigen 1 or passive immunization with a specific monoclonal antibody protects BALB/c mice against lethal Plasmodium yoelii yoelii YM blood-stage infection.* Infect Immun, 2000. **68**(5): p. 2899-906.

21. Anders, R.F., et al., *Immunisation with recombinant AMA1 protects mice against infection with Plasmodium chabaudi.* Vaccine, 1998. **16**(2-3): p. 240-7.

22. Stowers, A.W., et al., *Vaccination of monkeys with recombinant Plasmodium falciparum apical membrane antigen 1 confers protection against blood-stage malaria.* Infect Immun, 2002. **70**(12): p. 6961-7.

23. Mueller, M.S., et al., *Induction of parasite growth-inhibitory antibodies by a virosomal formulation of a peptidomimetic of loop I from domain III of Plasmodium falciparum apical membrane antigen 1.* Infect Immun, 2003. **71**(8): p. 4749-58.

24. Miao, J., et al., *Immune responses in mice induced by prime-boost schemes of the Plasmodium falciparum apical membrane antigen 1 (PfAMA1)-based DNA, protein and recombinant modified vaccinia Ankara vaccines.* Vaccine, 2006. **24**(37-39): p. 6187-98.

25. Rogers, W.O., et al., *Protection of rhesus macaques against lethal Plasmodium knowlesi malaria by a heterologous DNA priming and poxvirus boosting immunization regimen.* Infect Immun, 2002. **70**(8): p. 4329-35.

26. Silvie, O., et al., *A role for apical membrane antigen 1 during invasion of hepatocytes by Plasmodium falciparum sporozoites.* J Biol Chem, 2004. **279**(10): p. 9490-6.

27. Polley, S.D. and D.J. Conway, *Strong diversifying selection on domains of the Plasmodium falciparum apical membrane antigen 1 gene.* Genetics, 2001. **158**(4): p. 1505-12.

28. Bett, A.J., et al., *An efficient and flexible system for construction of adenovirus vectors with insertions or deletions in early regions 1 and 3.* Proc Natl Acad Sci U S A, 1994. **91**(19): p. 8802-6.

29. Top, F.H., Jr., et al., *Immunization with live types 7 and 4 adenovirus vaccines. I. Safety, infectivity, antigenicity, and potency of adenovirus type 7 vaccine in humans.* J Infect Dis, 1971. **124**(2): p. 148-54.

30. Robbins, P.D. and S.C. Ghivizzani, *Viral vectors for gene therapy.* Pharmacol Ther, 1998. **80**(1): p. 35-47.

31. Lee, T.W., D.A. Matthews, and G.E. Blair, *Novel molecular approaches to cystic fibrosis gene therapy.* Biochem J, 2005. **387**(Pt 1): p. 1-15.

32. Yang, Y., Q. Su, and J.M. Wilson, *Role of viral antigens in destructive cellular immune responses to adenovirus vector-transduced cells in mouse lungs.* J Virol, 1996. **70**(10): p. 7209-12.

33. Raper, S.E., et al., *A pilot study of in vivo liver-directed gene transfer with an adenoviral vector in partial ornithine transcarbamylase deficiency.* Hum Gene Ther, 2002. **13**(1): p. 163-75.

34. Raper, S.E., et al., *Fatal systemic inflammatory response syndrome in a ornithine transcarbamylase deficient patient following adenoviral gene transfer.* Mol Genet Metab, 2003. **80**(1-2): p. 148-58.

35. Catanzaro, A.T., et al., *Phase 1 safety and immunogenicity evaluation of a multiclade HIV-1 candidate vaccine delivered by a replication-defective recombinant adenovirus vector.* J Infect Dis, 2006. **194**(12): p. 1638-49.

36. Rodrigues, E.G., et al., *Efficient induction of protective anti-malaria immunity by recombinant adenovirus.* Vaccine, 1998. **16**(19): p. 1812-7.

37. Maeda, K., et al., *Recombinant adenovirus vector vaccine induces stronger cytotoxic T-cell responses than recombinant vaccinia virus vector, plasmid DNA, or a combination of these.* Viral Immunol, 2005. **18**(4): p. 657-67.

38. Casimiro, D.R., et al., *Heterologous human immunodeficiency virus type 1 priming-boosting immunization strategies involving replication-defective adenovirus and poxvirus vaccine vectors.* J Virol, 2004. **78**(20): p. 11434-8.

39. Tatsis, N. and H.C. Ertl, *Adenoviruses as vaccine vectors.* Mol Ther, 2004. **10**(4): p. 616-29.

40. Kobinger, G.P., et al., *Chimpanzee adenovirus vaccine protects against Zaire Ebola virus.* Virology, 2006. **346**(2): p. 394-401.

41. Casimiro, D.R., et al., *Comparative immunogenicity in rhesus monkeys of DNA plasmid, recombinant vaccinia virus, and replication-defective adenovirus vectors expressing a human immunodeficiency virus type 1 gag gene.* J Virol, 2003. **77**(11): p. 6305-13.

42. Xiang, Z., et al., *Novel, chimpanzee serotype 68-based adenoviral vaccine carrier for induction of antibodies to a transgene product.* J Virol, 2002. **76**(6): p. 2667-75.

43. Grabenstein, J.D., et al., *Immunization to protect the US Armed Forces: heritage, current practice, and prospects.* Epidemiol Rev, 2006. **28**: p. 3-26.

44. Xue, F. and R.M. Burnett, *Capsid-like arrays in crystals of chimpanzee adenovirus hexon.* J Struct Biol, 2006. **154**(2): p. 217-21.

45. Farina, S.F., et al., *Replication-defective vector based on a chimpanzee adenovirus.* J Virol, 2001. **75**(23): p. 11603-13.

46. Tatsis, N., et al., *Chimpanzee-origin adenovirus vectors as vaccine carriers.* Gene Ther, 2006. **13**(5): p. 421-9.

47. Tatsis, N., et al., *A CD46-binding Chimpanzee Adenovirus Vector as a Vaccine Carrier.* Mol Ther, 2007.

48. Pinto, A.R., et al., *Induction of CD8+ T cells to an HIV-1 antigen through a prime boost regimen with heterologous E1-deleted adenoviral vaccine carriers.* J Immunol, 2003. **171**(12): p. 6774-9.

49. Reyes-Sandoval, A., et al., *Single-dose immunogenicity and protective efficacy of simian adenoviral vectors against Plasmodium berghei.* Eur J Immunol, 2008. **38**(3): p. 732-41.

50. Genton, B. and Z.H. Reed, *Asexual blood-stage malaria vaccine development: facing the challenges.* Curr Opin Infect Dis, 2007. **20**(5): p. 467-75.

51. Graves, P., H. Gelband, and P. Garner, *The SPf66 Malaria Vaccine: What is the Evidence for Efficacy?* Parasitol Today, 1998. **14**(6): p. 218-20.

52. Genton, B., et al., *Safety and immunogenicity of a three-component blood-stage malaria vaccine (MSP-1, MSP2, RESA) against Plasmodium falciparum in Papua New Guinean children.* Vaccine, 2003. **22**(1): p. 30-41.

53. Genton, B., et al., *A recombinant blood-stage malaria vaccine reduces Plasmodium falciparum density and exerts selective pressure on parasite populations in a phase 1-2b trial in Papua New Guinea.* J Infect Dis, 2002. **185**(6): p. 820-7.

54. Malkin, E.M., et al., *Phase 1 clinical trial of apical membrane antigen 1: an asexual blood-stage vaccine for Plasmodium falciparum malaria.* Infect Immun, 2005. **73**(6): p. 3677-85.

55. Dicko, A., et al., *Impact of a Plasmodium falciparum AMA1 Vaccine on Antibody Responses in Adult Malians.* PLoS ONE, 2007. **2**(10): p. e1045.

56. Dicko, A., et al., *Phase 1 study of a combination AMA1 blood stage malaria vaccine in Malian children.* PLoS ONE, 2008. **3**(2): p. e1563.

57. Mullen, G.E., et al., *Enhancement of functional antibody responses to AMA1-C1/Alhydrogel, a Plasmodium falciparum malaria vaccine, with CpG oligodeoxynucleotide.* Vaccine, 2006. **24**(14): p. 2497-505.

58. Mullen, G.E., et al., *Phase 1 trial of AMA1-C1/Alhydrogel plus CPG 7909: an asexual blood-stage vaccine for Plasmodium falciparum malaria.* PLoS ONE, 2008. **3**(8): p. e2940.

59. Polhemus, M.E., et al., *Phase I dose escalation safety and immunogenicity trial of Plasmodium falciparum apical membrane protein (AMA1) FMP2.1, adjuvanted with AS02A, in malaria-naive adults at the Walter Reed Army Institute of Research.* Vaccine, 2007. **25**(21): p. 4203-12.

60. Saul, A., et al., *A human phase 1 vaccine clinical trial of the Plasmodium falciparum malaria vaccine candidate apical membrane antigen 1 in Montanide ISA720 adjuvant.* Vaccine, 2005. **23**(23): p. 3076-83.

61. Roestenberg, M., et al., *Safety and immunogenicity of a recombinant Plasmodium falciparum AMA1 malaria vaccine adjuvanted with Alhydrogel, Montanide ISA 720 or AS02.* PLoS ONE, 2008. **3**(12): p. e3960.

62. Thompson, F.M., et al., *Evidence of Blood Stage Efficacy with a Virosomal Malaria Vaccine in a Phase IIa Clinical Trial.* PLoS ONE, 2008. **3**(1): p. e1493.

63. Sirima, S.B., et al., *Safety and immunogenicity of the Plasmodium falciparum merozoite surface protein-3 long synthetic peptide (MSP3-LSP) malaria vaccine in healthy, semi-immune adult males in Burkina Faso, West Africa.* Vaccine, 2007. **25**(14): p. 2723-32.

64. Prieur, E., et al., *A Plasmodium falciparum candidate vaccine based on a six-antigen polyprotein encoded by recombinant poxviruses.* Proc Natl Acad Sci U S A, 2004. **101**(1): p. 290-5.

65. Moorthy, V.S., et al., *Safety of DNA and modified vaccinia virus Ankara vaccines against liver-stage P. falciparum malaria in non-immune volunteers.* Vaccine, 2003. **21**(17-18): p. 2004-11.

66. Webster, D.P., et al., *Safety of recombinant fowlpox strain FP9 and modified vaccinia virus Ankara vaccines against liver-stage P. falciparum malaria in non-immune volunteers.* Vaccine, 2006. **24**(15): p. 3026-34.

67. Walther, M., et al., *Safety, immunogenicity, and efficacy of prime-boost immunization with recombinant poxvirus FP9 and modified vaccinia virus Ankara encoding the full-length Plasmodium falciparum circumsporozoite protein.* Infect Immun, 2006. **74**(5): p. 2706-16.

68. Hill, A.V., *Pre-erythrocytic malaria vaccines: towards greater efficacy.* Nat Rev Immunol, 2006. **6**(1): p. 21-32.

69. Webster, D.P., et al., *Enhanced T cell-mediated protection against malaria in human challenges by using the recombinant poxviruses FP9 and modified vaccinia virus Ankara.* Proc Natl Acad Sci U S A, 2005. **102**(13): p. 4836-41.

70. Wang, R., et al., *Induction of antigen-specific cytotoxic T lymphocytes in humans by a malaria DNA vaccine.* Science, 1998. **282**(5388): p. 476-80.

71. McConkey, S.J., et al., *Enhanced T-cell immunogenicity of plasmid DNA vaccines boosted by recombinant modified vaccinia virus Ankara in humans.* Nat Med, 2003. **9**(6): p. 729-35.

72. Moorthy, V.S., et al., *A randomised, double-blind, controlled vaccine efficacy trial of DNA/MVA ME-TRAP against malaria infection in Gambian adults.* PLoS Med, 2004. **1**(2): p. e33.

73. Anderson, R.J., et al., *Enhanced CD8+ T cell immune responses and protection elicited against Plasmodium berghei malaria by prime boost immunization regimens using a novel attenuated fowlpox virus.* J Immunol, 2004. **172**(5): p. 3094-100.

74. Bejon, P., et al., *Calculation of Liver-to-Blood Inocula, Parasite Growth Rates, and Preerythrocytic Vaccine Efficacy, from Serial Quantitative Polymerase Chain Reaction Studies of Volunteers Challenged with Malaria Sporozoites.* J Infect Dis, 2005. 191(4): p. 619-26.

Appendix A. Laboratory values for exclusion

The following reference ranges are provided for the purpose of guidance only for Investigators during the trial. Results during the trial that fall out with these ranges may not be of clinical significance but should be considered on an individual basis. Abnormal results judged of clinical significance should ordinarily be recorded as adverse events (AEs).

PARAMETER	LOWER LIMIT OF EXCLUSION	UPPER LIMIT OF EXCLUSION
**BIOCHEMISTRY**		
Potassium [mmol/L]	<3.2	>5.5
Sodium [mmol/L]	<132	>148
Urea [mmol/L]	N/A	>9
Creatinine [μmol/L]	N/A	>145
Albumin [g/L]	<30	N/A
Total bilirubin [μmol/L]	N/A	>19 when accompanied by elevated liver enzyme
ALT [IU/L]	N/A	>60
ALP [IU/L]	N/A	>350
**HAEMATOLOGY**		
Haemoglobin [g/dL]	Male: < 11.5<br>Female: < 10.5	Male: > 18<br>Female: >17.5
White Cell Count [x 10^9/L]<br>Neutrophil count [x 10^9/L]	<3.5<br>< 1.5	>14.0
Platelet Count [x 10^9/L]	<130	>500
**URINE ANALYSIS (using MULTISTIX * 10 SG Bayer Diagnostics)**		
Protein [g/L]	> 0.3	
Glucose [mmol/L]	> 5.5	
