# Supplementary material for: Phase Ia Clinical Evaluation of the Safety and Immunogenicity of the Plasmodium falciparum Blood-Stage Antigen AMA1 in ChAd63 and MVA Vaccine Vectors
Source: PLoS One. 2012 Feb 21;7(2):e31208. doi: 10.1371/journal.pone.0031208 (PMC3283618; doi:10.1371/journal.pone.0031208)
Supplement: Supplementary Information S1 — Inclusion and exclusion criteria for volunteers in study. (DOCX) [file pone.0031208.s012.docx]

**SUPPLEMENTAL INFORMATION**

**Inclusion Criteria**

- Healthy adults aged 18 to 50 years.
- Able and willing (in the Investigator’s opinion) to comply with all study requirements.
- Willing to allow the Investigators to discuss the volunteer’s medical history with their General Practitioner.
- For females only, willingness to practice continuous effective barrier contraception during the study and a negative pregnancy test on the day(s) of vaccination.
- For males only, willingness to use barrier contraception until three months after the last vaccination.
- Agreement to refrain from blood donation during the course of the study.
- Written informed consent.

**Exclusion Criteria**

- Participation in another research study involving an investigational product in the 30 days preceding enrolment, or planned use during the study period.
- Prior receipt of a recombinant adenoviral vaccine.
- Administration of immunoglobulins and/or any blood products within the three months preceding the planned administration of the vaccine candidate.
- Any confirmed or suspected immunosuppressive or immunodeficient state, including HIV infection; asplenia; recurrent, severe infections; and chronic (more than 14 days) immunosuppressant medication within the past 6 months (inhaled and topical steroids were allowed).
- History of allergic disease or reactions likely to be exacerbated by any component of the vaccine, e.g. egg products, Kathon.
- History of clinically significant contact dermatitis.
- Any history of anaphylaxis in reaction to vaccination.
- Pregnancy, lactation or willingness/intention to become pregnant during the study.
- History of cancer (except basal cell carcinoma of the skin and cervical carcinoma in situ).
- History of serious psychiatric condition.
- Any other serious chronic illness requiring hospital specialist supervision.
- Suspected or known current alcohol abuse as defined by an alcohol intake of greater than 42 units every week.
- Suspected or known injecting drug abuse.
- Seropositive for hepatitis B surface antigen (HBsAg).
- Seropositive for hepatitis C virus (antibodies to HCV).
- Any other significant disease, disorder or finding, which, in the opinion of the Investigator, may have either put the volunteer at risk because of participation in the study, or may have influenced the result of the study, or the volunteer’s ability to participate in the study.
- Any history of malaria .
- Travel to a malaria endemic region during the study period or within the previous six months.
- Any clinically significant abnormal finding on screening biochemistry or haematology blood tests or urinalysis.
- Any other finding which in the opinion of the Investigators would have significantly increased the risk of having an adverse outcome from participating in the protocol.

**Re-vaccination exclusion criteria**

- Anaphylactic reaction following administration of vaccine.
- Pregnancy.
