# Supplementary material for: Phase Ia Clinical Evaluation of the Safety and Immunogenicity of the Plasmodium falciparum Blood-Stage Antigen AMA1 in ChAd63 and MVA Vaccine Vectors
Source: PLoS One. 2012 Feb 21;7(2):e31208. doi: 10.1371/journal.pone.0031208 (PMC3283618; doi:10.1371/journal.pone.0031208)
Supplement: Table S1 — Assessment of severity of AEs. (PDF) [file pone.0031208.s013.pdf]

**Table S1. Assessment of severity of AEs.**

| Scale | Description | Definition |
|---|---|---|
| **0** | | Absence of the indicated symptom |
| **1** | Mild | Awareness of a symptom but the symptom is easily tolerated |
| **2** | Moderate | Discomfort enough to cause interference with usual activity |
| **3** | Severe | Incapacitating; unable to perform usual activities; requires absenteeism or bed rest |
