# Supplementary material for: Phase Ia Clinical Evaluation of the Safety and Immunogenicity of the Plasmodium falciparum Blood-Stage Antigen AMA1 in ChAd63 and MVA Vaccine Vectors
Source: PLoS One. 2012 Feb 21;7(2):e31208. doi: 10.1371/journal.pone.0031208 (PMC3283618; doi:10.1371/journal.pone.0031208)
Supplement: Table S2 — Assessment of severity of local AEs. (PDF) [file pone.0031208.s014.pdf]

Table S2. Assessment of severity of local AEs.

(A) Swelling & Erythema

Grade	Diameter (mm)
0	0
1	1 - 50
2	>50 – 100
3	> 100

(B) Pain

Grade	Description
**0**	No pain at all
**1**	Painful to touch, no restriction in movement of arms, able to work, drive, carry heavy objects as normal
**2**	Painful when limb is moved<br>(*i.e.* restriction in range of movement in arm, difficulty in carrying objects)
**3**	Severe pain at rest<br>(*i.e.* unable to use arm due to pain.)
