# Supplementary material for: Phase Ia Clinical Evaluation of the Safety and Immunogenicity of the Plasmodium falciparum Blood-Stage Antigen AMA1 in ChAd63 and MVA Vaccine Vectors
Source: PLoS One. 2012 Feb 21;7(2):e31208. doi: 10.1371/journal.pone.0031208 (PMC3283618; doi:10.1371/journal.pone.0031208)
Supplement: Table S3 — Assessment of relationship of AE to vaccination. (PDF) [file pone.0031208.s015.pdf]

Table S3. Assessment of relationship of AE to vaccination.

**0**	**No Relationship**	No temporal relationship to study product ***and***<br>Alternate aetiology (clinical state, environmental or other interventions); ***and***<br>Does not follow known pattern of response to study product
**1**	**Possible**	Reasonable temporal relationship to study product; ***or***<br>Event not readily produced by clinical state, environmental or other interventions; ***or***<br>Similar pattern of response to that seen with other vaccines
**2**	**Probable**	Reasonable temporal relationship to study product; ***and***<br>Event not readily produced by clinical state, environment, or other interventions ***or***<br>Known pattern of response seen with other vaccines
**3**	**Definite**	Reasonable temporal relationship to study product; ***and***<br>Event not readily produced by clinical state, environment, or other interventions; ***and***<br>Known pattern of response seen with other vaccines
