# Supplementary material for: Phase Ia Clinical Evaluation of the Safety and Immunogenicity of the Plasmodium falciparum Blood-Stage Antigen AMA1 in ChAd63 and MVA Vaccine Vectors
Source: PLoS One. 2012 Feb 21;7(2):e31208. doi: 10.1371/journal.pone.0031208 (PMC3283618; doi:10.1371/journal.pone.0031208)
Supplement: Table S4 — AMA1 overlapping peptides. 20mer peptides overlapping by 10 amino acids (aa) were generated for the whole of the AMA1 vaccine insert present in the ChAd63 and MVA vaccines. Peptides were divided into pools containing up to 10 peptides and were divided up according to whether they were 3D7 strain specific (3 pools, n = 24), FVO specific (3 pools, n = 24), common peptides (CP; 3 pools, n = 28), FVO terminus peptides (FVOT; 1 pool, n = 7). A single pool of tPA peptides (n = 5) was used and these were 15mers overlapping by 10aa. Amino acids that were substituted to prevent potential N-linked glycosylation are indicated in bold. (PDF) [file pone.0031208.s016.pdf]

Table S4. AMA1 overlapping peptides.

#	Sequence	Pool	#	Sequence	Pool
A1	QNYWEHPYQNSDVYRPINEH	3D71	A45	DNDDEGNKKIIAPRIFISDD	CP 3
A2	SDVYRPINEHREHPKEYEYP	3D71	A46	IAPRIFISDDKDSLKCPCDP	CP 3
A3	REHPKEYEYPLHQEHTYQQE	CP 1	A47	KDSLKCPCDPEMVS**Q**STCRF	CP 3
A4	LHQEHTYQQEDSGEDENTLQ	CP 1	A48	EMVS**Q**STCRFFVCKCVERRA	CP 3
A5	DSGEDENTLQHAYPIDHEGA	CP 1	A49	FVCKCVERRAEVTSNNEVVV	CP 3
A6	HAYPIDHEGAEPAPQEQNLF	CP 1	A50	EVTSNNEVVVKEEYKDEYAD	CP 3
A7	EPAPQEQNLFSSIEIVERSN	CP 1	A51	KEEYKDEYADIPEHKPTYDK	CP 3
A8	SSIEIVERSNYMGNPWTEYM	CP 1	A52	IPEHKPTYDKMKGGGPGGG	3D73
A9	YMGNPWTEYMAKYDIEEVHG	CP 1	A53	QNYWEHPYQKSDVYHPINEH	FVO1
A10	AKYDIEEVHGSGIRVDLGED	CP 1	A54	SDVYHPINEHREHPKEYEYP	FVO1
A11	SGIRVDLGEDAEVAGTQYRL	CP 1	A68	FL**T**PVATGNQDLKDGGFAFP	FVO1
A12	AEVAGTQYRLPSGKCPVFGK	CP 2	A69	DLKDGGFAFPPTNPLISPMT	FVO1
A13	PSGKCPVFGKGIIIENS**K**TT	CP 2	A70	PTNPLISPMTLNGMRDFYKN	FVO1
A14	GIIIENS**K**TTFLTPVATGNQ	CP 2	A71	LNGMRDFYKNNEYVKNLDEL	FVO1
A15	FLTPVATGNQYLKDGGFAFP	3D71	A72	NEYVKNLDELTLCSRHAGNM	FVO1
A16	YLKDGGFAFPPTEPLMSPMT	3D71	A73	TLCSRHAGNMNPDNDKNSNY	FVO1
A17	PTEPLMSPMTLDEMRHFYKD	3D71	A74	NPDNDKNSNYKYPAVYDYND	FVO2
A18	LDEMRHFYKDNKYVKNLDEL	3D71	A75	KYPAVYDYNDKKCHILYIAA	FVO2
A19	NKYVKNLDELTLCSRHAGNM	3D71	A77	QENNGPRYCNKDQSKRNSMF	FVO2
A20	TLCSRHAGNMIPDNDKNSNY	3D71	A78	KDQSKRNSMFCFRPAKDKLF	FVO2
A21	IPDNDKNSNYKYPAVYDDKD	3D72	A79	CFRPAKDKLFEN**LV**YLSKNV	FVO2
A22	KYPAVYDDKDKKCHILYIAA	3D72	A80	EN**LV**YLSKNVVDNWEEVCPR	FVO2
A23	KKCHILYIAAQENNGPRYCN	CP 2	A81	VDNWEEVCPRKNLENAKFGL	FVO2
A24	QENNGPRYCNKDESKRNSMF	3D72	A82	KNLENAKFGLWVDGNCEDIP	FVO2
A25	KDESKRNSMFCFRPAKDISF	3D72	A83	WVDGNCEDIPHVNEFSANDL	FVO3
A26	CFRPAKDISFQN**LV**YLSKNV	3D72	A84	HVNEFSANDLFECNKLVFEL	FVO3
A27	QN**LV**YLSKNVVDNWEKVCPR	3D72	A90	FKADRYKSHGKGYNWGNYNR	FVO3
A28	VDNWEKVCPRKNLQNAKFGL	3D72	A91	KGYNWGNYNRETQKCEIFNV	FVO3
A29	KNLQNAKFGLWVDGNCEDIP	3D72	A94	YIATTALSHPIEVEHNFPCS	FVO3
A30	WVDGNCEDIPHVNEFPAIDL	3D73	A95	IEVEHNFPCSLYKDEIKKEI	FVO3
A31	HVNEFPAIDLFECNKLVFEL	3D73	A96	LYKDEIKKEIERESKRIKLN	FVO3
A32	FECNKLVFELSASDQPKQYE	CP 2	A105	IPEHKPTYDNMKIIIASSAA	FVO3
A33	SASDQPKQYEQHLTDYEKIK	CP 2	A106	MKIIIASSAAVAVLATILMV	FVOT
A34	QHLTDYEKIKEGFKNKN**RE**M	CP 2	A107	VAVLATILMVYLYKRKGNAE	FVOT
A35	EGFKNKN**RE**MIKSAFLPTGA	CP 2	A108	YLYKRKGNAEKYDKMDQPQH	FVOT
A36	IKSAFLPTGAFKADRYKSHG	CP 2	A109	KYDKMDQPQHYGKSTSRNDE	FVOT
A37	FKADRYKSHGKGYNWGNYNT	3D73	A110	YGKSTSRNDEMLDPEASFWG	FVOT
A38	KGYNWGNYNTETQKCEIFNV	3D73	A111	MLDPEASFWGEEKRASHTTP	FVOT
A39	ETQKCEIFNVKPTCLIN**DKN**	CP 3	A112	EEKRASHTTPVLMEKPYY	FVOT
A40	KPTCLIN**DKN**YIATTALSHP	CP 3	tPA1	MDAMKRGLCCVLLLC	tPA
A41	YIATTALSHPIEVENNFPCS	3D73	tPA2	RGLCCVLLLCGAVFV	tPA
A42	IEVENNFPCSLYKDEIMKEI	3D73	tPA3	VLLLCGAVFVSPSQE	tPA
A43	LYKDEIMKEIERESKRIKLN	3D73	tPA4	GAVFVSPSQEIHARF	tPA
A44	ERESKRIKLNDNDDEGNKKI	CP 3	tPA5	SPSQEIHARFRR	tPA

20mer peptides overlapping by 10 amino acids (aa) were generated for the whole of the AMA1 vaccine insert present in the ChAd63 and MVA vaccines. Peptides were divided into pools containing up to 10 peptides and were divided up according to whether they were 3D7 strain specific (3 pools, *n* = 24), FVO specific (3 pools, *n* = 24), common peptides (CP; 3 pools, *n* = 28), FVO terminus peptides (FVOT; 1 pool, *n* = 7). A single pool of tPA peptides (*n* = 5) was used and these were 15mers overlapping by 10aa. Amino acids that were substituted to prevent potential N-linked glycosylation are indicated in bold.
