# Supplementary material for: Phase Ia Clinical Evaluation of the Safety and Immunogenicity of the Plasmodium falciparum Blood-Stage Antigen AMA1 in ChAd63 and MVA Vaccine Vectors
Source: PLoS One. 2012 Feb 21;7(2):e31208. doi: 10.1371/journal.pone.0031208 (PMC3283618; doi:10.1371/journal.pone.0031208)
Supplement: Table S5 — Vaccine versus Native peptides. 15mer peptide sequences are shown, and pools indicated: V = sequence found in vaccine insert (n = 21); N = sequence found in native parasite (n = 21). Amino acids that were substituted to prevent potential N-linked glycosylation are highlighted in bold. (PDF) [file pone.0031208.s017.pdf]

Table S5. Vaccine versus Native peptides.

#	Sequence	Pool	#	Sequence	Pool
31	VFGKGIIIENS**K**TTF	V	112	VFGKGIIIENS**N**TTF	N
32	IIIENS**K**TTFLTPVA	V	113	IIIENS**N**TTFLTPVA	N
33	S**K**TTFLTPVATGNQY	V	114	S**N**TTFLTPVATGNQY	N
56	FRPAKDISFQN**LV**YL	V	115	FRPAKDISFQN**YT**YL	N
57	DISFQN**LV**YLSKNVV	V	116	DISFQN**YT**YLSKNVV	N
58	N**LV**YLSKNVVDNWEK	V	117	N**YT**YLSKNVVDNWEK	N
73	EKIKEGFKNKN**RE**MI	V	118	EKIKEGFKNKN**AS**MI	N
74	GFKNKN**RE**MIKSAFL	V	119	GFKNKN**AS**MIKSAFL	N
75	N**RE**MIKSAFLPTGAF	V	120	N**AS**MIKSAFLPTGAF	N
83	IFNVKPTCLIN**DKN**Y	V	121	IFNVKPTCLIN**NSS**Y	N
84	PTCLIN**DKN**YIATTA	V	122	PTCLIN**NSS**YIATTA	N
85	N**DKN**YIATTALSHPI	V	123	N**NSS**YIATTALSHPI	N
98	DSLKCPCDPEMVS**Q**S	V	124	DSLKCPCDPEMVS**N**S	N
99	PCDPEMVS**Q**STCRFF	V	125	PCDPEMVS**N**STCRFF	N
100	MVS**Q**STCRFFVCKCV	V	126	MVS**N**STCRFFVCKCV	N
32	IIIENS**K**TTFL**T**PVA	V	194	IIIENS**N**TTFL**K**PVA	N
147	S**K**TTFL**T**PVATGNQD	V	195	S**N**TTFL**K**PVATGNQD	N
148	L**T**PVATGNQDLKDGG	V	196	L**K**PVATGNQDLKDGG	N
167	FRPAKDKLFEN**LV**YL	V	197	FRPAKDKLFEN**YT**YL	N
168	DKLFEN**LV**YLSKNVV	V	198	DKLFEN**YT**YLSKNVV	N
169	N**LV**YLSKNVVDNWEE	V	199	N**YT**YLSKNVVDNWEE	N

15mer peptide sequences are shown, and pools indicated: V = sequence found in vaccine insert (n = 21); N = sequence found in native parasite (n = 21). Amino acids that were substituted to prevent potential N-linked glycosylation are highlighted in bold.
